# Formal Total Synthesis of (±)-Isoalantolactone

**DOI:** 10.1021/acs.orglett.6c03173

**Published:** 2026-08-19

**Authors:** Wilma Olsson, Johan Wennerberg, Sebastian Clementson

**Affiliations:** † Centre for Analysis and Synthesis, Department of Chemistry, Lund University, SE-221 00 Lund, Sweden; ‡ RG Discovery, Medicon Village SE-221 81 Lund, Sweden

## Abstract

Herein, we report a concise formal synthesis of (±)-isoalantolactone through a six-step route to the advanced intermediate of Tada. The 5/6/6-fused eudesmanolide core is assembled through a SmI_2_-mediated reductive lactonization, while a late-stage acid-promoted sequence combines global deprotection, intramolecular aldol condensation, and thermodynamic stereochemical convergence to establish the required relative stereochemistry. Computational analysis supports thermodynamically controlled formation of the observed diastereomer and validates the overall synthetic design.

## Graphical abstract

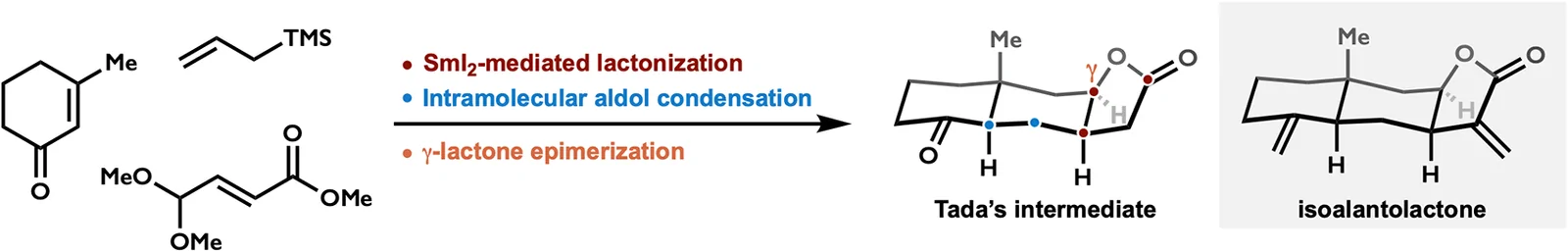

Among sesquiterpene lactones, the eudesmanolides constitute a structurally diverse family of natural products that has attracted sustained interest from both the biological and synthetic communities (Figure 1).^1^ Isolated primarily from plants of the *Compositae* family, these structurally diverse metabolites are associated with a broad spectrum of biological activities. Isoalantolactone (**1**) and alantolactone (**2**), the major constituents of *Inula Helenium* ^2^ and *Inula Racemosa* ^3^ are representative members of this class and have shown potent anticancer activity against a range of cancer types, including lung, colorectal, and breast cancer.^4–6^ Among the reported mechanisms, they induce apoptosis by downregulating the PI3K/AKT/mTOR signaling pathway.^7,8^ Several studies further suggest that they synergistically enhance the efficacy of traditional chemotherapeutic agents such as cisplatin,^9,10^ olaparib,^11^ and oxaliplatin.^12^ Derivatization at the C2 and C15/C16 positions has also furnished analogues with attenuated cytotoxicity and retained or augmented bioactivity,^13–18^ as illustrated by hydroxamic acid hybrids of isoalantolactone that function as potent STAT3/HDAC inhibitors with improved cellular uptake.^19^

**Figure 1:**
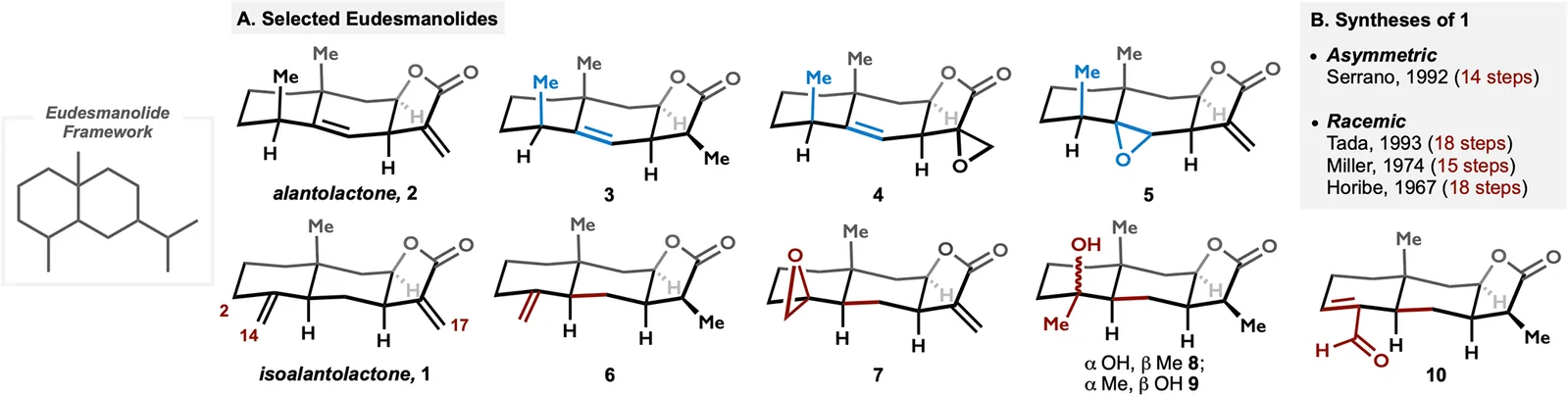
A) Selected eudesmanolides:^3^ alantolactone (**2**) and structurally related compounds (**blue**, **3**–**5**); isoalantolactone (**1**) and structurally related compounds (**red**, **6**–**10**). B) Prior synthetic approaches to isoalantolactone (**1**).

Despite these advances, the scarcity of these natural products has meant that analogue development has largely relied on semisynthesis, thereby limiting systematic exploration of closely related alantolactone (**blue**) and isoalantolactone (**red**)-type congeners (Figure 1).^20^ In an effort to expand the eudesmanolide chemical space, several strategies toward **1** and **2** have been reported (Figure 1 **.B**). As recently as 2025, Larach et al. disclosed a divergent approach targeting alantolactone (**2**) alongside structurally related eudesmanolides, rendering the alantolactone-type scaffold readily accessible.^21^ In contrast, efficient access to its isomer isoalantolactone (**1**) remains underdeveloped. Dating back to 1967, a few strategies toward isoalantolactone (**1**) have been reported, including semisynthetic (from (−)-santonin^22^) and formal/total synthetic approaches^23–25^ (Figure 1 **B**). Nevertheless, despite these efforts, step-efficient and stereoselective access to the isoalantolactone scaffold remains limited.

Herein, we report a concise formal synthesis of isoalantolactone (**1**) by means of a SmI_2_-mediated reductive lactonization. Subsequent acid-promoted aldol condensation provides rapid access to the eudesmanolide 5/6/6 tricyclic core while simultaneously unveiling an unusual isomerization that converges all stereoisomers under thermodynamic control. With only six steps to a known advanced intermediate, this route represents the most step-economical approach to isoalantolactone (**1**) reported to date.

We initiated our synthetic efforts by exploring Giese additions to a α-methylene-γ-butyrolactone **11** (Scheme 1 **.A**). Employing a mild photocatalytic β–β-coupling strategy developed in our laboratory,^26^ ring-opening of cyclopropyl **12** generated β-radical **13**, which underwent efficient Giese addition to **11** under blue LED irradiation, and furnished **14** in 68% yield. With **14** in hand, we then sought to forge the remaining ring system through hydrogenation followed by aldol condensation. Despite extensive screening, these efforts proved unsuccessful – a limitation we attribute to the steric demands imposed by the tetrasubstituted olefin.

**Scheme 1:**
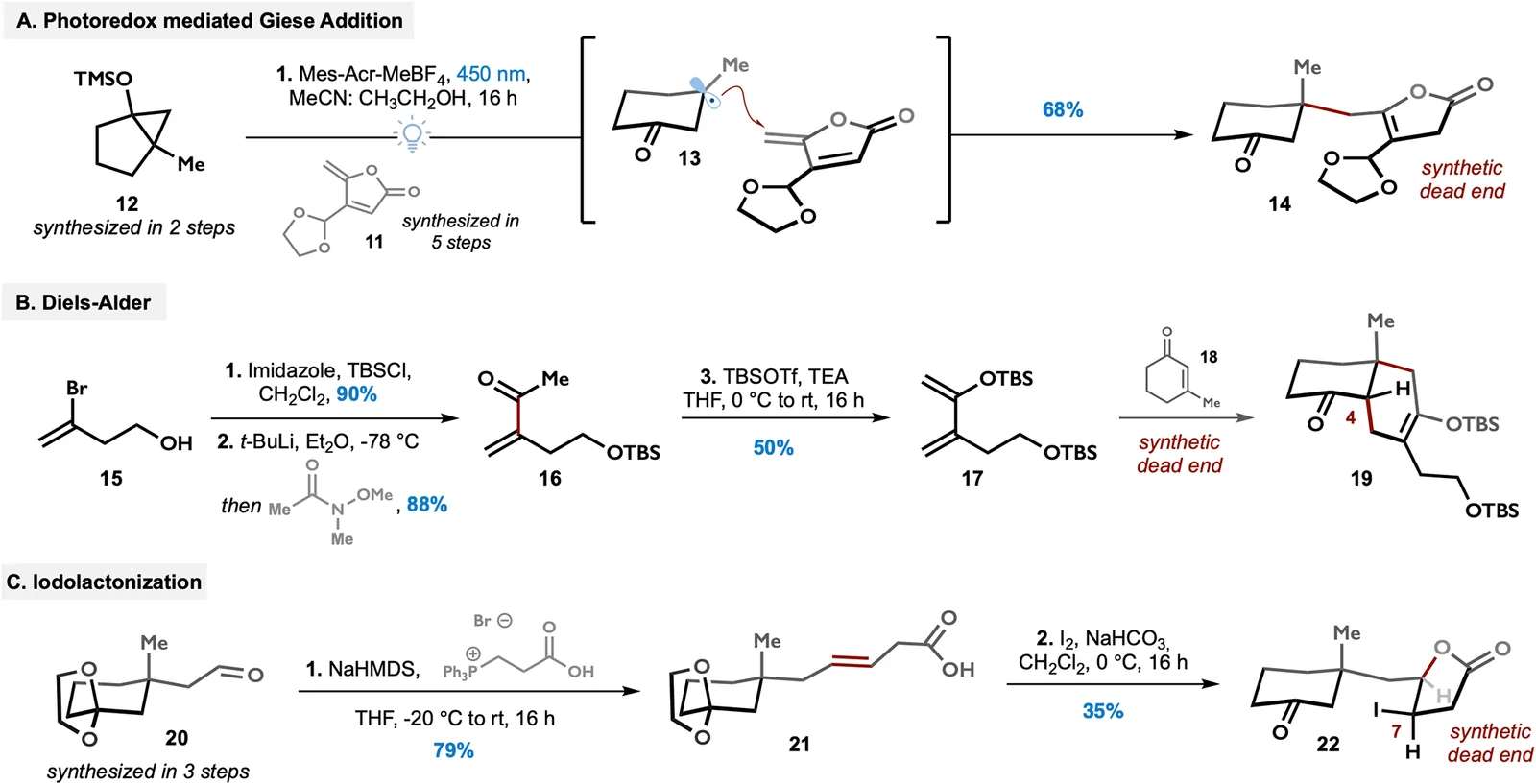
Attempted Synthetic Routes towards Isoalantolactone (**1**): A) Photoredox-Mediated Giese Addition, B) Diels-Alder, and C) Iodolactonization

Seeking a retrosynthetically distinct approach, we then envisioned a Diels–Alder reaction between enol ether **17** and 2-methylcyclohexenone (**18**) to effectively install the 6/6 fused framework of **19** (Scheme 1 **.B**). Encouraged by the work of Jung and Guzaev^27^ as well as Stoltz and co-workers,^28^ we anticipated efficient installation of the quaternary center of the fused ring followed by epimerization at C4 into a trans-decalin scaffold and redox manipulations en route to the desired lactone. Although diene **17** was successfully prepared in 3 steps from **15**, the desired Diels–Alder product (**19**) remained elusive despite extensive screening of additives and protecting groups, likely due to the enol ether lability in combination with the steric demands of the cyclization.

We therefore turned our attention to an iodolactonization approach (Scheme 1 **.C**). Aldehyde **20**, prepared in 3 steps from 2-methylcyclohexenone, was subjected to a Wittig olefination to deliver olefin **21** in 79% yield.^29^ Treatment of **21** with I_2_ and NaHCO_3_ promoted the key iodolactonization with concomitant acetal removal,^30,31^ furnishing **22**. Subsequent attempts of C7 functionalization from this intermediate, however, proved unsuccessful, preventing further advancement toward isoalantolactone (**1**).

Recognizing the limitations of our earlier approaches, we then sought to exploit aldehyde **20** in a redesigned Giese addition. Commencing from readily available **18**, Hosomi-Sakurai allylation furnished **23** in 56% yield^32^ (Scheme 2 **.A**). Acetal protection and subsequent Lemieux-Johnson oxidation^33^ then furnished aldehyde **20** and set the stage for the key SmI_2_-mediated lactonization.^34–37^ Single-electron reduction of the aldehyde generated an α-oxy radical that underwent Giese addition to acrylate **24**, which upon additional reduction spontaneously lactonized to furnish **25** as a mixture of diastereomers. Careful control of time and temperature proved critical to minimize the formation of side products. Photocatalytic generation of the α-hydroxy radical following MacMillan’s protocol^38^ was also explored but only resulted in trace amounts of product (Scheme 2 **.B**).

**Scheme 2:**
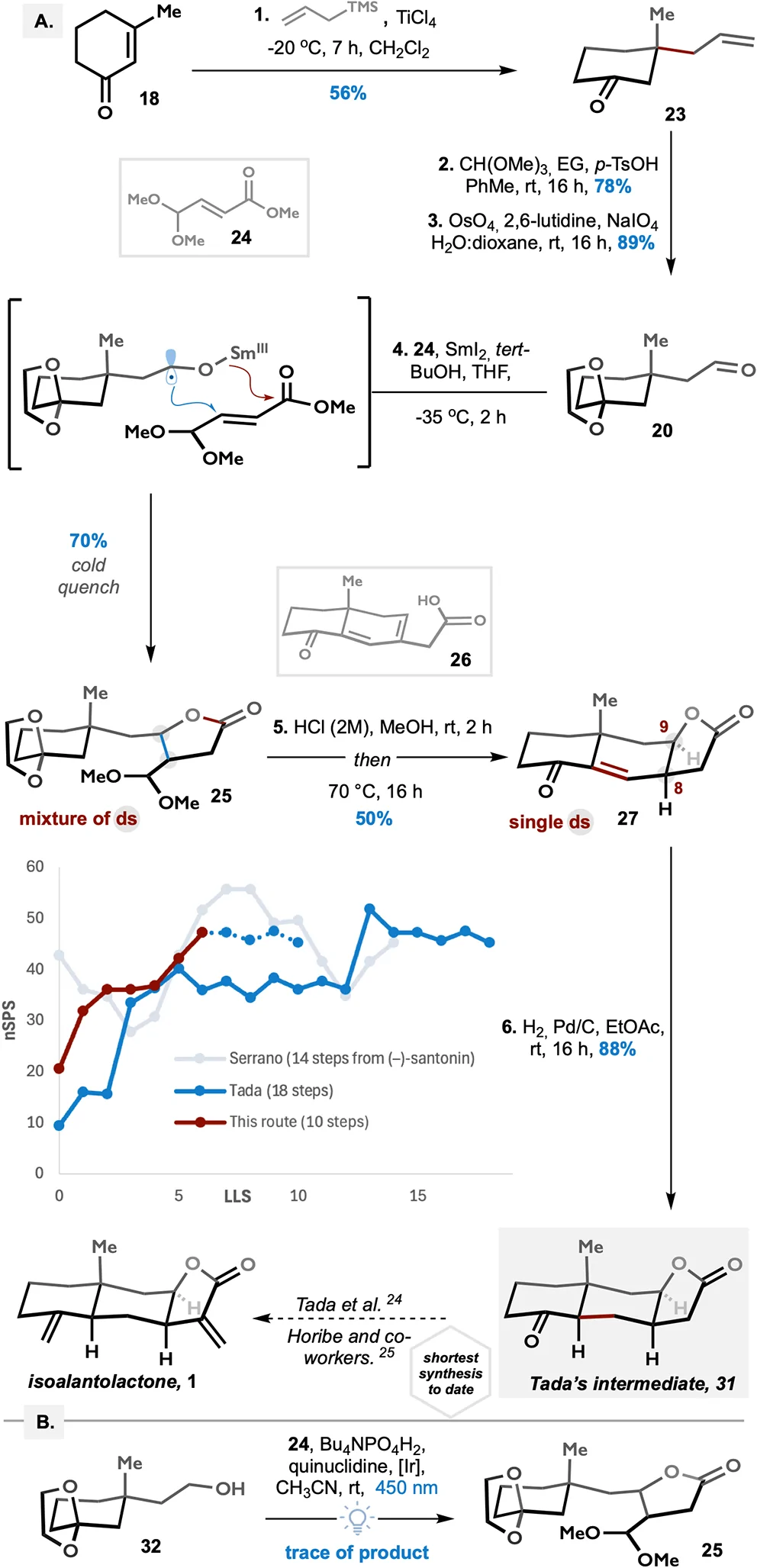
A) Formal Synthesis of (±)-Isoalantolactone (**1**) and B) Alternative Lactonization Approach

Treatment of **25** with 2 M HCl initiated a highly productive late-stage sequence in which both acetals were removed, the tricyclic eudesmanolide core was forged by intramolecular aldol condensation, and the initially formed diastereomeric mixture converged to the desired diastereomer under thermodynamic control.

Although a γ-lactone epimerization has been observed in a study with (−)-santonin, where the rearrangement proceeds through a benzylic carbocation,^39^ we are not aware of a corresponding nonbenzylic example. We therefore propose that the present mechanism proceeds through **26**, ultimately favoring the thermodynamically preferred diastereomer (Scheme 2.A). We anticipate that this epimerization may prove useful in related γ-lactone systems. Notably, C9 requires strong acid to epimerize, while C8 seems to epimerize under milder acidic conditions (see SI for details).

The experimental findings are further supported by computational analysis, where compound **27** is identified as the most stable diastereomer across all examined solvents (Table 1). The energy difference between the desired **27** and the remaining diastereomers (>1.4 kcal/mol) rationalizes the selective formation of compound **27** under thermodynamic control. Consistent with their large relative energies (>6 kcal/mol), no trace of the trans-fused configurations **29** and **30** were observed. Notably, compound **28** is marginally more stable in the gas phase (Δ*G* = −0.56 kcal/mol), highlighting the critical role of solvation in governing the observed diastereoselectivity.

**Table 1:**
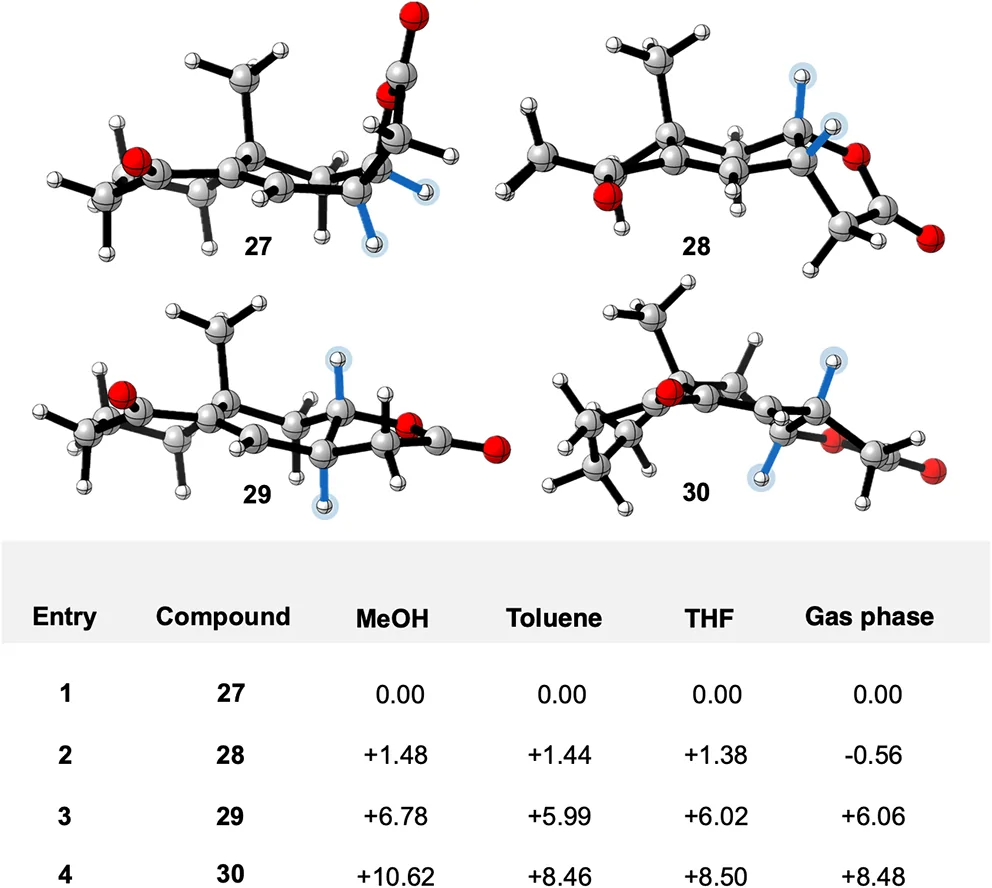
Relative Gibbs Free Energy, Δ*G* (kcal/mol), of Compounds **28**–**30** Relative to Compound **27** in MeOH, Toluene, THF, and the Gas Phase^a^

a Calculated at the M06-2X/def2-TZVP//M06-2X/def2-SVP level of theory with SMD implicit solvation model. The structures have been geometry optimized in MeOH.

With the eudesmanolide core **27** in hand, hydrogenation over Pd/C stereoselectively furnished the desired compound **31** in 88% yield, thereby completing the formal synthesis. The hydrogenated product **31** can then be readily converted to (±)-isoalantolactone (**1**) according to the protocol by Tada et al.^24^ and Horibe and co-workers.^25^ The synthetic efficiency was assessed by plotting normalized Spacial Score (nSPS) values^40^ against step count (LLS). As seen in Scheme 2 **.A**, our synthetic route efficiently builds up molecular complexity throughout the sequence and provides the shortest route of isoalantolactone (**1**) to date.

In conclusion, we disclose a concise formal synthesis of isoalantolactone (**1**) featuring a key SmI_2_-mediated lactonization and late-stage acid-promoted stereochemical convergence to the desired eudesmanolide core. This advanced intermediate, reached in only six steps, provides a platform to further explore the eudesmanolide framework.

## Supplementary Material



## Data Availability

The data underlying this study are available in the published article and its Supporting Information.

## Notes

Materials and methods, experimental procedures, computational details, and characterization data (PDF)
