# Supplementary material for: Formal Total Synthesis of (±)-Isoalantolactone
Source: Org Lett. 2026 Aug 19;28(34):10859–63. doi: 10.1021/acs.orglett.6c03173 (PMC13532190; doi:10.1021/acs.orglett.6c03173)
Supplement: Supplementary file 1 [file ol6c03173_si_001.pdf]

Supporting Information

Formal Total Synthesis of (±)-Isoalantolactone

Wilma Olsson[ab], Johan Wennerberg[ab], and Sebastian Clementson*[a]

a) Centre for Analysis and Synthesis, Department of Chemistry, Lund University Box 124, SE-221 00 Lund, Sweden.
E-mail: sebastian.clementson@chem.lu.se
b) RG Discovery, Medicon Village, SE-223 81, Lund, Sweden.

Table of Content

I. General Procedures 3
II. Computational Modeling 6
General Details 6
Calculated Energies 6
III. Experimental Section 7
IV. Supplementary References 16
V. Copies of ^{1}H, ^{13}C and 2D NMR of Compounds **S3-S4, 16-17, 20-25**, **27-28**, and **31-32** 18
VI. Cartesian Coordinates of Computed Structures 35

SAFETY STATEMENT: No unusual or unexpected safety issues were encountered throughout this work.

I. General Procedures

Synthesis, general: Unless otherwise stated, all reactions were carried out in oven-dried glassware under a dry N_2 atmosphere using standard Schlenk techniques or in a N_2 filled glovebox (O_2, H_2O < 1 ppm). Reactions were heated using a metal block on a hotplate with feedback temperature control, unless otherwise stated. Room temperature (rt) refers to temperatures in a range between 20 – 22 °C. Mixtures were concentrated under reduced pressure by rotary evaporation. For known compounds (references given) synthesized through new or modified procedures, copies of ^1H NMR and $^{13}C\{^1H\}$ NMR spectra are provided. Yields refer to chromatographically and spectroscopically (^1H NMR and $^{13}C\{^1H\}$ NMR) homogeneous material, unless otherwise stated.

Reagents and solvents: Anhydrous THF, CH_2Cl_2, Et_2O, and toluene were obtained from a solvent purification system equipped with alumina columns. Anhydrous EtOAc and MeOH were dried over activated molecular sieves (3 or 4 Å) for at least 16 h prior to use. Petroleum ether (PE) refers to the fraction with boiling point 40 – 60 °C. All other solvents and reagents were purchased from commercial suppliers and used as received without further purification.

NMR spectroscopy: NMR spectra were recorded on a Bruker Ultrashield 400 plus (^1H at 400 MHz and $^{13}C\{^1H\}$ at 101 MHz), Bruker Avance II (^1H at 500 MHz and $^{13}C\{^1H\}$ at 126 MHz), Bruker Avance Neo spectrometer (^1H at 500 MHz and $^{13}C\{^1H\}$ at 126 MHz), or on a Varian Inova (^1H at 400 MHz and $^{13}C\{^1H\}$ at 101 MHz), and processed using MestReNova version 14.2.0. Chemical shifts (δ) are reported in parts per million (ppm) and referenced to the residual solvent signal for $CDCl_3$ (δ = 7.26 ^1H; δ = 77.16 $^{13}C\{^1H\}$). Coupling constants (*J*) are given in hertz. ^1H NMR spectra are reported as follows: chemical shift, multiplicity (s = singlet, d = doublet, t = triplet, q = quartet, sept. = septet, dd = doublet of doublets, dt = doublet of triplets, m = multiplet, br. = broad, app. = apparent), coupling constant, and integration. $^{13}C\{^1H\}$ NMR spectra are reported as follows: chemical shift. When two or fewer signals overlap in ^{13}C NMR, these signals are clearly noted. If three or more signals overlap, this is written as *multiple overlapping signals*. Structural assignments were made with additional information from gCOSY, edited gHSQC, and 1D NOESY experiments.

Chromatography: Thin layer chromatography (TLC) was performed using aluminum-backed plates (Merck $60F_{254}$ silica gel). The plates were visualized with UV-light (254 nm) and/or stained with alkaline $KMnO_4$ or anisaldehyde stain. Flash column chromatography was performed using silica gel (Thermo Scientific, 60 – 200 µm, 60 Å) or aluminum oxide (Thermo Scientific, Brockmann I, 60 Å, neutral or basic).

Mass spectrometry: Mass spectra (HRMS) were recorded on a Micromass Q-TOF spectrometer (ESI) or on a LTQ Velos PRO orbitrap mass spectrometer (Thermo Scientific) (ESI).

IR spectroscopy: IR spectra were recorded on a Bruker ALPHA-P in ATR mode and selected peaks are reported as follows: wavenumber (cm^{-1}).

Photoreactor setup: All photochemical reactions were irradiated using a PennOC/PennPhD Integrated Photoreactor (IPR). For these experiments, the 450 nm blue LED module (M2 photoreactor) was employed. Unless otherwise noted, reactions were stirred at 650 rpm and cooled using the maximum fan speed setting throughout irradiation. Under these conditions, reaction vessels were maintained near rt (approximately 27 °C) for the duration of irradiation.

Complexity quantification: Molecular complexity was quantified using the Spacial Score metric developed by Krzyanowski *et al.*[1] The scores were determined by inserting the molecular structures SMILES strings obtained from Chemdraw into the MolComplex web tool (https://www.molcomplex.org) developed by the Sarpong group.[2] To mitigate protecting group impact, the values were then normalized (nSPS) by dividing the SPS scores with the number of heavy atoms (non-hydrogen atoms). The resulting complexity values were then plotted against the synthetic step count (longest linear sequence, LLS). To allow for a fair

comparison of routes, a synthetic step is defined as proposed by Johnson throughout the following analysis.[3] Here, any type of solvent switch, workup, and/or filtration mark the beginning of a new step.

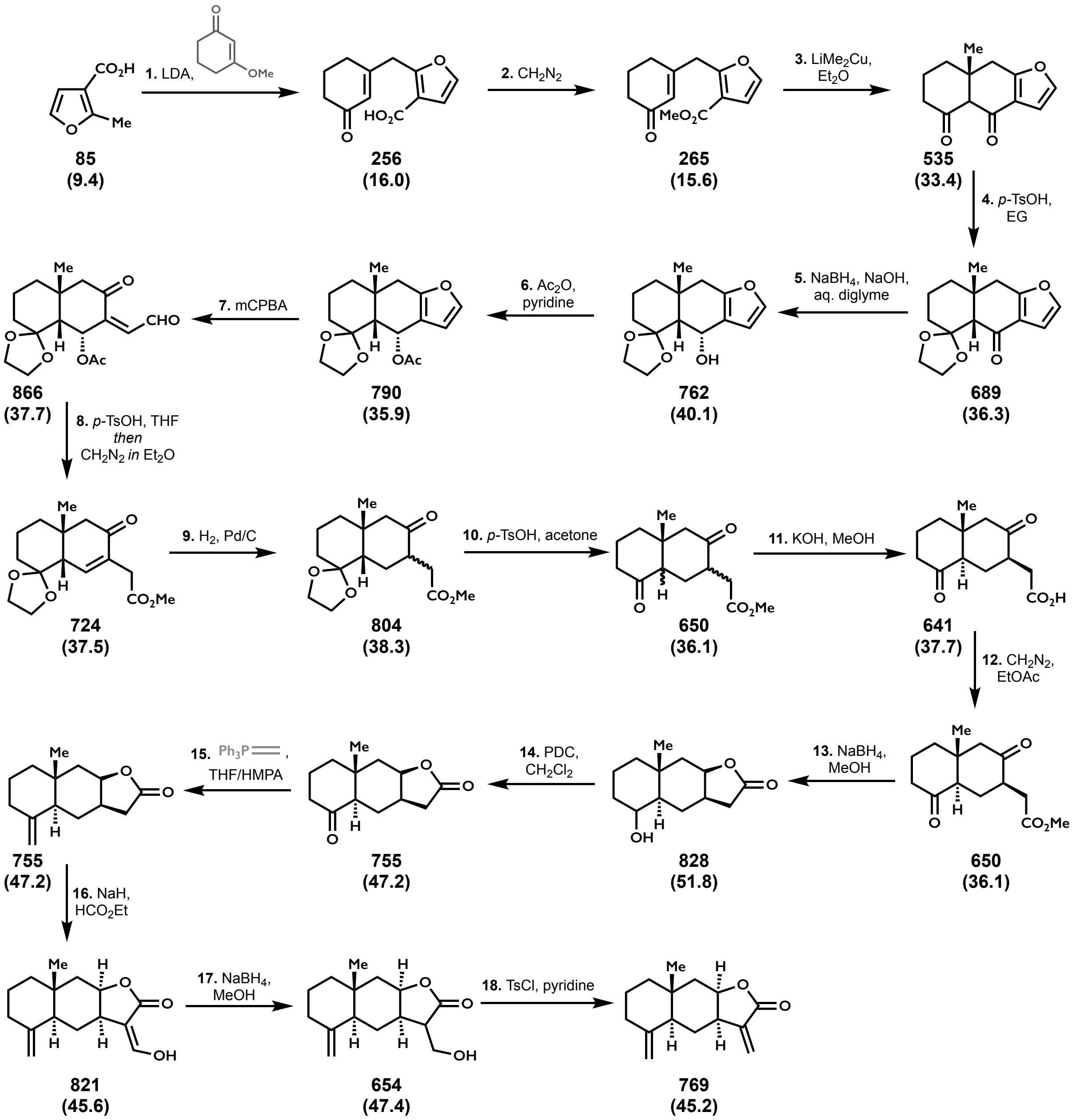

Figure S1. Complexity analysis of the formal total synthesis by Tada *et al.*,[4] including SPS values (above) and nSPS values (below, in parenthesis). Steps 16-18 were performed according to the procedure by Horibe and co-workers.[5]

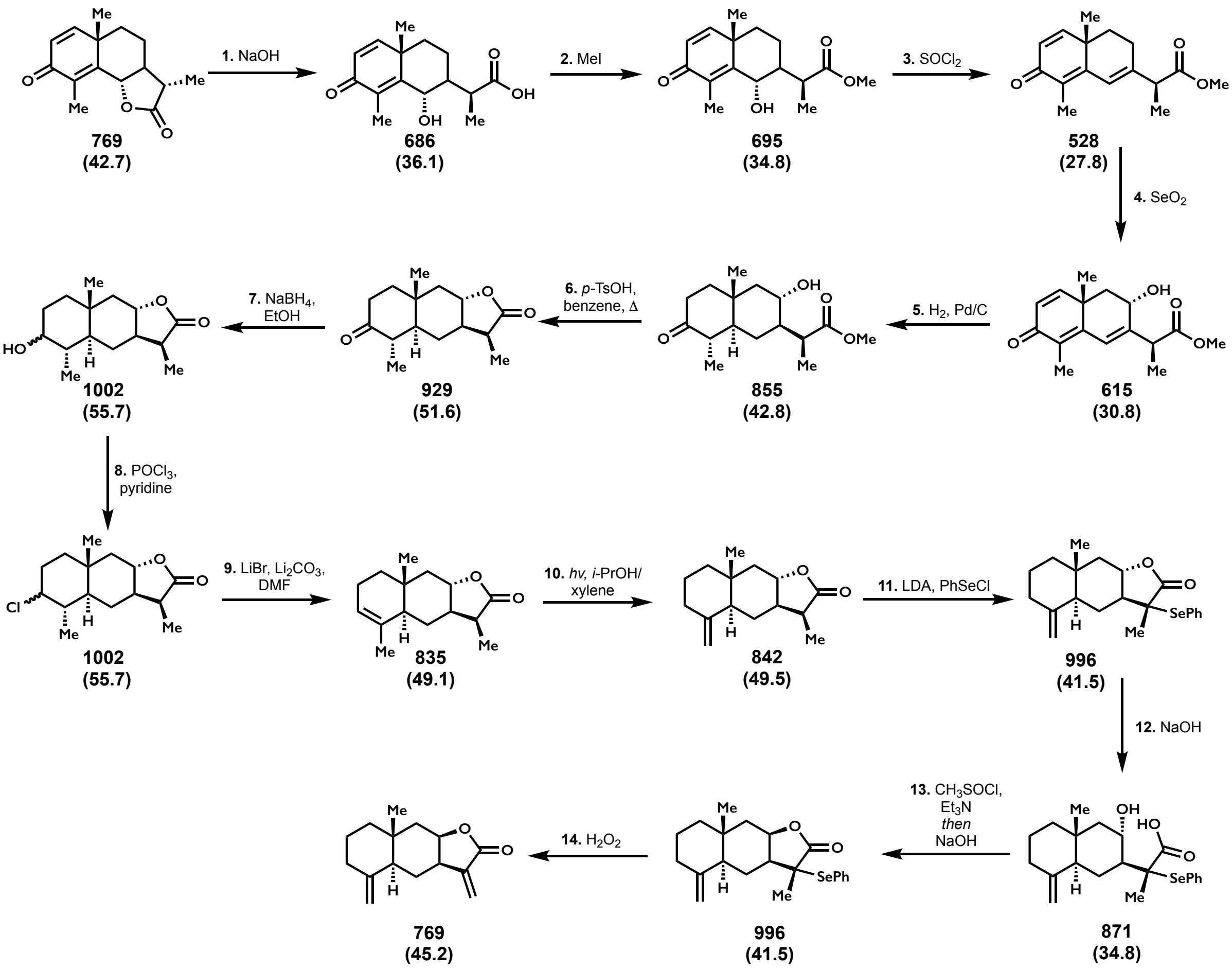

Figure S2. Complexity analysis of the synthesis by Serrano and co-workers,[6,7] including SPS values (above) and nSPS values (below, in parenthesis).

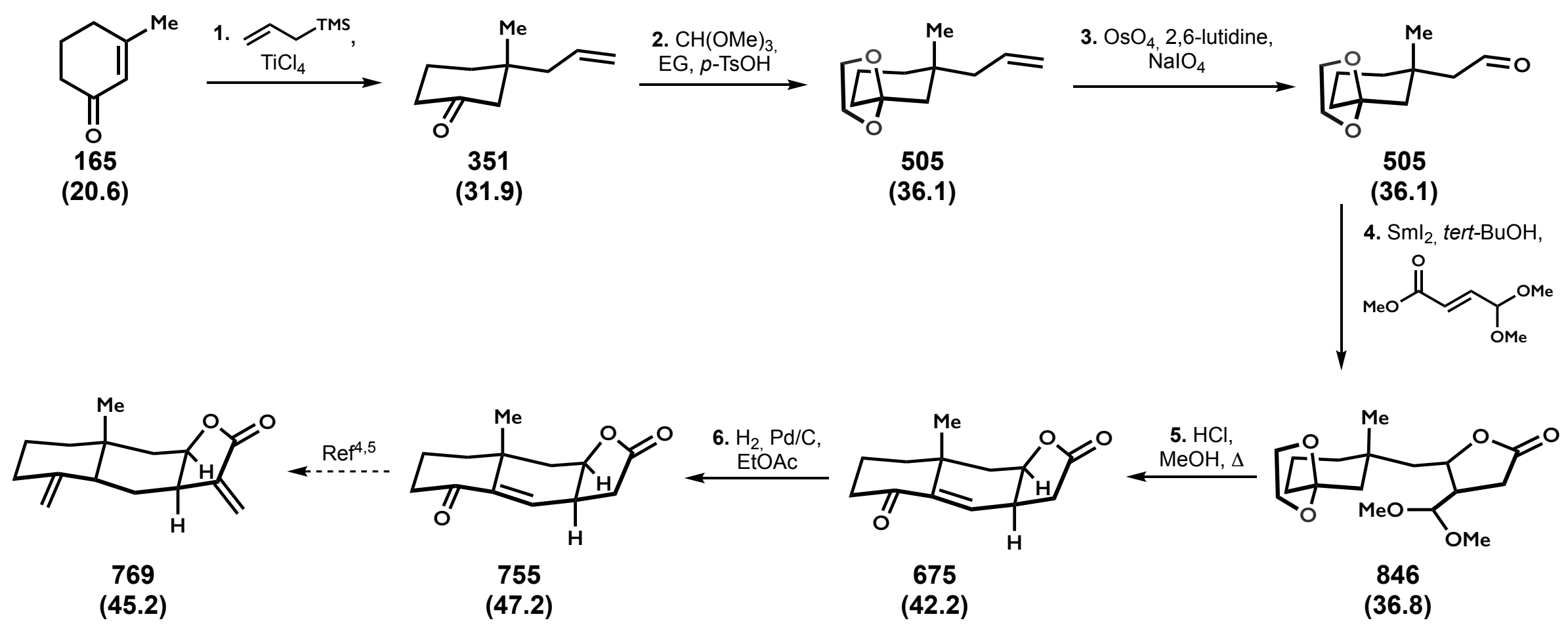

Figure S3. Complexity analysis of our formal total synthesis, including SPS values (above) and nSPS values (below, in parenthesis).

II. Computational Modeling

General Details

All quantum chemical calculations were carried out with the ORCA v.6.1.0 software.[8] Initial geometries were generated through force field-based optimization using MMFF94.[9] Conformational searches were performed with GOAT ensemble generator[10] using GFN2-xTB developed by Grimme and co-workers.[11] Optimization of molecular geometries were performed at the M06-2X/def2-SVP/def2/J level of theory for all atoms.[12–16] Frequency calculations at the same level of theory was then used to confirm the stationary points as true minima on the potential energy surface (characterized by zero imaginary frequencies). Energies were then further refined through single point calculations at the M06-2X/def2-TZVP/def2/J auxiliary level of theory for all atoms. To model the reactions conducted in solvent, the SMD[17] implicit solvation model for MeOH, toluene and THF was incorporated in the geometry optimization, frequency analysis and single-point energy calculations. To reduce integration grid errors, the ORCA implemented integration grid DefGrid3 was used in all calculations.[18] The TightSCF criteria was also implemented. Structures corresponding to the reactions conducted in MeOH were then visualized using CYLview.[19]

Calculated Energies

Table S1. Calculated energies of structure **27** - **30** given in Hartrees. **ΔG** represents the Gibbs free energy correction, **E** the single point electronic energy, and **G** the final Gibbs free energy. The SMD implicit solvation model was used to model the reactions run in MeOH, toluene, or THF. The dash (–) represents gas phase calculations. The functional, basis set and auxiliary are shown in brackets.

STRUCTURE	SOLVENT	ΔG [M06-2X/DEF2-SVP/DEF2/J]	E [M06-2X/DEF2-TZVP/DEF2/J]	G = ΔG + E
**27**	MeOH	0.23368123	-730.7050063	-730.4713251
	Toluene	0.2340132	-730.6984747	-730.4644615
	THF	0.23371696	-730.7031413	-730.4694243
	–	0.23433297	-730.677208	-730.442875
**28**	MeOH	0.2329364	-730.7019044	-730.468968
	Toluene	0.23375108	-730.6959223	-730.4621712
	THF	0.23340018	-730.7006301	-730.4672299
	–	0.23412252	-730.6778859	-730.4437634
**29**	MeOH	0.23376625	-730.6947673	-730.461001
	Toluene	0.23420636	-730.6891187	-730.4549123
	THF	0.23400516	-730.6938316	-730.4598265
	–	0.23429873	-730.6675193	-730.4332206
**30**	MeOH	0.23454241	-730.6889424	-730.4544
	Toluene	0.23392562	-730.6849022	-730.4509766
	THF	0.23371716	-730.689589	-730.4558719
	–	0.23411079	-730.6634763	-730.4293655

III. Experimental Section

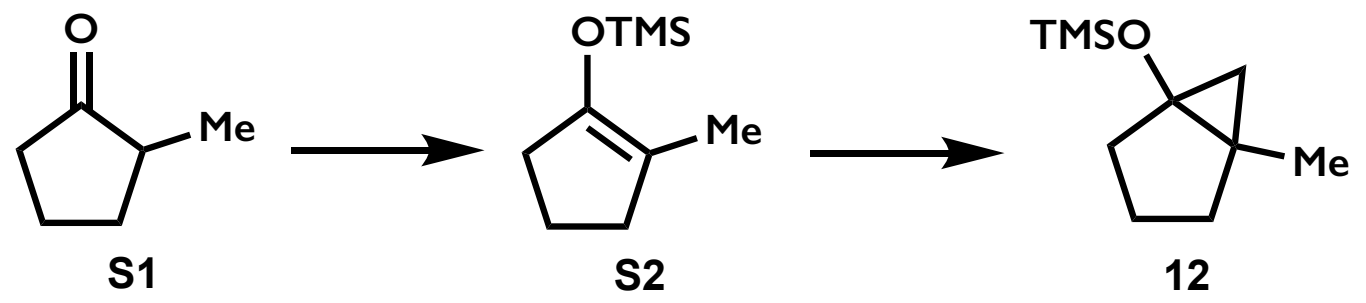

Cyclopropyl silyl ether 12. Silyl enol ether **S2** was prepared from 2-methylcyclopentanone (**S1**) according to the one-step procedure reported by Nicolaou *et al.*[20] The crude silyl enol ether was then used to prepare cyclopropyl silyl ether **12** according to the cyclopropanation procedure previously described by our group.[21] Analytical data (^{1}H, ^{13}C NMR, HRMS) were in agreement with those previously described.[22]

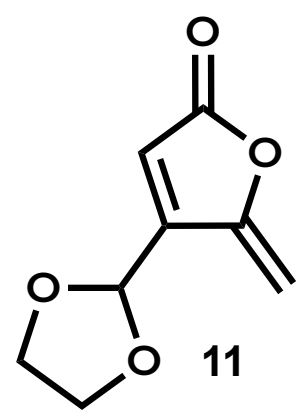

Butenolide 11. Butenolide **11** was prepared from 1,3-dihydroxyacetone according to the five-step procedure previously described by our group.[21] Analytical data (^{1}H, ^{13}C NMR, HRMS) were in agreement with those previously described.[21]

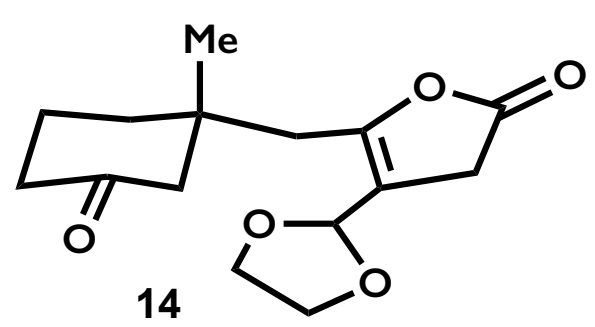

1,6-addition product 14. Lactone **14** was prepared from **11** and **12** according to the procedure reported by our group. Analytical data (^{1}H, ^{13}C NMR, HRMS) were in agreement with those previously described.[21]

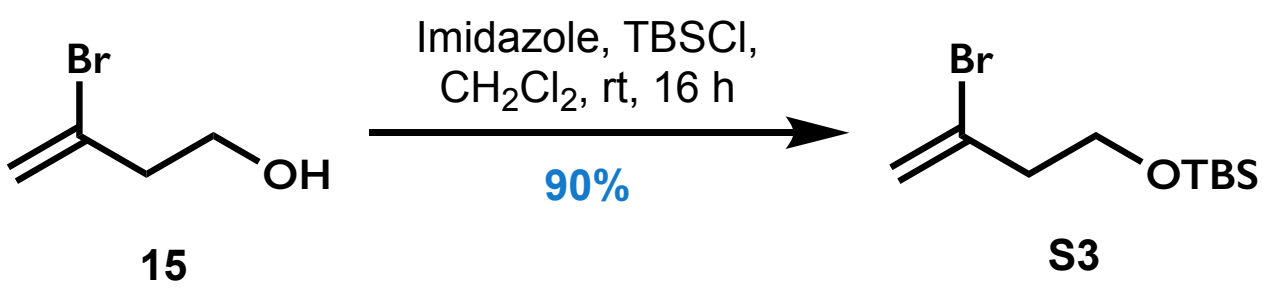

Silyl ether S3. Under an inert atmosphere at 0 °C, a solution of TBSCl (3.26 g, 21.6 mmol) in dry CH_2Cl_2 (15 mL) was added dropwise over 15 min to 3-bromo-3-buten-1-ol (**15**, 2.20 g, 14.6 mmol) and imidazole (1.49 g, 21.9 mmol) in dry CH_2Cl_2 (15 mL). The mixture was stirred for 16 h at rt, quenched with H_2O (25 mL), extracted with CH_2Cl_2 (3 x 25 mL), and dried over $NaSO_4$. Concentration under reduced pressure and purification by flash chromatography (silica gel, 5 to 20% EtOAc in PE) afforded the silylated bromo-vinyl **S3** (3.50 g, 13.2 mmol, 90%) as a colorless oil.

R$_f$ 0.62 (silica gel, 10% EtOAc in *n*-heptane).

^{1}H NMR (400 MHz, $CDCl_3$) δ 5.63 (d, *J* = 1.6 Hz, 1H), 5.46 (d, *J* = 1.5 Hz, 1H), 3.78 (t, *J* = 6.3 Hz, 2H), 2.62 (t, *J* = 6.3 Hz, 2H), 0.89 (s, 9H), 0.07 (s, 6H).

^{13}C{^{1}H} NMR (126 MHz, $CDCl_3$) δ 131.0, 118.6, 61.0, 44.9, 26.0, 18.5, -5.2.

HRMS (ESI+) *m/z:* calcd. for $C_{10}H_{22}BrOSi^+$ [M + H]$^+$: 265.0618; found: 265.0619, (Δ = 0.38 ppm).

IR (ATR)/cm^{-1} 2954, 2929, 2857, 1631, 1464, 1253, 1098, 925, 886, 833, 774.

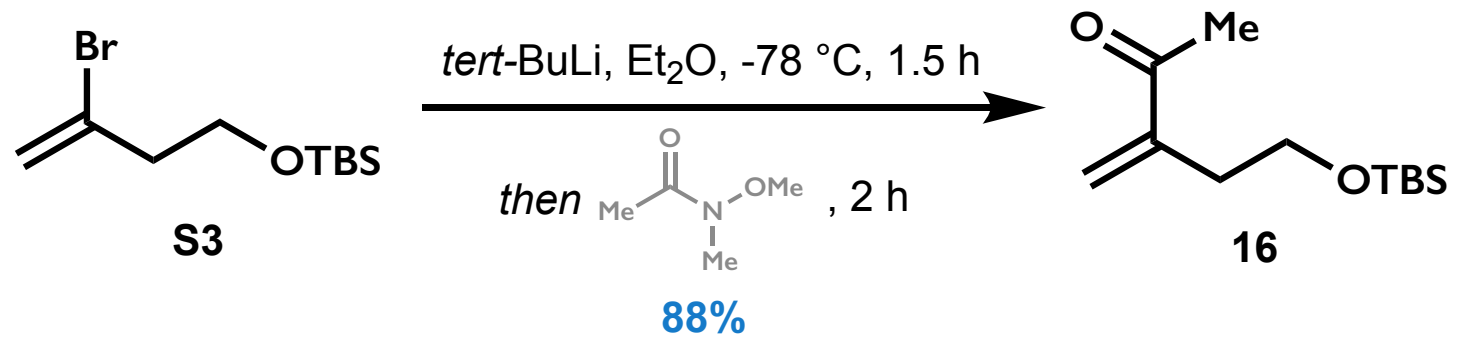

Enone 16. Under an inert atmosphere at -78 °C, *tert*-BuLi (9.42 mL, 15.1 mmol ,1.6 M in pentane) was added dropwise to silyl ether **S3** (2.00 g, 7.54 mmol) in dry Et_2O (10 mL). After 1.5 h, *N*-Methoxy-*N*-methylacetamide (802 µL, 7.54 mmol) was added dropwise, and the mixture was stirred for an additional 2 h. The reaction mixture was then poured into cold sat. aq. NH_4Cl (25 mL). The organic layer was separated, and the aqueous layer was extracted with *n*-pentane (2 x 25 mL). The combined organic extracts were washed with brine (25 mL), dried using a phase separator, and concentrated under reduced pressure. Purification by flash chromatography (silica gel, 3 to 10% EtOAc in PE) afforded α,β-unsaturated **16** (1.52 g, 6.65 mmol, 88%) as a colorless oil.

$\mathbf{R_f}$ 0.42 (silica gel, 10% EtOAc in *n*-heptane).

^{1}H NMR (400 MHz, $CDCl_3$) δ 6.06 (app. s, 1H), 5.87 (app. s, 1H), 3.66 (t, *J* = 6.5 Hz, 2H), 2.48 (t, *J* = 7.0 Hz, 2H), 2.33 (s, 3H), 0.87 (s, 9H), 0.02 (s, 6H).

^{13}C{^{1}H} NMR (101 MHz, $CDCl_3$) δ 199.8, 146.1, 127.1, 62.0, 34.4, 26.1, 25.9, 18.4, -5.2.

HRMS (ESI+) *m/z:* calcd. for $C_{12}H_{25}O_2Si^+$ [M + H]$^+$: 229.1618; found: 229.1620, (Δ = 0.87 ppm).

IR (ATR)/cm^{-1} 2955, 2929, 2857, 1679, 1362, 1254, 1093, 925, 833, 774.

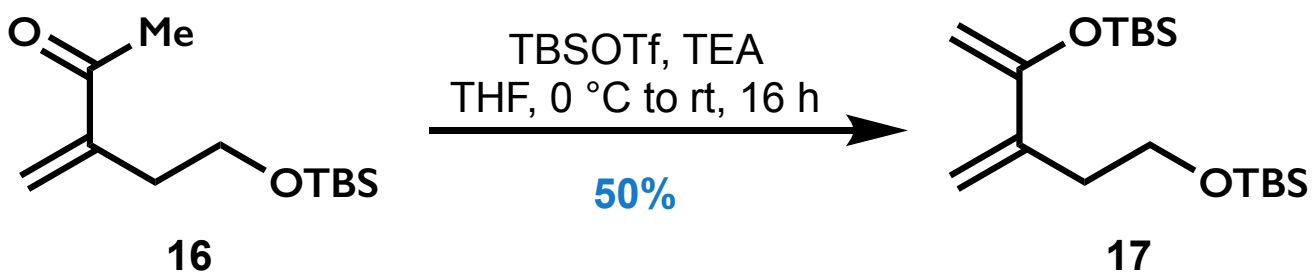

Silyl enol ether 17. Under an inert atmosphere at 0 °C, TBSOTf (3.01 mL, 13.1 mmol) was slowly added dropwise to a solution of enone **16 (**1.50 g, 6.57 mmol) and triethylamine (3.66 mL, 26.3 mmol) in dry THF (30 mL). The colorless solution was stirred for 16 h, slowly reaching rt. The orange solution was then quenched with triethylamine (5 mL), *n*-pentane (20 mL), and sat. aq. $NaHCO_3$ (40 mL). The organic layer was separated, and the aqueous layer was extracted with EtOAc (2 x 25 mL). The combined organic phases were washed with sat. aq. $NaHCO_3$ (30 mL), distilled water (30 mL), and brine (30 mL), dried using a phase separator, and concentrated under reduced pressure. Purification by flash chromatography (basic aluminum oxide, 0 to 5% EtOAc in *n*-heptane) afforded silyl enol ether **17** (1.13 g, 3.30 mmol, 50%) as a colorless oil.

$\mathbf{R_f}$ 0.67 (silica gel, 20% EtOAc in *n*-heptane).

^{1}H NMR (400 MHz, $CDCl_3$) δ 5.51 (d, *J* = 2.2 Hz, 1H), 4.99 (app. s, 1H), 4.52 (app. s, 1H), 4.33 (app. s, 1H), 3.73 (t, *J* = 7.3 Hz, 2H), 2.46 (t, *J* = 7.3 Hz, 2H), 0.96 (s, 9H), 0.89 (s, 9H), 0.17 (s, 6H), 0.05 (s, 6H).

^{13}C{^{1}H} NMR (101 MHz, $CDCl_3$) δ 155.9, 141.2, 114.6, 92.6, 63.0, 36.8, 26.1, 26.0, 18.5, 18.4, -4.5, -5.1.

HRMS (ESI+) *m/z:* calcd. for $C_{18}H_{38}NaO_2Si_2^+$ [M + Na]$^+$: 365.2303; found: 365.2297, (Δ = -1.64 ppm).

IR (ATR)/cm^{-1} 2955, 2930, 2858, 1588, 1472, 1463, 1254, 1097, 1015, 1004, 776.

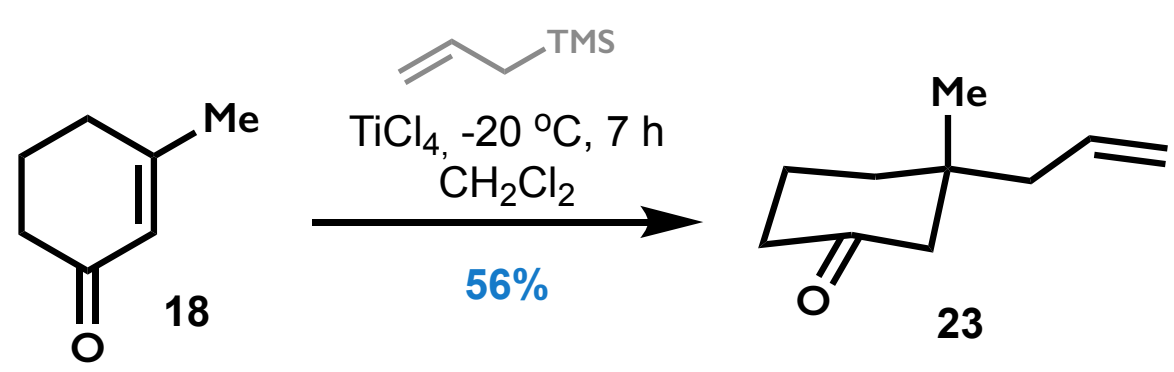

3-allyl-3-methylcyclohexanone (23). Under an inert atmosphere at -20 °C, $TiCl_4$ (14.0 mL, 14.0 mmol, 1 M in CH_2Cl_2) was slowly added dropwise to a solution of 3-methyl-2-cyclohexen-1-one (**18**) (1.59 mL, 14.0 mmol) in dry CH_2Cl_2 (14 mL). After 2 min, allyltrimethylsilane (3.11 mL, 19.6 mmol) was added dropwise to the dark red solution. The solution was stirred for 7 h at -20 °C, quenched by a dropwise addition of a solution of triethylamine (7 mL), MeOH (2.25 mL), and CH_2Cl_2 (20 mL). The resulting mixture was stirred for 5 min and diluted with CH_2Cl_2 (25 ml). The organic layer was separated and washed with 10% HCl (2 x 50 mL), sat. aq. $NaHCO_3$ (50 mL), and brine (50 mL). Drying over $NaSO_4$, concentration under reduced pressure, and purification by flash chromatography (silica gel, 0 to 10% EtOAc in PE) afforded cyclohexanone **23** (1.20 g, 7.88 mmol, 56%) as a faint yellow oil.

R_f 0.50 (silica gel, 10 % EtOAc in *n*-heptane).

^{1}H NMR (400 MHz, $CDCl_3$) δ 5.84 – 5.72 (m, 1H), 5.11 – 5.00 (m, 2H), 2.32 – 2.25 (m, 2H), 2.24 – 2.06 (m, 2H), 2.04 – 2.00 (d, *J* = 7.5, 2H), 1.94 – 1.81 (m, 2H), 1.67 – 1.50 (m, 2H), 0.92 (s, 3H).

^{13}C{^{1}H} NMR (101 MHz, $CDCl_3$) δ 212.4, 133.9, 118.4, 53.4, 46.2, 41.1, 38.9, 35.7, 25.2, 22.1.

HRMS (ESI+) *m/z:* calcd. for $C_{10}H_{17}O^+$ [M + H]$^+$: 153.1274; found: 153.1268, (Δ = -3.92 ppm).

IR (ATR)/cm^{-1} 2957, 2937, 2876, 2854, 1709, 1456, 1426, 1380, 1312, 1226, 998, 913, 505.

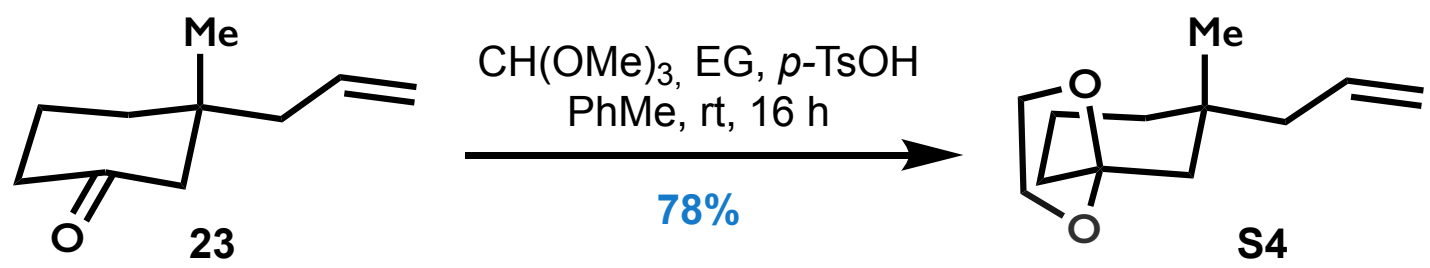

Acetal S4. A solution of cyclohexanone **23** (3.18 g, 20.9 mmol), ethylene glycol (EG, 7.01 mL, 125 mmol), trimethyl orthoformate (5.72 mL, 52.3 mmol), and *p*-TsOH monohydrate (398 mg, 2.09 mmol) in dry toluene (2.6 mL) was stirred under an inert atmosphere at rt for 16 h. The faint yellow solution was quenched by the addition of sat. aq. $NaHCO_3$ (6 mL) and diluted with EtOAc (10 mL). The organic layer was separated, and the aqueous phase was extracted with EtOAc (3 x 10 mL). The combined organic extracts were washed with brine (20 mL) and dried using a phase separator. Concentration under reduced pressure and purification by flash chromatography (silica gel, 0 to 5% EtOAc in heptane) afforded acetal **S4** (3.20 g, 16.3 mmol, 78%) as a colorless oil.

R_f 0.65 (silica gel, 20 % EtOAc in *n*-heptane).

^{1}H NMR (400 MHz, $CDCl_3$) δ 5.81 (ddt, *J* = 16.8, 10.3, 7.5 Hz, 1H), 5.05 – 4.96 (m, 2H), 3.94 – 3.88 (app. s, 4H), 2.12 – 1.99 (m, 2H), 1.66 – 1.36 (m, 6H),1.34 – 1.20 (m, 2H), 0.95 (s, 3H).

^{13}C{^{1}H} NMR (101 MHz, $CDCl_3$) δ 135.5, 117.2, 109.6, 64.2, 64.1, 46.8, 44.7, 37.0, 35.2, 35.0, 26.1, 19.8.

HRMS (ESI+) *m/z:* calcd. for $C_{12}H_{20}NaO_2^+$ [M + Na]$^+$: 219.1356; found: 219.1364, (Δ = 3.65 ppm).

IR (ATR)/cm^{-1} 2935, 2873, 1456, 1360, 1178, 1051, 911, 825, 513.

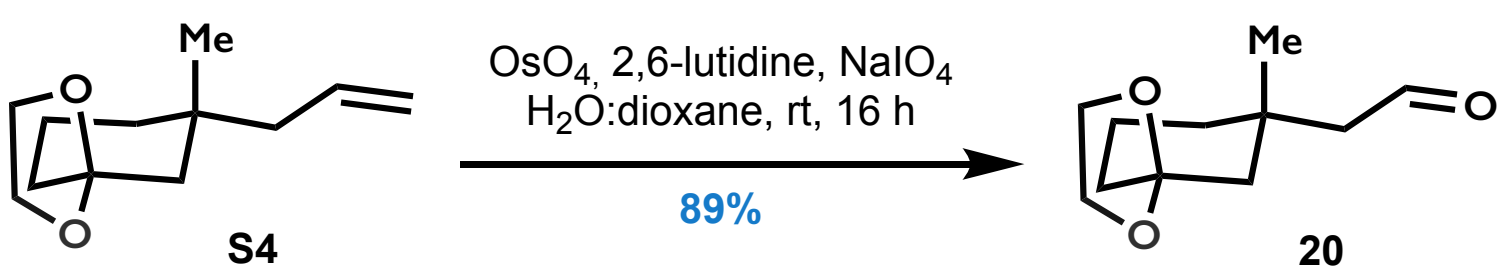

Aldehyde 20. 2,6-lutidine (3.92 mL, 33.6 mmol), OsO_4 (551 µL, 42.0 µmol, 2.5 wt.% in *tert*-BuOH), and $NaIO_4$ (7.19 g, 33.6 mmol) were added sequentially added to acetal **S4** (1.65 g, 8.41 mmol) in 1,4-dioxane/water (70 mL, 3:1) at rt under air. The reaction mixture was stirred for 16 h, resulting in a white slurry. The mixture was diluted with distilled water (5 mL) and CH_2Cl_2 (5 mL). The organic phase was separated, and the aqueous phase was extracted with CH_2Cl_2 (3 x 10 mL). The combined organic extracts were washed with distilled water (30 mL), HCl (30 mL, 1 M), sat. aq. $NaHCO_3$ (30 mL), and brine (30 mL), dried using a

phase separator, and concentrated under reduced pressure. Purification by flash chromatography (neutral aluminum oxide, 10% EtOAc in PE) afforded aldehyde **20** (1.90 g, 7.48 mmol, 89%) as a faint yellow oil.

R*f* 0.27 (silica gel, 20 % EtOAc in *n*-heptane).

^{1}H NMR (400 MHz, $CDCl_3$) δ 9.85 (t, *J* = 2.9 Hz, 1H), 3.91 (app. s, 4H), 2.63 (dd, J = 15.0, 2.6 Hz, 1H), 2.31 (dd, J = 15.0, 3.1 Hz, 1H), 1.81 – 1.61 (m, 4H), 1.56 – 1.45 (m, 3H), 1.33 – 1.25 (m, 1H), 1.12 (s, 3H).

^{13}C{^{1}H} NMR (126 MHz, $CDCl_3$) δ 204.0, 109.0, 64.4, 64.2, 53.2, 45.0, 37.9, 35.0, 34.8, 28.1, 19.8.

HRMS (ESI+) *m/z:* calcd. for $C_{11}H_{19}O_3^+$ [M + H]$^+$: 199.1329; found: 199.1333, (Δ = 2.01 ppm).

IR (ATR)/cm^{-1} 2936, 2875, 1717, 1456, 1361, 1178, 1089, 1042, 936, 855, 830, 687, 514.

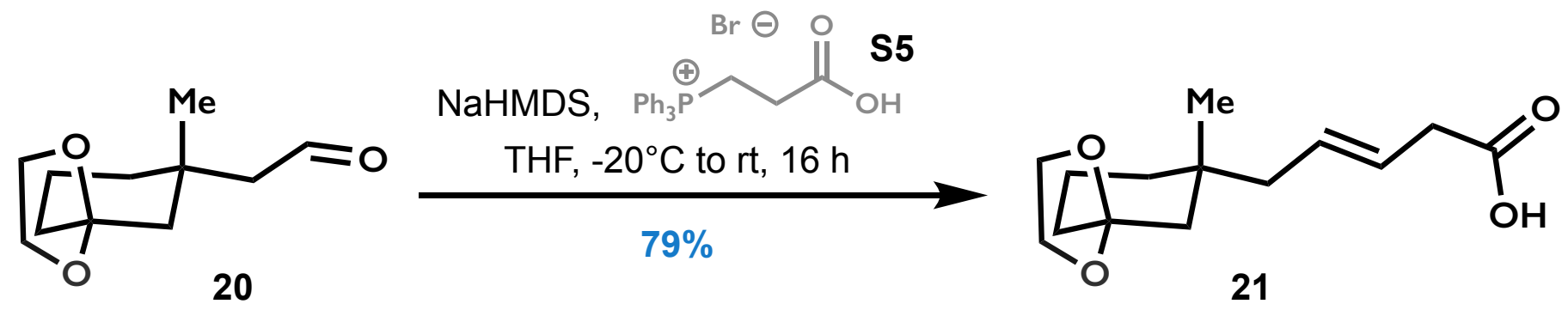

Pentenoic acid 21. Under an inert atmosphere at -20 °C, NaHMDS (13.9 mL, 13.9 mmol, 1M in THF) was added dropwise to (2-carboxyethyl)triphenylphosphonium bromide (**S5**, 2.62 g, 6.31 mmol) in dry THF (10 mL). After 30 min, a solution of aldehyde **20** (1.00 g, 5.08 mmol) in dry THF (3 mL) was added dropwise. The reaction mixture was stirred for 16 h, slowly reaching rt, after which the reaction was quenched by addition of sat. aq. NH_4Cl (20 mL). The organic layer was separated, and the aqueous phase was acidified with HCl (30 mL, 1M) and extracted with 2-MeTHF (3 x 30 mL). The combined organic phases were washed with brine (40 mL), dried using a phase separator, and concentrated under reduced pressure. Purification by flash chromatography (silica gel, 5 to 25% EtOAc in PE) afforded pentenoic acid **21** (1.02 g, 4.01 mmol, 79%) as a colorless oil, isolated as a mixture of stereoisomers.

R*f* 0.30 (silica gel, 90 % EtOAc in *n*-heptane).

^{1}H NMR (400 MHz, $CDCl_3$) δ 5.72 – 5.48 (m, 2H), 3.97 – 3.87 (app. s, 4H), 3.20 – 3.07 (m, 2H), 2.17 – 1.97 (m, 2H), 1.65 – 1.22 (m, 8H), 0.95 (app. d, *J* = 8.3 Hz, 3H) (Summary of stereoisomers, dr 1.1:1).

^{13}C{^{1}H} NMR (126 MHz, $CDCl_3$) δ 176.5, 176.5, 132.0, 130.4, 123.6, 122.0, 109.6, 109.5, 64.2, 64.2, 64.1 (two overlapping signals), 44.7, 44.7, 37.8, 37.7, 37.1, 37.0, 35.5, 35.2, 35.1, 35.1, 32.6 (two overlapping signals), 26.4, 26.2, 19.9, 19.8 (Summary of diastereoisomers).

HRMS (ESI+) *m/z:* calcd. for $C_{14}H_{22}NaO_4^+$ [M + Na]$^+$: 277.1410; found: 277.1408, (Δ = -0.72 ppm).

IR (ATR)/cm^{-1} 2931, 2874, 2850, 1707, 1360, 1222, 1176, 1091, 1050, 935.

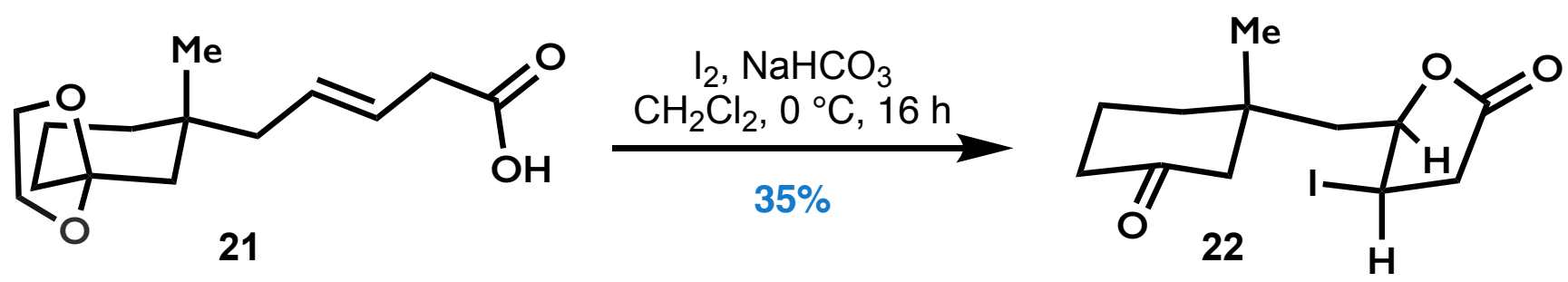

Iodolactone 22. Under an inert atmosphere at 0 °C, I_2 (898 mg, 3.54 mmol) and $NaHCO_3$ (297 mg, 3.54 mmol) were added to a solution of pentenoic acid **21** (300 mg, 1.18 mmol) in dry CH_2Cl_2 (6 mL). The reaction mixture was stirred for 16 h while slowly reaching rt. The mixture was quenched by addition of sat. aq. $NaHCO_3$ (4 mL). The organic layer was separated, and the aqueous phase was extracted with EtOAc (3 x 10 mL). The combined organic extracts were washed with 10% aq. $Na_2S_2O_3$ (10 mL), dried using a phase separator, and concentrated under reduced pressure. Purification by flash chromatography (silica gel, 0 to 50% EtOAc in PE) afforded iodolactone **22** (139 mg, 0.414 mmol, 35%) as a faint yellow oil, isolated as a mixture of diastereoisomers.

R*f* 0.57 (silica gel, 40 % EtOAc in *n*-heptane).

^{1}**H NMR** (400 MHz, $CDCl_3$) δ 4.74 – 4.65 (m, 1H), 3.96 – 3.87 (dtd, *J* = 10.8, 8.3, 2.3 Hz, 1H), 3.10 (dd, *J* = 17.8, 8.0 Hz, 1H), 2.88 (dd, *J* = 17.9, 10.4 Hz, 1H), 2.36 – 2.16 (m, 4H), 1.98 – 1.58 (m, 6H), 1.06 – 1.01 (app. d, *J* = 2.7, 3H) (Summary of diastereoisomers, dr 1.4:1).

^{13}**C{^{1}H} NMR** (101 MHz, $CDCl_3$) δ 211.1, 211.0, 173.7, 173.6, 85.5, 85.4, 54.0, 53.8, 44.4, 44.1, 41.0, 40.9, 40.5 (two overlapping signals), 38.1, 38.0, 35.7, 35.7, 25.3, 25.2, 22.1, 22.0, 16.0, 15.9 (Summary of diastereoisomers).

HRMS (ESI+) *m/z:* calcd. for $C_{12}H_{18}IO_3^+$ [M + H]$^+$: 337.0295; found: 337.0293, (Δ = -0.59 ppm).

IR (ATR)/cm^{-1} 2954, 2934, 1781, 1705, 1457, 1421, 1352, 1198, 1145, 962.

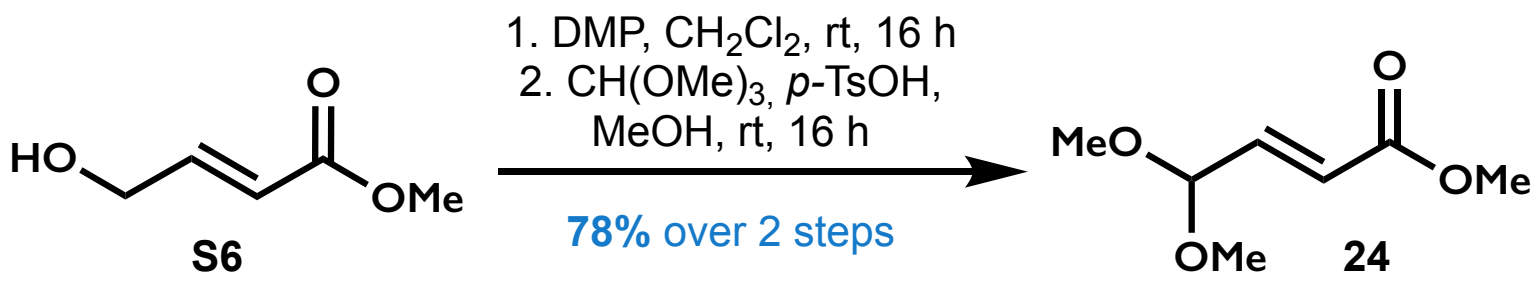

Butenoate 24. Dess-Martin periodinane (DMP, 11.1 g, 26.1 mmol) was added in one portion to a solution of acrylate **S6** (2.98 g, 26.1 mmol, prepared according to the procedure of Hébert *et al.*[23]) in CH_2Cl_2 (230 mL) at rt under air. The reaction mixture was stirred for 16 h and quenched by addition of sat. aq. $NaHCO_3$ (50 mL). The organic layer was separated, and the aqueous phase was extracted with EtOAc (2 x 50 mL). The combined organic extracts were washed with brine (100 mL), dried over $MgSO_4$, and concentrated under reduced pressure to afford a faint yellow solid. The crude aldehyde was used in the next step without further purification.

Under an inert atmosphere at rt, trimethyl orthoformate (9.14 mL, 83.6 mmol), and *p*-TsOH monohydrate (530 mg, 2.77 mmol) was added to a solution of the crude aldehyde in dry MeOH (16 mL). The reaction mixture was stirred for 16 h, quenched by the addition of sat. aq. $NaHCO_3$ (10 mL), and diluted with EtOAc (10 mL). The organic layer was separated, and the aqueous layer was extracted with EtOAc (3 x 10 mL). The combined organic extracts were washed with brine (20 mL), dried over Na_2SO_4, and concentrated under reduced pressure. Purification by flash chromatography (silica gel, 0 to 20% EtOAc in PE) afforded acetal **24** (3.28 g, 20.5 mmol, 74%) as a colorless oil.

R_f 0.43 (silica gel, 20% EtOAc in PE).

^{1}**H NMR** (400 MHz, $CDCl_3$) δ 6.77 (dd, *J* = 15.9, 3.9 Hz, 1H), 6.14 (dd, *J* = 15.9, 1.5 Hz, 1H), 4.94 (dd, *J* = 4.0, 1.4 Hz, 1H), 3.75 (s, 3H), 3.33 (s, 6H).

^{13}**C{^{1}H} NMR** (101 MHz, $CDCl_3$) δ 166.5, 143.0, 124.4, 100.6, 52.9, 51.9.

HRMS (ESI+) *m/z:* calcd. for $C_7H_{12}NaO_4^+$ [M + Na]$^+$: 183.0628; found: 183.0631, (Δ = 1.64 ppm).

IR (ATR)/cm^{-1} 2995, 2953, 2834, 1727, 1437, 1305, 1274, 1192, 1169, 1129, 1055.

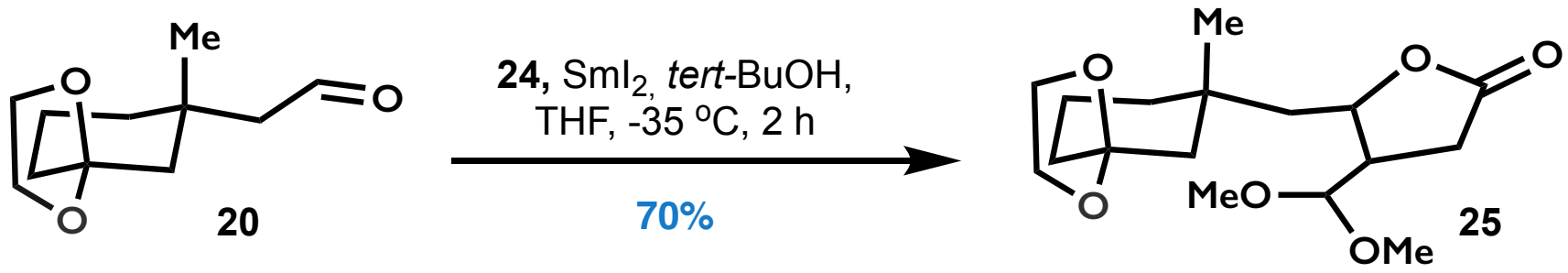

Lactone 25. In a glovebox under nitrogen atmosphere at -35 °C, aldehyde **20** (327 mg, 1.65 mmol), acrylate **24** (240 mg, 1.50 mmol), and *tert*-BuOH (667 mg, 9.00 mmol) in dry THF (7.5 mL) were added to freshly prepared SmI_2 (30.0 mL, 0.10 M in THF, prepared according to the procedure of Janjetovic *et al.*[24]). The deep blue solution was placed in the freezer at -35 °C for 2 h. The flask was then sealed, removed from the glovebox, and quenched with brine (30 mL) at -40 °C (MeCN, dry ice). The organic layer was separated, and the aqueous layer was extracted with EtOAc (3 x 30 mL). The combined organic extracts were dried over $MgSO_4$, concentrated under reduced pressure and purified by flash chromatography (basic aluminum

oxide, 0 to 10% EtOAc in PE), affording lactone **25** (342 mg, 1.05 mmol, 70%) as a faint yellow oil and as a mixture of more than two inseparable stereoisomers.

R_f 0.57 (silica gel, 60% EtOAc in PE).

^{1}H NMR (400 MHz, $CDCl_3$) δ 4.71 – 4.65 (m, 1H), 4.43 – 4.39 (m, 1H), 3.96 – 3.83 (m, 4H), 3.41 – 3.32 (m, 6H), 2.87 – 2.71 (m, 1H), 2.59 – 2.41 (m, 2H), 1.91 – 1.40 (m, 9H), 1.34 – 1.23 (m, 1H), 1.20 (app. d, J = 3.0 Hz, 3H) (Summary of diastereomers).

^{13}C{^{1}H} NMR (101 MHz, $CDCl_3$) δ 176.5, 176.3, 109.6, 109.4, 109.3, 103.6, 103.4, 78.6, 78.5, 64.3, 64.3, 64.2, 64.1, 64.1, 54.5, 54.3, 54.1, 53.4, 45.6, 45.3, 42.2, 42.1, 39.0, 38.1, 38.6, 37.7, 35.2, 34.9, 34.7, 34.4, 30.5, 30.5, 27.2 (two overlapping signals), 19.8, 19.7 (Summary of diastereomers).

HRMS (ESI+) *m/z:* calcd. for $C_{17}H_{28}NaO_6^+$ [M + Na]$^+$: 351.1778; found: 351.1777, (Δ = -0.28 ppm).

IR (ATR)/cm^{-1} 2933, 2890, 2849, 1774, 1456, 1360, 1176, 1131, 1089, 1063.

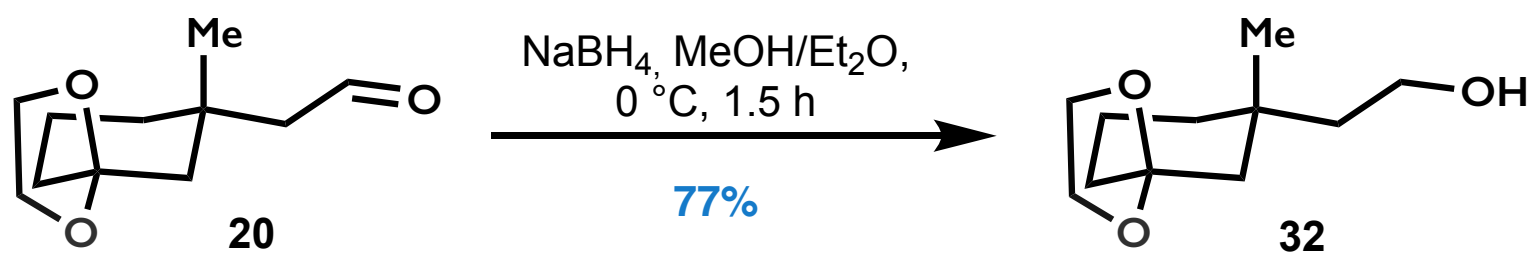

Alcohol 32. $NaBH_4$ (57.2 mg, 1.51 mmol) was added in one portion to aldehyde **20** (150 mg, 0.757 mmol) in dry MeOH/Et_2O (8 mL, 1:1) at 0 °C under inert atmosphere. The colorless solution was allowed to warm to rt and stirred for 1.5 h. The solution was then quenched with sat. aq. $NaHCO_3$ (4 mL) and diluted with EtOAc (10 mL). The organic layer was separated, and the aqueous phase was extracted with EtOAc (3 x 20 mL). The combined organic phases were washed with sat. aq. NH_4Cl (20 mL) and brine (20 mL), dried using a phase separator, and concentrated under reduced pressure. Flash chromatography (silica gel, 10 to 50% EtOAc in heptane) afforded alcohol **32** (117 mg, 0.585 mmol, 77%) as a colorless oil.

R_f 0.50 (silica gel, 80 % EtOAc/*n*-heptane).

^{1}H NMR (400 MHz, $CDCl_3$) δ 3.96 – 3.88 (m, 4H), 3.75 – 3.68 (m, 2H), 1.86 – 1.20 (m, 10H), 0.99 (s, 3H).

^{13}C{^{1}H} NMR (101 MHz, $CDCl_3$) δ 109.6, 64.3, 64.1, 59.6, 44.9, 43.5, 38.1, 34.7, 34.2, 27.6, 19.7.

HRMS (ESI+) *m/z:* calcd. for $C_{11}H_{20}NaO_3^+$ [M + Na]$^+$: 223.1305; found: 223.1300, (Δ = -2.24 ppm).

IR (ATR)/cm^{-1} 3394, 2930, 2873, 1457, 1360, 1179, 1095, 1051, 1026, 948.

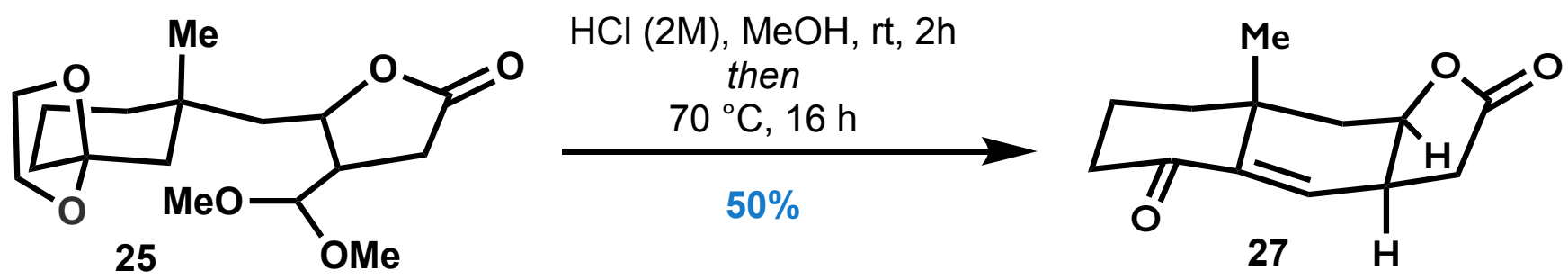

Lactone 27. To a solution of **25** (48.1 mg, 0.147 mmol) in MeOH (1.5 mL) was added HCl (0.88 mL, 2 M) in one portion at rt. The reaction was stirred at rt for 2 h, then heated to 70 °C and stirred for an additional 16 h. The reaction mixture was quenched by addition of sat. aq. $NaHCO_3$ until pH 7 and diluted with EtOAc (3 mL). The organic layer was separated, and the aqueous phase was extracted with EtOAc (3 x 3 mL). The combined organic extracts were dried using a phase separator and concentrated under reduced pressure. Purification by flash chromatography (silica gel, 20 to 60% EtOAc in PE) afforded **27** (16.1 mg, 0.0731 mmol, 50%) as white crystals.[1,2]

R_f 0.23 (silica gel, 60% EtOAc in PE).

---

[1] The isomerization affords 27 as a single product (confirmed by crude NMR).

[2] Decomposition is observed during column chromatography purification.

^{1}H NMR (500 MHz, $CDCl_3$) δ 6.14 (dd, *J* = 3.6, 0.9 Hz, 1H), 4.85 (dtd, *J* = 6.1, 3.1, 0.9 Hz, 1H), 3.22 (m, 1H), 2.94 (dd, *J* = 17.7, 10.0 Hz, 1H), 2.61 (ddt, *J* = 16.9, 5.1, 2.0 Hz, 1H), 2.49 (app. d, J = 17.7, 1H), 2.38 – 2.26 (m, 2H), 2.09 – 1.98 (m, 1H), 1.93 – 1.85 (m, 1H), 1.78 (dtd, *J* = 13.3, 3.5, 2.0 Hz, 1H), 1.67 (dd, *J* = 15.1, 3.2 Hz, 1H), 1.62 – 1.54 (m, 1H), 1.18 (s, 3H).

^{13}C{^{1}H} NMR (126 MHz, $CDCl_3$) δ 201.8, 175.5, 145.2, 129.2, 77.5, 40.5, 39.6, 39.3, 35.5, 34.8, 34.6, 26.8, 18.8.

HRMS (ESI+) *m/z:* calcd. for $C_{13}H_{17}O_3^+$ [M + H]$^+$: 221.1172; found: 221.1169, (Δ = -1.36 ppm).

IR (ATR)/cm^{-1} 2925, 2878, 2854, 1768, 1688, 1628, 1419, 1329, 1292, 1264, 1230, 1182, 1158, 1019, 922.

mp 125 – 127 °C.

When **25** is subjected to less acidic ring closing conditions (i.e. *p*-TsOH), a mixture of two diastereoisomers **27** and **28** is obtained:

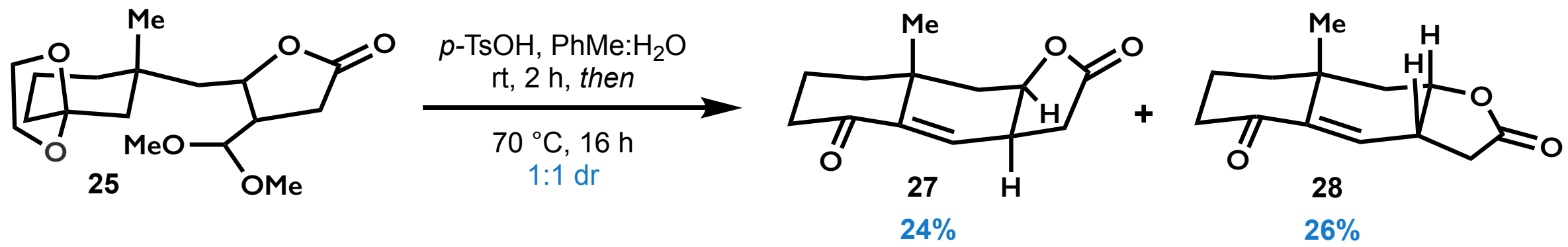

Lactone 27 and **28.** To a solution of **25** (280.8 mg, 0.855 mmol) in PhMe/H_2O (8.6 mL, 5:1) was added *p*-TsOH monohydrate (325 mg, 1.71 mmol) in one portion at rt. The reaction was stirred at rt for 2 h, then heated to 70 °C and stirred for an additional 16 h. The reaction mixture was quenched by addition of sat. aq. $NaHCO_3$ until pH 7 and diluted with EtOAc (3 mL). The organic layer was separated, and the aqueous phase was extracted with EtOAc (3 x 3 mL). The combined organic extracts were dried using a phase separator and concentrated under reduced pressure. Purification by flash chromatography (silica gel, 20 to 60% EtOAc in PE) afforded **27** (45.2 mg, 0.205 mmol, 24%) and **28** (48.8, 0.222 mmol, 26%) as separable white crystals.[2,3]

28:

R_f 0.26 (silica gel, 40% EtOAc in PE).

^{1}H NMR (400 MHz, $CDCl_3$) δ 6.46 (d, *J* = 4.0 Hz, 1H), 4.90 (ddd, *J* = 12.1, 8.0, 5.3 Hz, 1H), 3.27 (m, 1H), 2.81 (dd, *J* = 17.7, 9.7 Hz, 1H), 2.61 (ddt, *J* = 17.1, 4.6, 2.2 Hz, 1H), 2.38 – 2.26 (m, 2H), 2.11 (dd, *J* = 12.7, 5.3 Hz, 1H), 2.06 – 1.87 (m, 2H), 1.77 (dtd, *J* = 13.5, 3.5, 2.0 Hz, 1H), 1.63 (td, *J* = 13.3, 4.1 Hz, 1H), 1.49 (t, *J* = 12.4 Hz, 1H), 1.11 (s, 3H).

^{13}C{^{1}H} NMR (126 MHz, $CDCl_3$) δ 200.7, 175.2, 144.2, 130.8, 75.6, 41.2, 40.2, 38.0, 37.2, 34.9, 33.5, 25.1, 18.6.

HRMS (ESI+) *m/z:* calcd. for $C_{13}H_{17}O_3^+$ [M + H]$^+$: 221.1172; found: 221.1169, (Δ = -1.36 ppm).

IR (ATR)/cm^{-1} 2950, 2924, 2867, 1765, 1680, 1622, 1456, 1421, 1335, 1318, 1302, 1261, 1245, 1173, 1136, 1006.

mp 120 – 124 °C.

[3] The isomerization affords 27 and 28 as single products (confirmed by NMR).

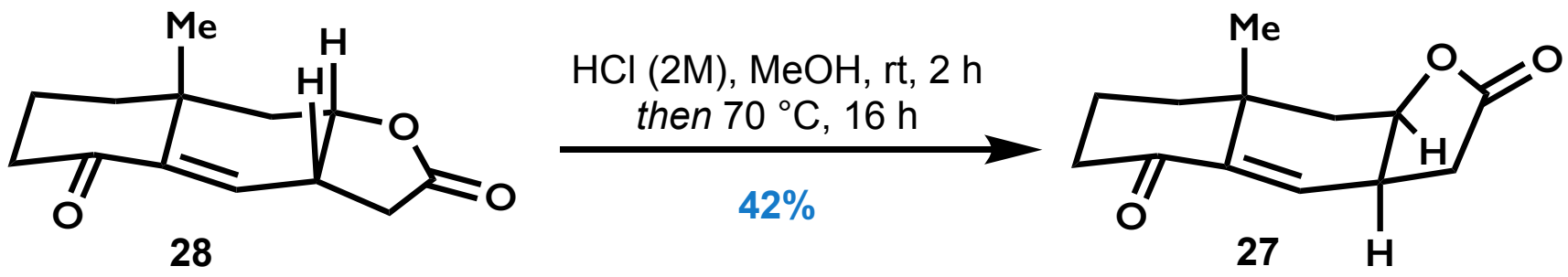

Lactone 27. To a solution of 28 (74.5 mg, 0.338 mmol) in MeOH (3.4 mL) was added HCl (2.03 mL, 2 M) in one portion at rt. The reaction was stirred at rt for 2 h, then heated to 70 °C and stirred for an additional 16 h. The reaction mixture was quenched by addition of sat. aq. $NaHCO_3$ until pH 7 and diluted with EtOAc (3 mL). The organic layer was separated, and the aqueous phase was extracted with EtOAc (3 x 3 mL). The combined organic extracts were dried using a phase separator and concentrated under reduced pressure. Purification by flash chromatography (silica gel, 20 to 60% EtOAc in PE) afforded 27 (31.3 mg, 0.142 mmol, 42%) as white crystals.[1,2]

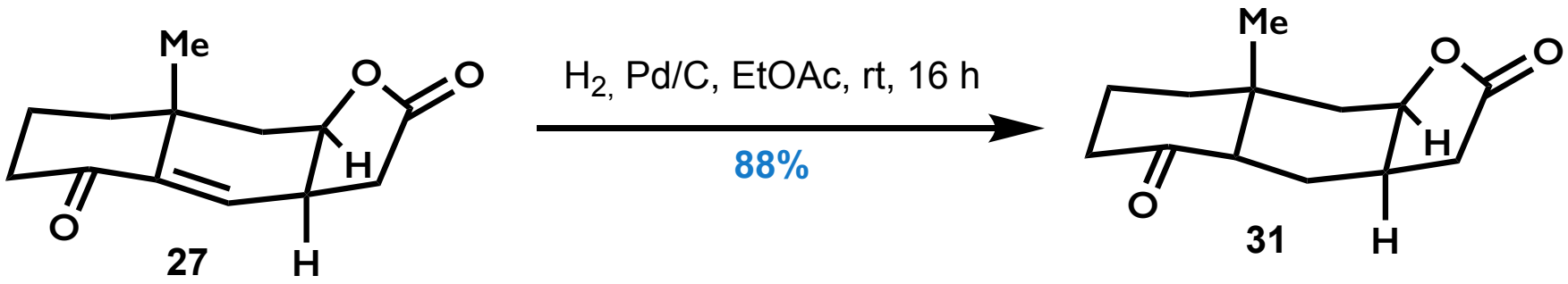

Lactone 31. Under an inert atmosphere at rt, palladium on carbon (25.3 mg, 23.8 µmol, 10 wt%) was added in one portion to a solution of lactone **27** (105 mg, 0.477 mmol) in dry EtOAc (5 mL). The reaction mixture was degassed with H_2 for 5 min and left stirred under an atmosphere of hydrogen (1 atm) for 16 h. The reaction mixture was filtered through a plug of silica gel topped with celite and concentrated under reduced pressure. Purification by flash chromatography (silica gel, 20% EtOAc in PE) afforded **31** (93.0 mg, 0.42 mmol, 88%) as white crystals.

$\mathbf{R}_f$ 0.23 (silica gel, 60% EtOAc in PE).

^{1}H NMR (400 MHz, $CDCl_3$) δ 4.54 (app. td, *J* = 4.5, 1.8 Hz, 1H), 2.72 (dd, *J* = 17.0, 7.0 Hz, 1H), 2.42 – 2.18 (m, 6H), 1.97 – 1.79 (m, 3H), 1.73 – 1.57 (m, 3H), 1.32 (app. dt, *J* = 14.7, 12.5 Hz, 1H), 0.88 (s, 3H).

^{13}C{^{1}H} NMR (126 MHz, $CDCl_3$) δ 211.1, 176.7, 78.8, 54.4, 41.3, 41.3, 40.7, 38.0, 37.9, 35.1, 22.4, 21.9, 18.7.

HRMS (ESI+) *m/z:* calcd. for $C_{13}H_{19}O_3^+$ [M + H]$^+$: 223.1329; found: 223.1323, (Δ = -2.69 ppm).

IR (ATR)/cm^{-1} 2925, 2876, 2854, 1766, 1705, 1422, 1306, 1271, 1212, 1194, 1155, 1075, 991, 924.

mp 145 – 149 °C.

Table S2. Comparison of the ^{1}H NMR, ^{13}C NMR, and melting point (MP) data for **31** with values reported by Tada *et al.*[4]

**Analysis**	**This work** (^{1}H NMR (400 MHz, $CDCl_3$); ^{13}C{^{1}H} NMR (126 MHz, $CDCl_3$))	**Tada *et al.***[4] (MHz not specified)
**^{1}H NMR**	4.54 (app. td, *J* = 4.5, 1.8 Hz, 1H)	4.56 (ddd, *J* = 5.0, 5.0, 2.0 Hz, 1H)
	2.72 (dd, *J* = 17.0, 7.0 Hz, 1H)	2.73 (dd, *J* = 16.0, 7.0 Hz, 1H)
	2.42 – 2.18 (m, 6H)	2.44 – 2.18 (m, 6H)
	1.97 – 1.79 (m, 3H)	1.98 – 1.79 (m, 3H)
	1.73 – 1.57 (m, 3H)	1.75 – 1.55 (m, 3H)
	1.32 (app. dt, *J* = 14.7, 12.5 Hz, 1H)	1.33 (ddd, *J* = 15.0, 12.0, 12.0 Hz, 1H)
	0.88 (s, 3H)	0.9 (s, 3H)
	211.1	210.7
	176.7	176.4
	78.8	78.8
	54.4	54.4
	41.3	41.3

**^{13}C NMR**	41.3	41.1
	40.7	40.6
	38.0	37.9
	37.9	37.8
	35.1	35.1
	22.4	22.3
	21.9	21.7
	18.7	18.6
**MP**	145 – 149 °C	153 – 155 °C

IV. Supplementary References

(1) Krzyzanowski, A.; Pahl, A.; Grigalunas, M.; Waldmann, H. Spacial Score–A Comprehensive Topological Indicator for Small-Molecule Complexity. *J. Med. Chem.* **2023**, *66* (18), 12739–12750. https://doi.org/10.1021/acs.jmedchem.3c00689.

(2) Amber, C.; Nakamura, T.; Amoako, M.; Settineri, N. S.; Sarpong, R. An “Inside-Out” Strategy Enables a 14-Step Total Synthesis of Hispidospermidin. *JACS Au* **2026**, *6* (1), 600–606. https://doi.org/10.1021/jacsau.5c01567.

(3) Johnson, J. S. Counting Steps. *Nat. Synth.* **2023**, *2* (1), 6–8. https://doi.org/10.1038/s44160-022-00204-3.

(4) Tada, M.; Yamada, H.; Kanamori, A.; Chiba, K. Total Synthesis of Three Eudesman-12,8-Olides, (±)-Isoalantolactone, (±)-Dihydrocallitrisin and (±)-Septuplinolide; Structure Revision of Septuplinolide. *J Chem Soc Perkin Trans 1* **1993**, No. 2, 239–247. https://doi.org/10.1039/P19930000239.

(5) Minato, H.; Horibe, I. Studies on Sesquiterpenoids. Part XVI. A New Synthetic Method for α-Methylene-γ-Lactones; Total Synthesis of (±)-Isoalantolactone. *J Chem Soc C* **1967**, *0* (0), 1575–1577. https://doi.org/10.1039/J39670001575.

(6) Blay, G.; Cardona, L.; García, B.; Pedro, J. R.; Serrano, A. Synthesis of (+)-Isoalantolactone and (+)-Isoalloalantolactone from (−)-Santonin. *Tetrahedron* **1992**, *48* (25), 5265–5272. https://doi.org/10.1016/S0040-4020(01)89024-4.

(7) Blay, G.; Luz Cardona, M.; Garcia, B.; Pedro, J. R. Functionality Transfer from C6 to C8 in Sesquiterpenes. Synthesis of 8-Epi-Ivangustin and 8-Epi-Isoivangustin from Santonin. *J. Org. Chem.* **1991**, *56* (21), 6172–6175. https://doi.org/10.1021/jo00021a040.

(8) Neese, F. Software Update: The ORCA Program System—Version 6.0. *WIREs Comput. Mol. Sci.* **2025**, *15* (2), e70019. https://doi.org/10.1002/wcms.70019.

(9) Halgren, T. A. Merck Molecular Force Field. I. Basis, Form, Scope, Parameterization, and Performance of MMFF94. *J. Comput. Chem.* **1996**, *17* (5–6), 490–519. https://doi.org/10.1002/(SICI)1096-987X(199604)17:5/6%3C490::AID-JCC1%3E3.0.CO;2-P.

(10) De Souza, B. GOAT: A Global Optimization Algorithm for Molecules and Atomic Clusters. *Angew. Chem. Int. Ed.* **2025**, *64* (18), e202500393. https://doi.org/10.1002/anie.202500393.

(11) Bannwarth, C.; Ehlert, S.; Grimme, S. GFN2-xTB—An Accurate and Broadly Parametrized Self-Consistent Tight-Binding Quantum Chemical Method with Multipole Electrostatics and Density-Dependent Dispersion Contributions. *J. Chem. Theory Comput.* **2019**, *15* (3), 1652–1671. https://doi.org/10.1021/acs.jctc.8b01176.

(12) Zhao, Y.; Truhlar, D. G. The M06 Suite of Density Functionals for Main Group Thermochemistry, Thermochemical Kinetics, Noncovalent Interactions, Excited States, and Transition Elements: Two New Functionals and Systematic Testing of Four M06-Class Functionals and 12 Other Functionals. *Theor. Chem. Acc.* **2008**, *120* (1–3), 215–241. https://doi.org/10.1007/s00214-007-0310-x.

(13) Rayne, S.; Forest, K. Performance of the M062X Density Functional against the ISOL Set of Benchmark Isomerization Energies for Large Organic Molecules. *Nat. Preced.* **2010**. https://doi.org/10.1038/npre.2010.5183.1.

(14) Rayne, S.; Forest, K. Gas Phase Isomerization Enthalpies of Organic Compounds: A Semiempirical, Density Functional Theory, and Ab Initio Post-Hartree–Fock Theoretical Study. *J. Mol. Struct. THEOCHEM* **2010**, *948* (1–3), 102–107. https://doi.org/10.1016/j.theochem.2010.02.030.

(15) Weigend, F.; Ahlrichs, R. Balanced Basis Sets of Split Valence, Triple Zeta Valence and Quadruple Zeta Valence Quality for H to Rn: Design and Assessment of Accuracy. *Phys. Chem. Chem. Phys.* **2005**, *7* (18), 3297. https://doi.org/10.1039/b508541a.

(16) Weigend, F. Accurate Coulomb-Fitting Basis Sets for H to Rn. *Phys. Chem. Chem. Phys.* **2006**, *8* (9), 1057. https://doi.org/10.1039/b515623h.

(17) Marenich, A. V.; Cramer, C. J.; Truhlar, D. G. Universal Solvation Model Based on Solute Electron Density and on a Continuum Model of the Solvent Defined by the Bulk Dielectric Constant and Atomic Surface Tensions. *J. Phys. Chem. B* **2009**, *113* (18), 6378–6396. https://doi.org/10.1021/jp810292n.

(18) Wheeler, S. E.; Houk, K. N. Integration Grid Errors for Meta-GGA-Predicted Reaction Energies: Origin of Grid Errors for the M06 Suite of Functionals. *J. Chem. Theory Comput.* **2010**, *6* (2), 395–404. https://doi.org/10.1021/ct900639j.

(19) Legault, C. CYLview, 1.0 b. **2009**.

(20) Nicolaou, K. C.; Yu, R.; Lu, Z.; Alvarez, F. G. Total Synthesis of Gukulenin B via Sequential Tropolone Functionalizations. *J. Am. Chem. Soc.* **2022**, *144* (11), 5190–5196. https://doi.org/10.1021/jacs.2c01305.

(21) DUMMY ARTICLE.

(22) Rosa, D.; Orellana, A. Palladium-Catalyzed Cross-Coupling of Cyclopropanols with Aryl Halides Under Mild Conditions. *Org. Lett.* **2011**, *13* (1), 110–113. https://doi.org/10.1021/ol1026409.

(23) Hébert, M.; Bellavance, G.; Barriault, L. Total Synthesis of Ginkgolide C and Formal Syntheses of Ginkgolides A and B. *J. Am. Chem. Soc.* **2022**, *144* (39), 17792–17796. https://doi.org/10.1021/jacs.2c08351.

(24) Janjetovic, M.; Träff, A. M.; Ankner, T.; Wettergren, J.; Hilmersson, G. Solvent Dependent Reductive Defluorination of Aliphatic C–F Bonds Employing Sm(HMDS)2. *Chem. Commun.* **2013**, *49* (18), 1826. https://doi.org/10.1039/c3cc37828d.

V. Copies of ^{1}H, ^{13}C and 2D NMR of Compounds **S3**-**S4**, **16**-**17**, **20**-**25**, **27**-**28**, and **31**-**32**

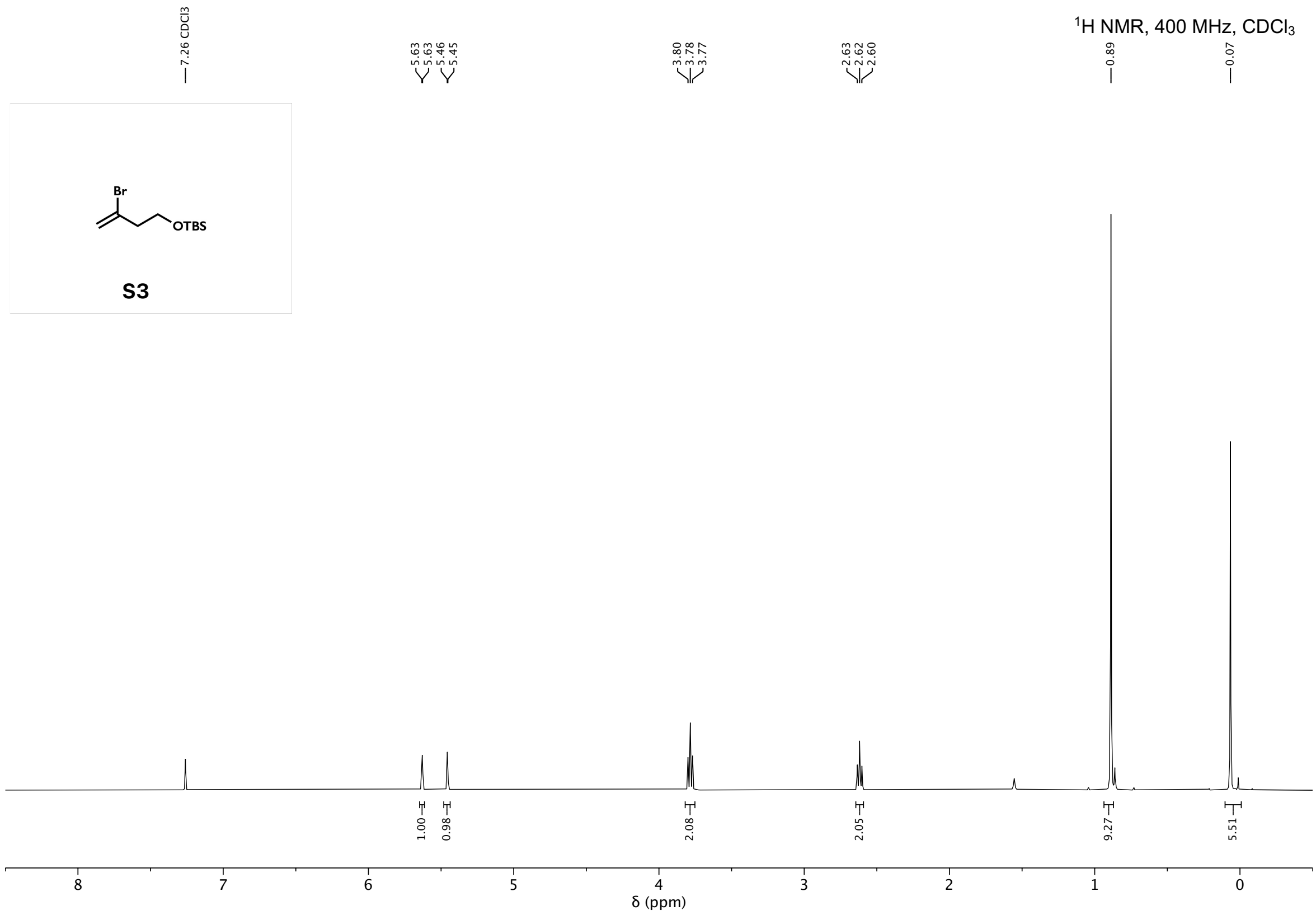

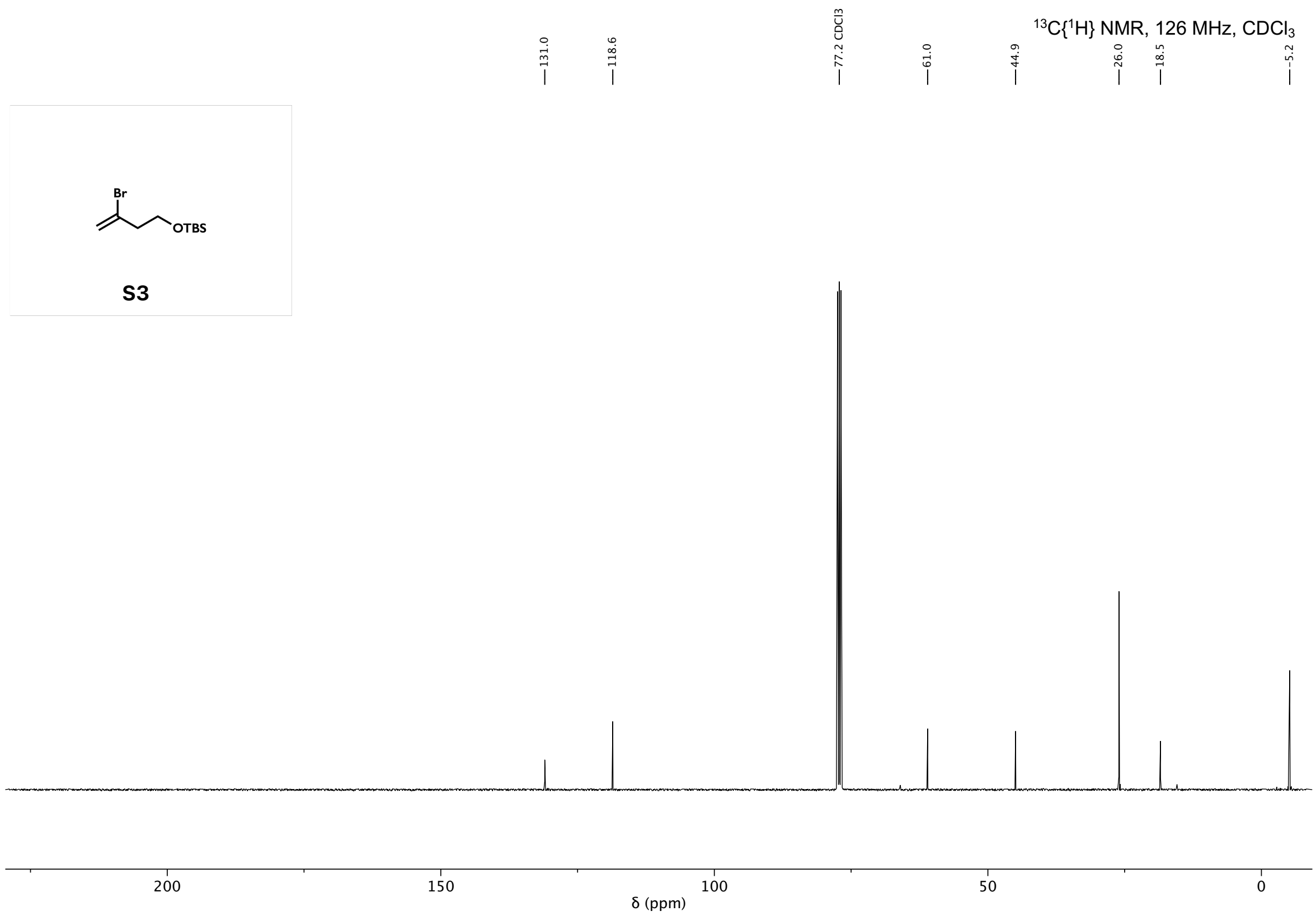

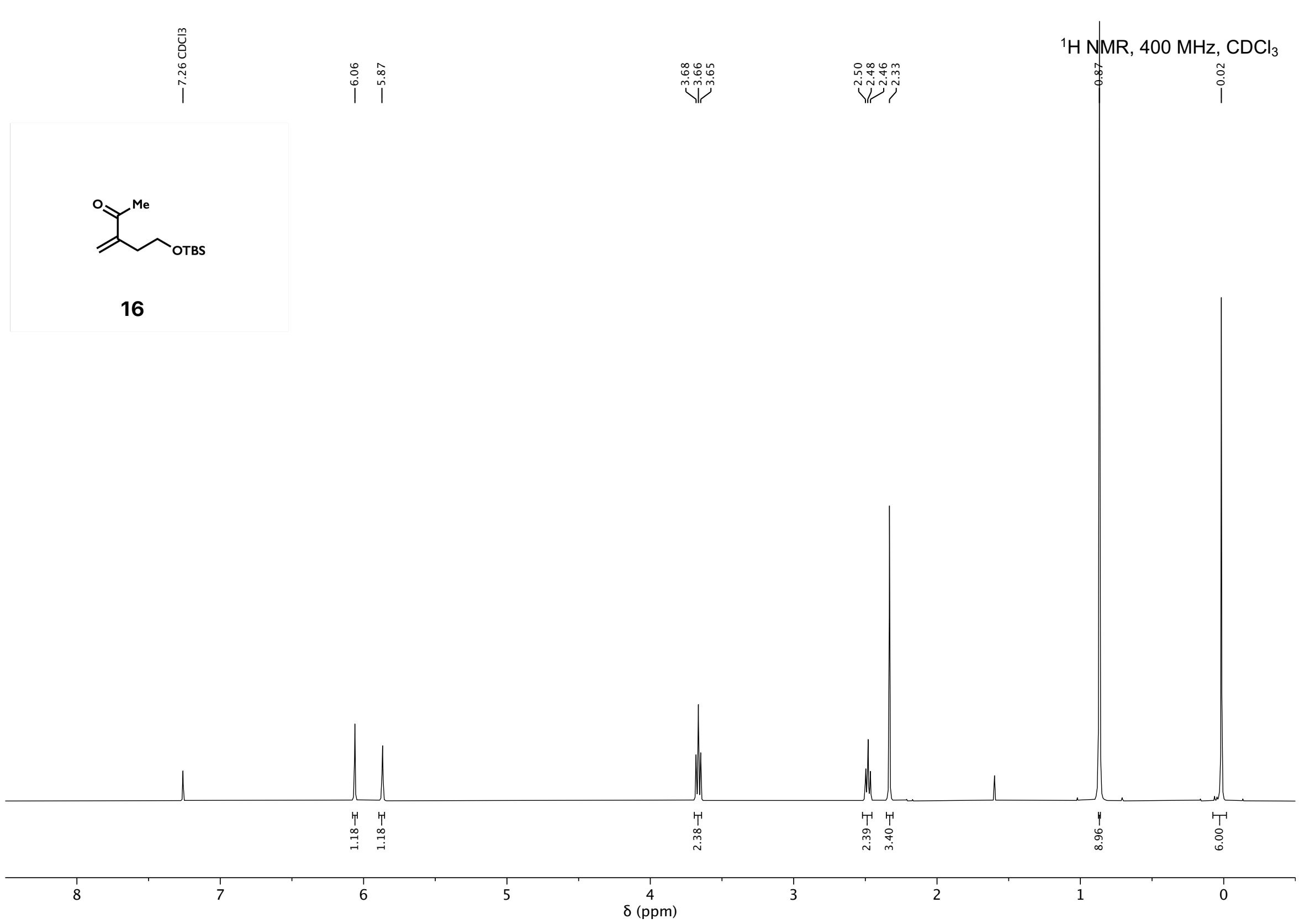
1H NMR, 400 MHz, CDCl3
7.26 CDCl3
6.06
5.87
3.68
3.66
3.65
2.50
2.48
2.46
2.33
0.87
0.02
O
Me
OTBS
16
1.18
1.18
2.38
2.39
3.40
8.96
6.00
8
7
6
5
4
3
2
1
0
δ (ppm)

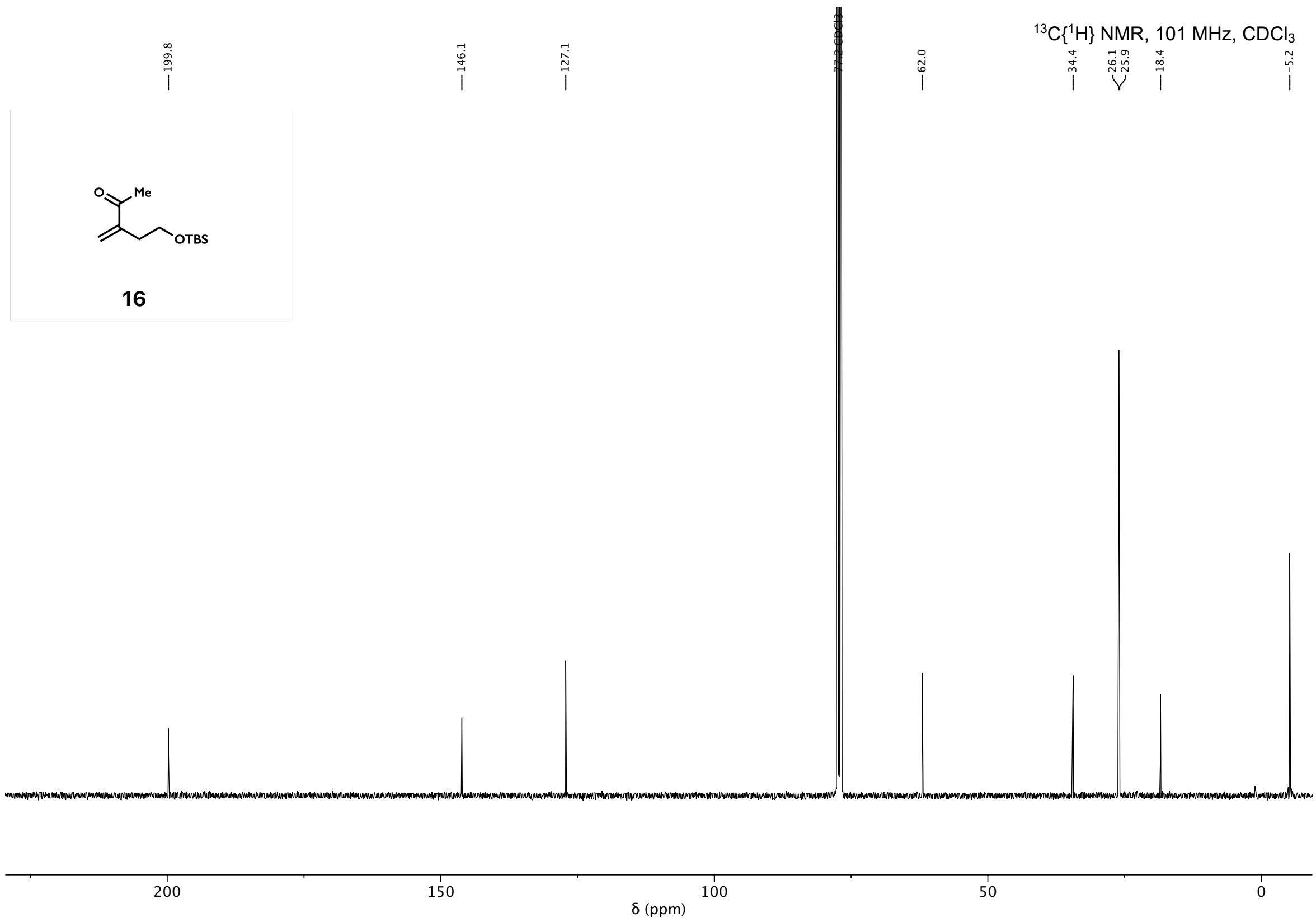
13C{1H} NMR, 101 MHz, CDCl3
199.8
146.1
127.1
62.0
34.4
26.1
25.9
18.4
-5.2
O
Me
OTBS
16
200
150
100
50
0
δ (ppm)

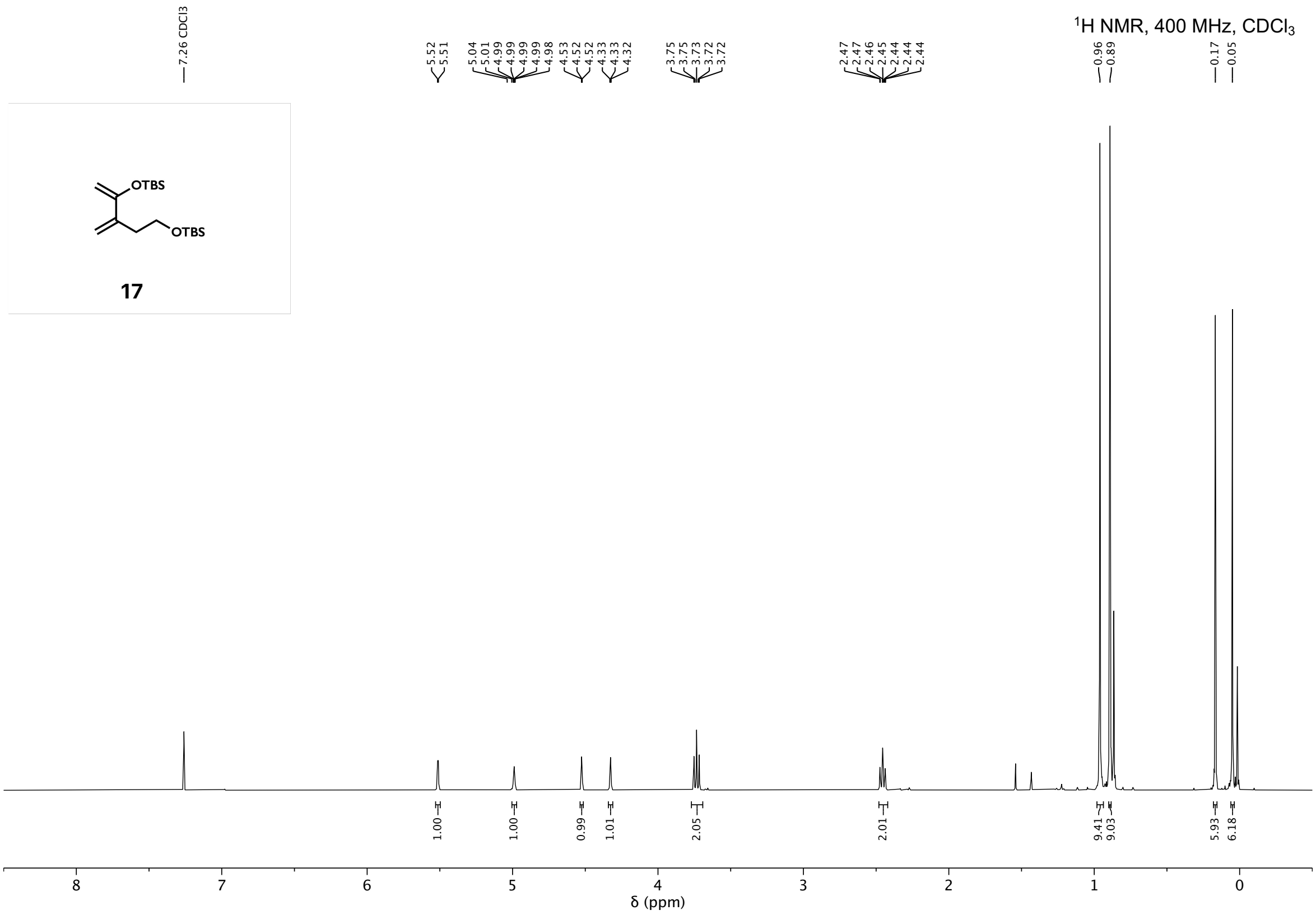
1H NMR, 400 MHz, CDCl3
7.26 CDCl3
5.52
5.51
5.04
5.01
4.99
4.99
4.99
4.99
4.98
4.53
4.52
4.52
4.33
4.33
4.32
3.75
3.75
3.73
3.72
3.72
2.47
2.47
2.46
2.45
2.44
2.44
2.44
0.96
0.89
0.17
0.05
OTBS
OTBS
17
1.00
1.00
0.99
1.01
2.05
2.01
9.41
9.03
5.93
6.18
8
7
6
5
4
3
2
1
0
δ (ppm)

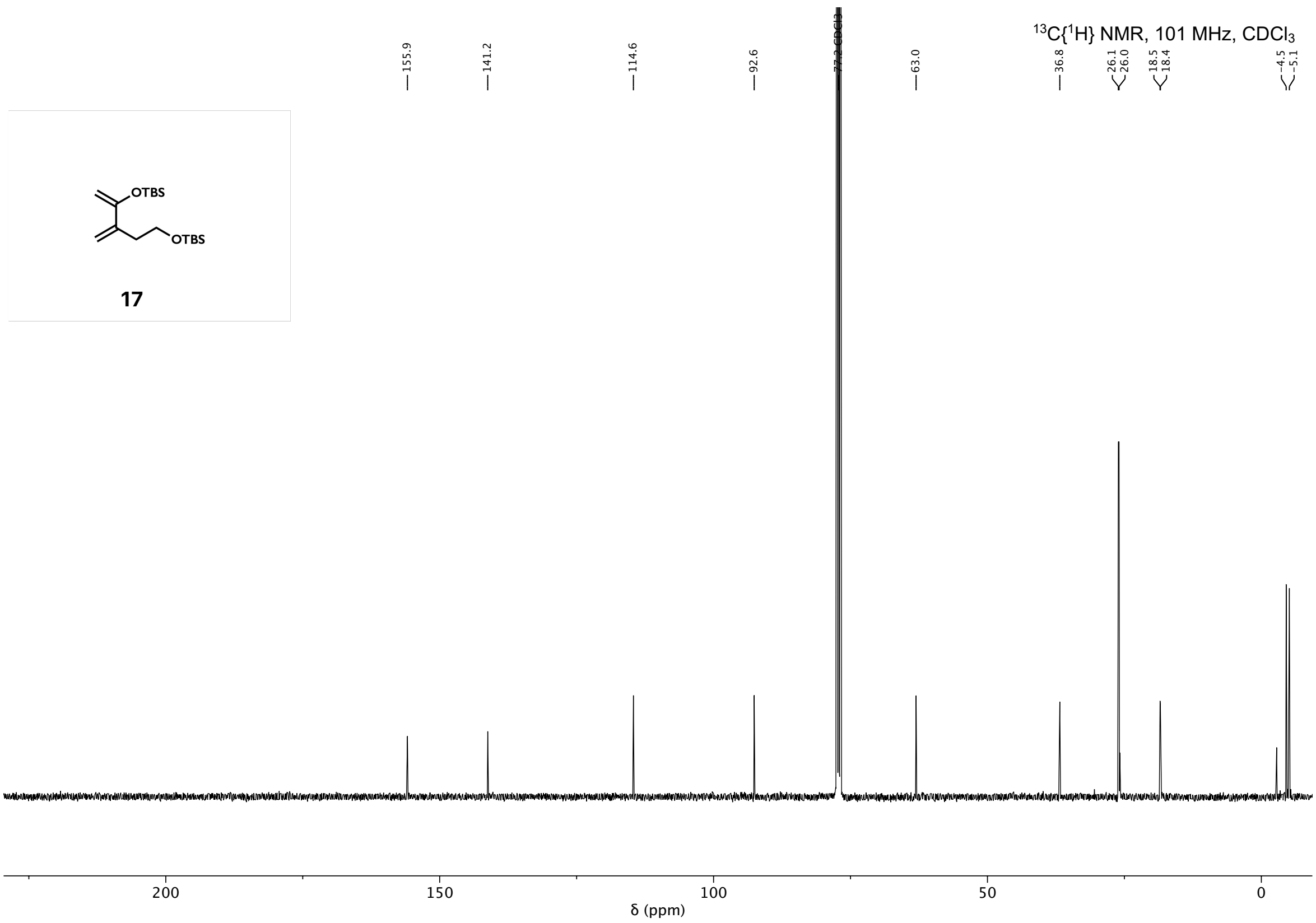
13C{1H} NMR, 101 MHz, CDCl3
155.9
141.2
114.6
92.6
77.2 CDCl3
63.0
36.8
26.1
26.0
18.5
18.4
-4.5
-5.1
OTBS
OTBS
17
200
150
100
50
0
δ (ppm)

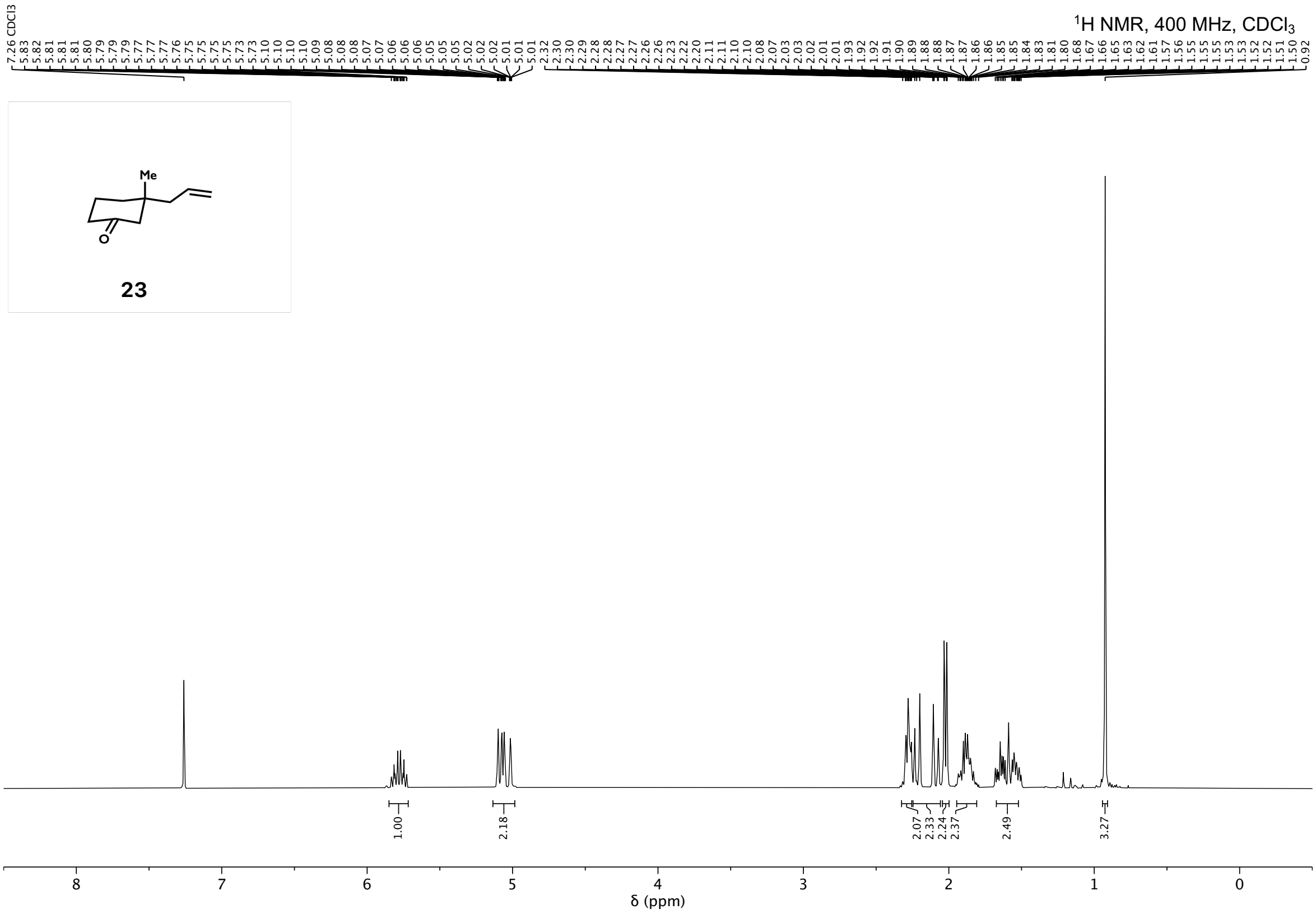

^{1}H NMR, 400 MHz, CDCl$_3$
Me
O
23
1.00
2.18
2.07
2.33
2.24
2.37
2.49
3.27
δ (ppm)

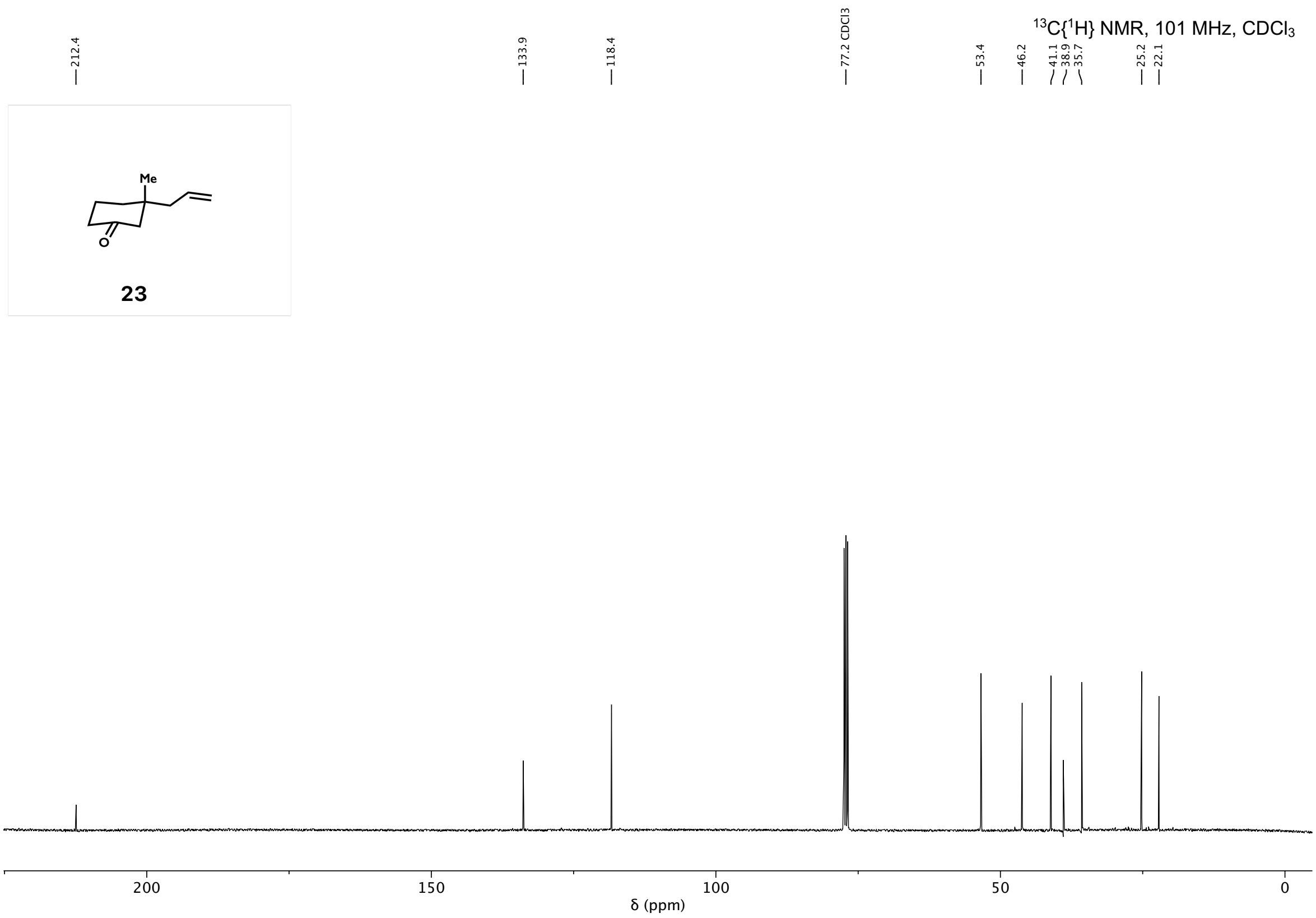

^{13}C{^{1}H} NMR, 101 MHz, CDCl$_3$
212.4
133.9
118.4
77.2 CDCl3
53.4
46.2
41.1
38.9
35.7
25.2
22.1
Me
O
23
δ (ppm)

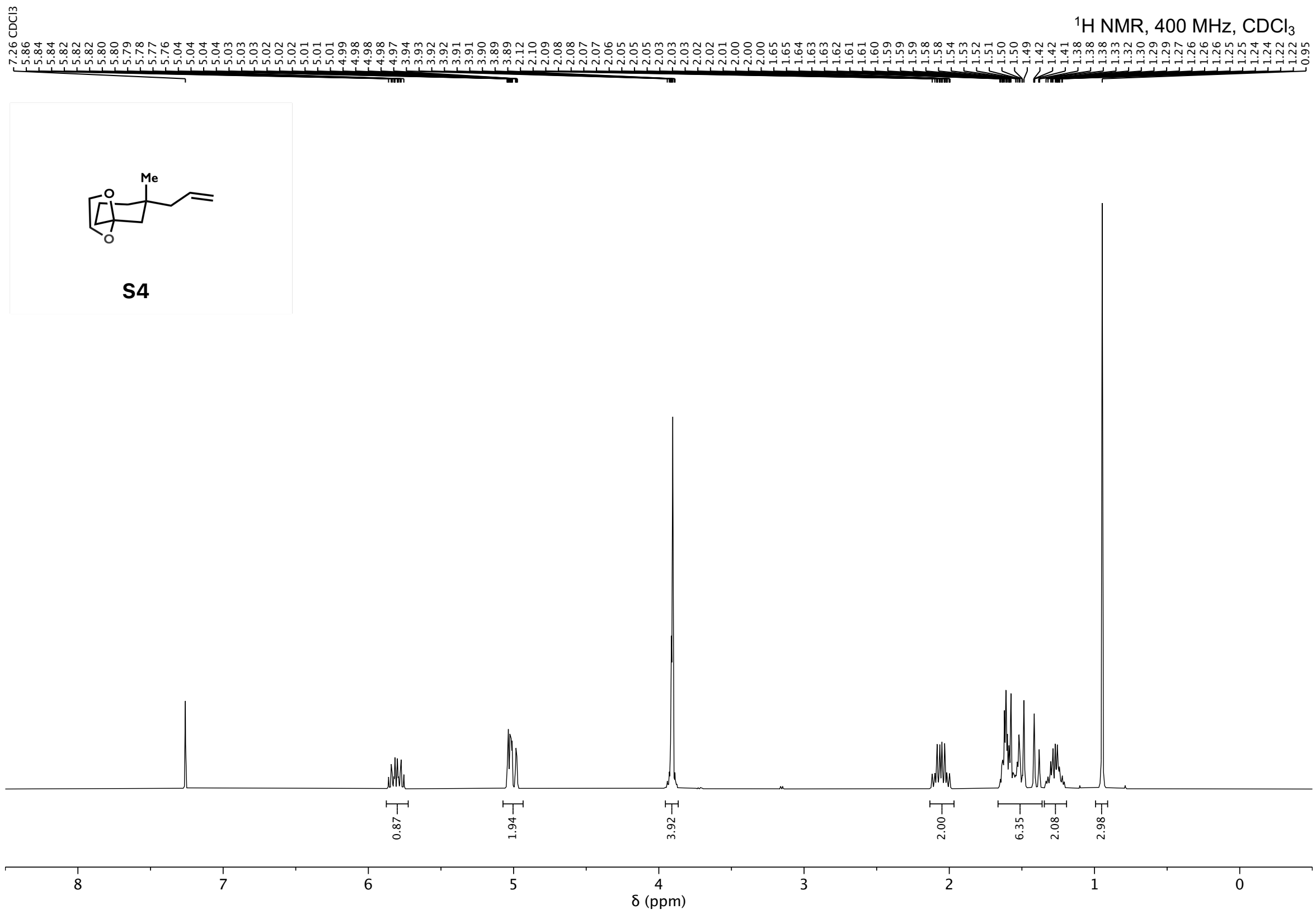
1H NMR, 400 MHz, CDCl3
7.26 CDCl3
5.86
5.84
5.84
5.82
5.82
5.80
5.80
5.79
5.78
5.77
5.76
5.04
5.04
5.04
5.03
5.03
5.02
5.02
5.02
5.01
5.01
5.01
4.99
4.98
4.98
4.98
4.97
3.94
3.93
3.92
3.92
3.91
3.91
3.90
3.89
2.12
2.10
2.09
2.08
2.08
2.07
2.07
2.06
2.05
2.05
2.05
2.03
2.03
2.03
2.02
2.02
2.01
2.00
2.00
1.65
1.65
1.64
1.63
1.62
1.61
1.61
1.60
1.59
1.59
1.59
1.58
1.58
1.54
1.53
1.52
1.51
1.50
1.50
1.49
1.42
1.42
1.41
1.38
1.38
1.38
1.33
1.32
1.30
1.29
1.29
1.27
1.26
1.26
1.25
1.25
1.24
1.24
1.22
1.22
0.95
Me
O
O
S4
0.87
1.94
3.92
2.00
6.35
2.08
2.98
8
7
6
5
4
3
2
1
0
δ (ppm)

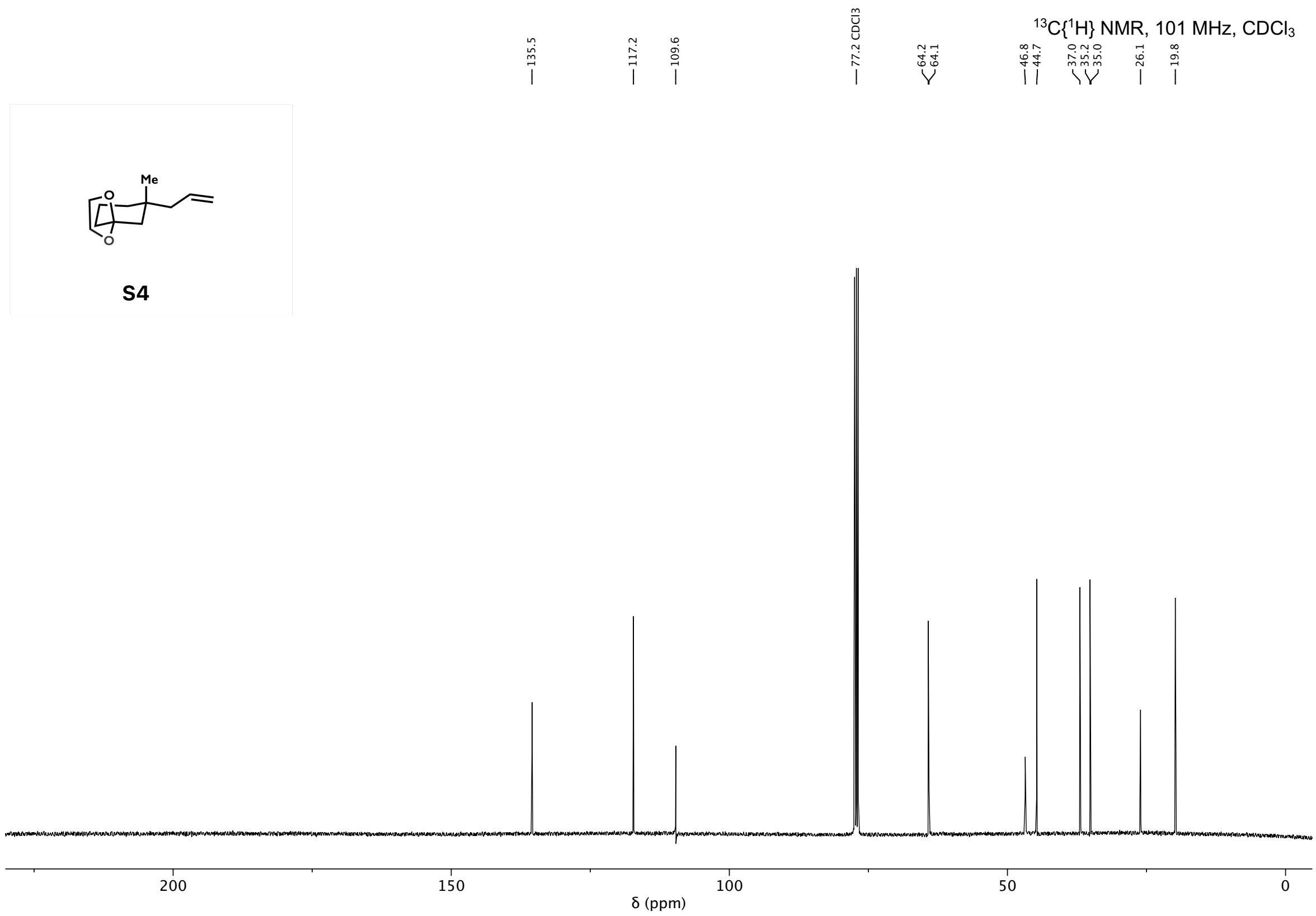
13C{1H} NMR, 101 MHz, CDCl3
135.5
117.2
109.6
77.2 CDCl3
64.2
64.1
46.8
44.7
37.0
35.2
35.0
26.1
19.8
Me
O
O
S4
200
150
100
50
0
δ (ppm)

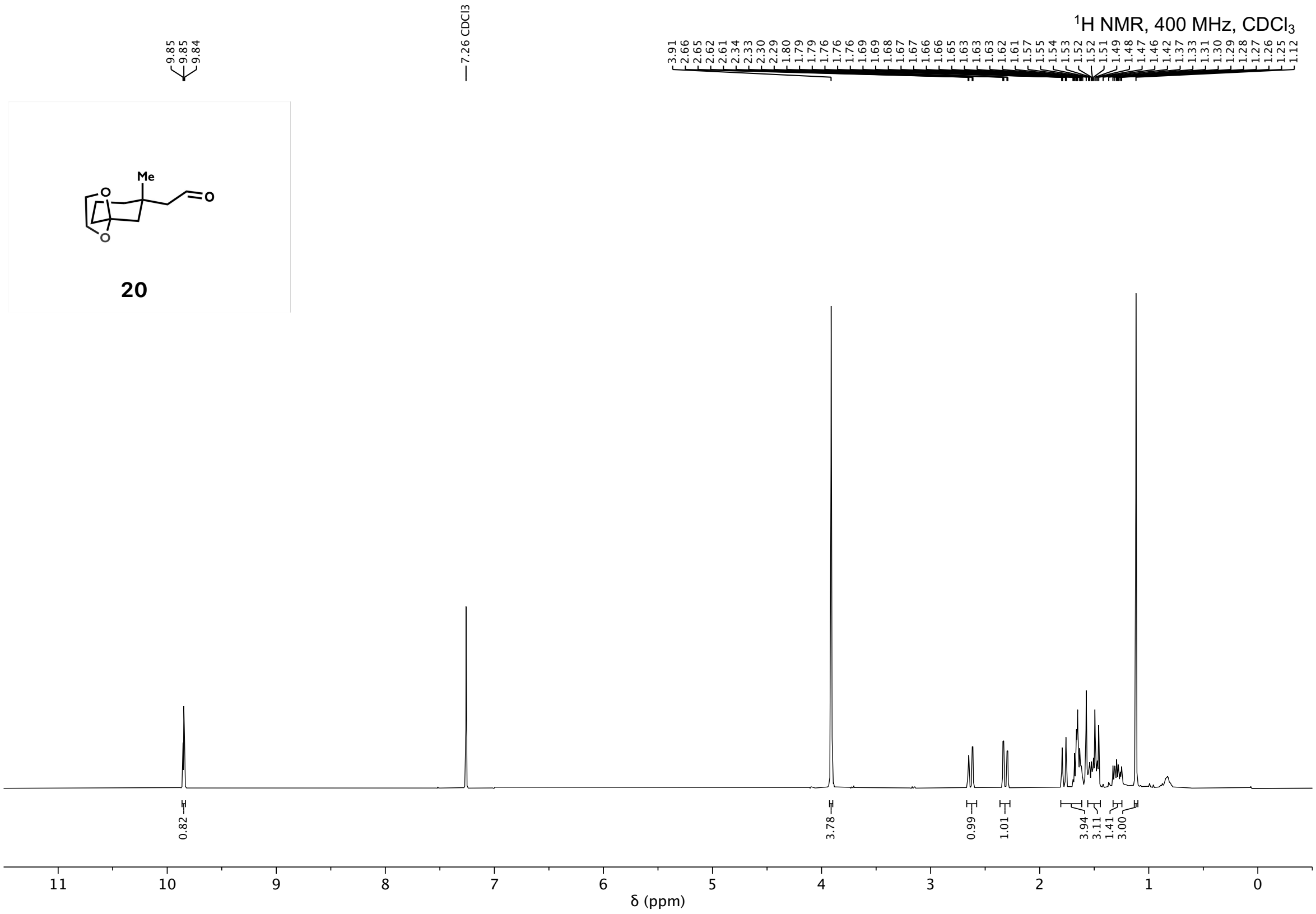

¹H NMR, 400 MHz, CDCl3
20
Me
O
δ (ppm)

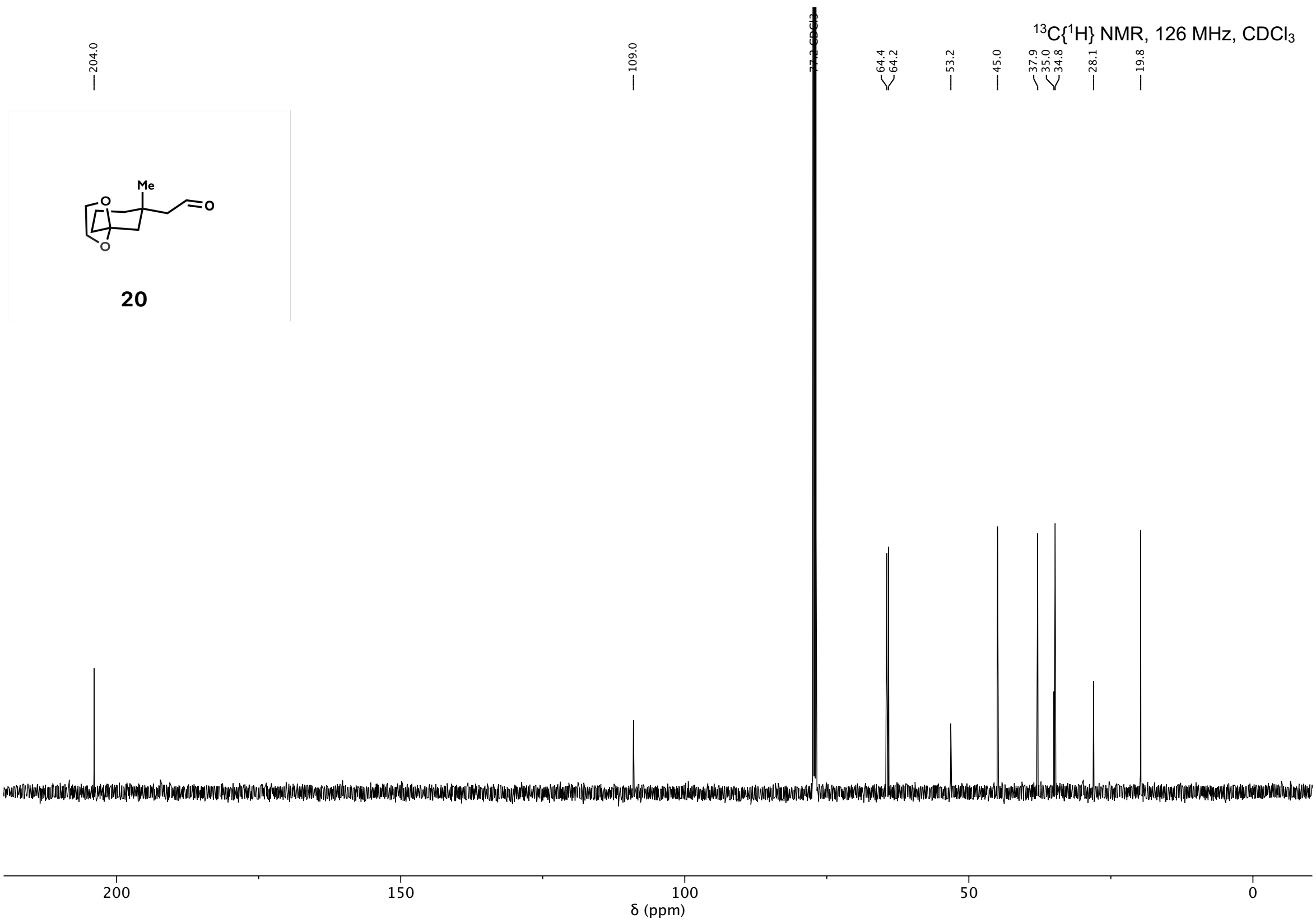

¹³C{¹H} NMR, 126 MHz, CDCl3
20
Me
O
204.0
109.0
64.4
64.2
53.2
45.0
37.9
35.0
34.8
28.1
19.8
δ (ppm)

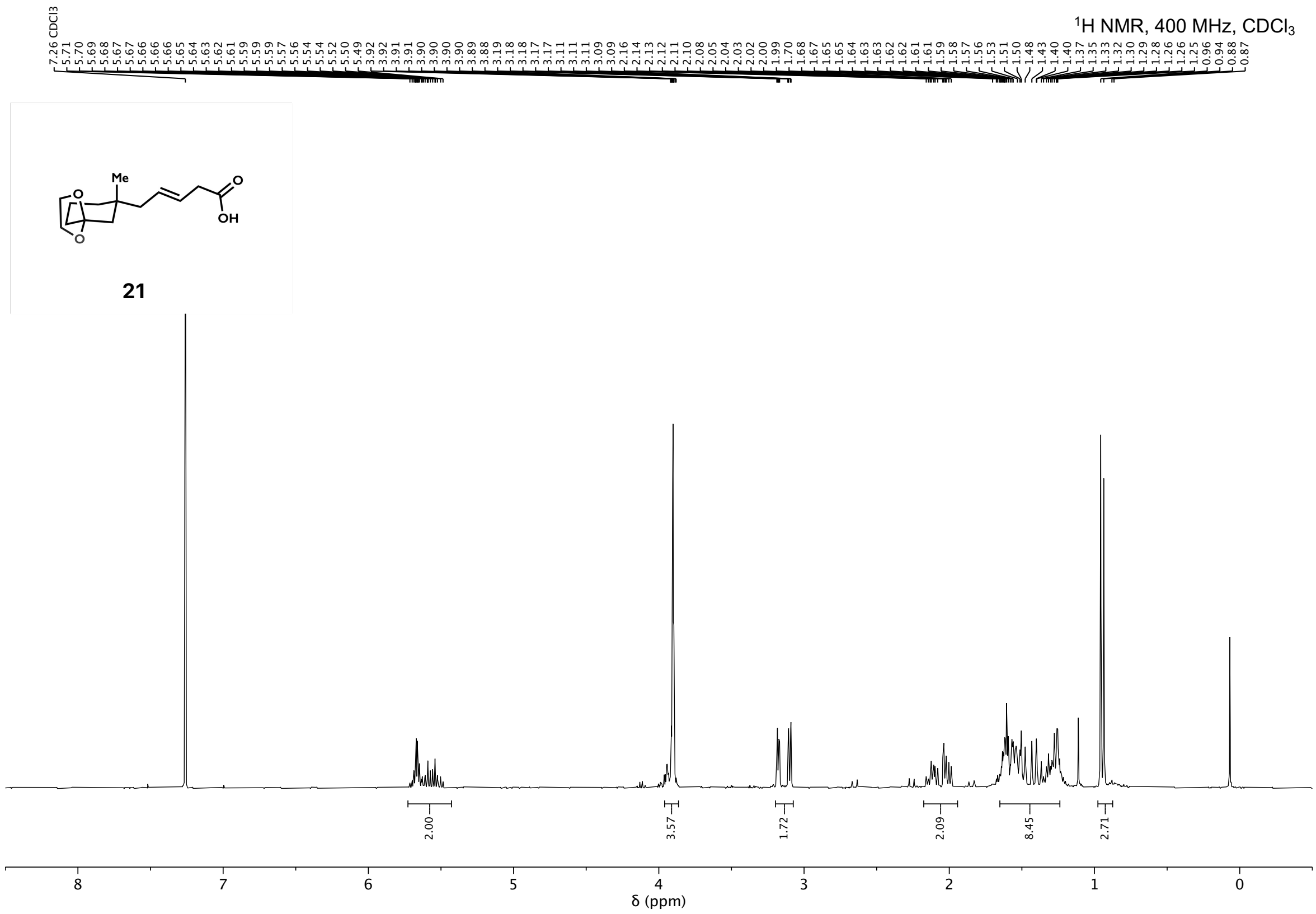

^{1}H NMR, 400 MHz, $CDCl_3$
7.26 CDCl3
Me
O
OH
21
2.00
3.57
1.72
2.09
8.45
2.71
δ (ppm)

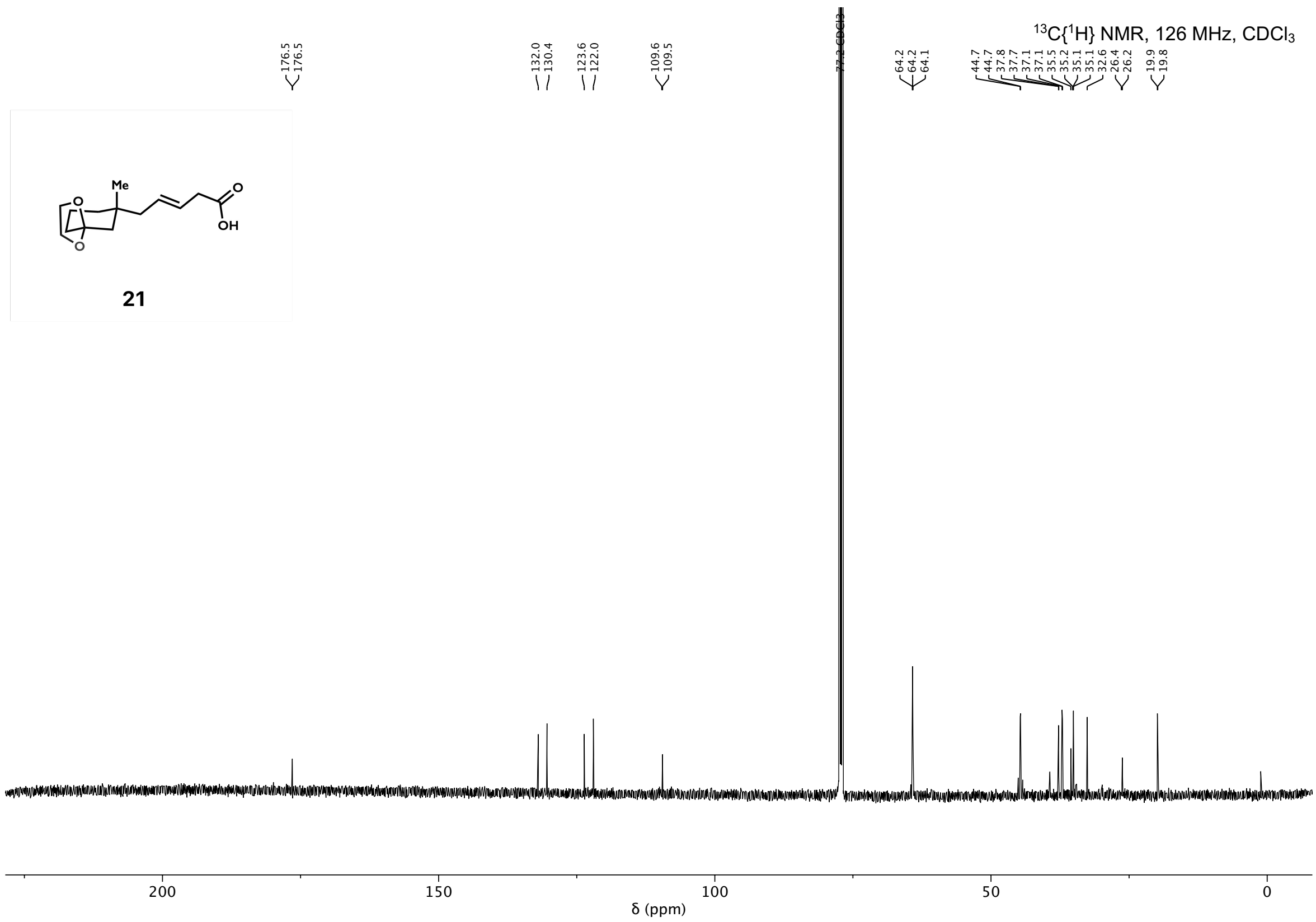

^{13}C{^{1}H} NMR, 126 MHz, $CDCl_3$
176.5
176.5
132.0
130.4
123.6
122.0
109.6
109.5
64.2
64.2
64.1
44.7
44.7
37.8
37.7
37.1
35.5
35.2
35.1
35.1
32.6
26.4
26.2
19.9
19.8
Me
O
OH
21
δ (ppm)

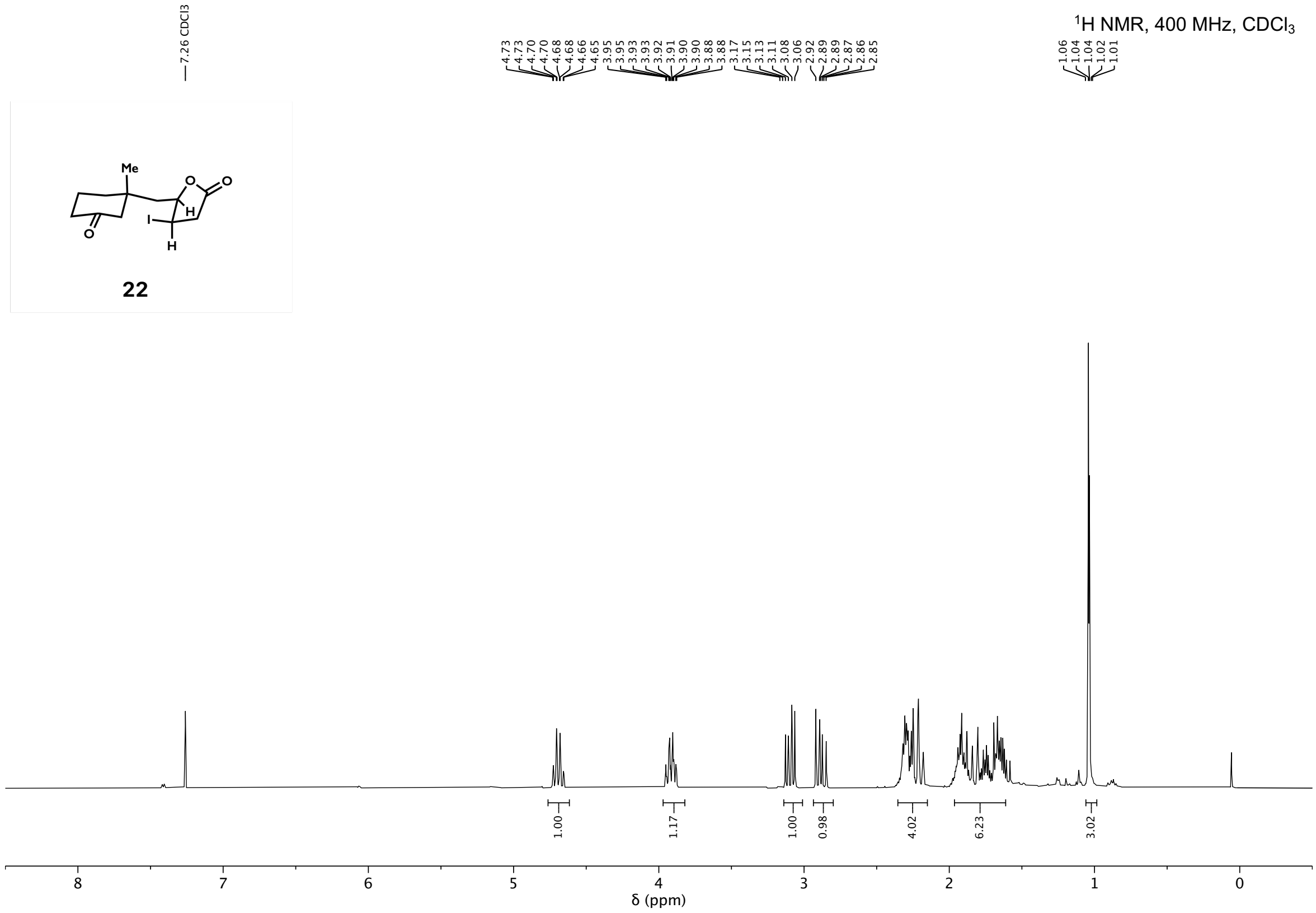
1H NMR, 400 MHz, CDCl3
7.26 CDCl3
4.73 4.73 4.70 4.68 4.68 4.66 4.65 3.95 3.95 3.93 3.93 3.92 3.91 3.90 3.90 3.88 3.17 3.15 3.13 3.11 3.08 3.06 2.92 2.89 2.89 2.87 2.86 2.85
1.06 1.04 1.04 1.02 1.01
Me
O
O
I
H
H
O
22
1.00
1.17
1.00
0.98
4.02
6.23
3.02
8 7 6 5 4 3 2 1 0
δ (ppm)

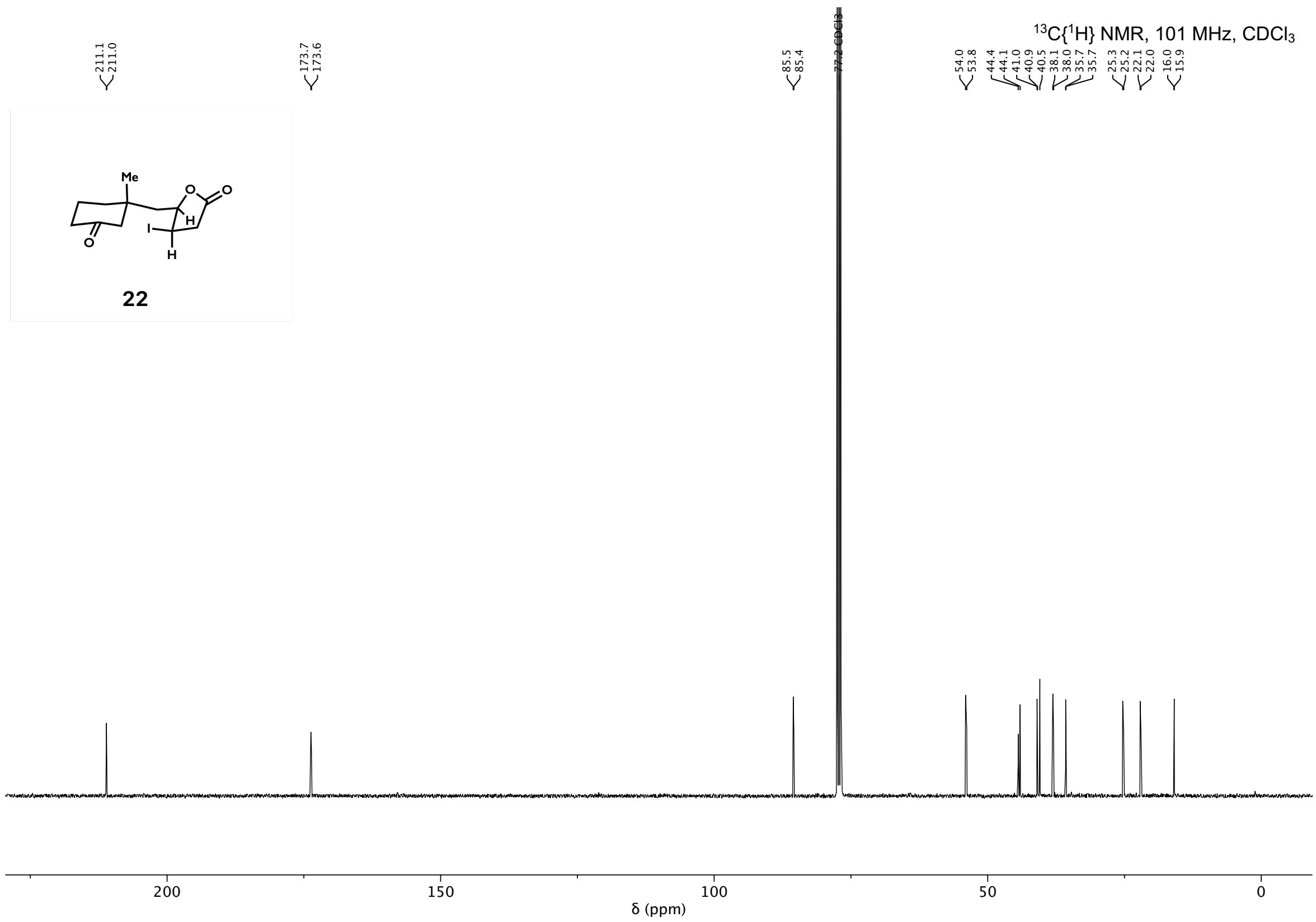
13C{1H} NMR, 101 MHz, CDCl3
211.1 211.0
173.7 173.6
85.5 85.4
77.2 CDCl3
54.0 53.8 44.4 44.1 41.0 40.9 40.5 38.1 38.0 35.7 35.7 25.3 25.2 22.1 22.0 16.0 15.9
Me
O
O
I
H
H
O
22
200 150 100 50 0
δ (ppm)

^{1}H NMR, 400 MHz, $CDCl_3$

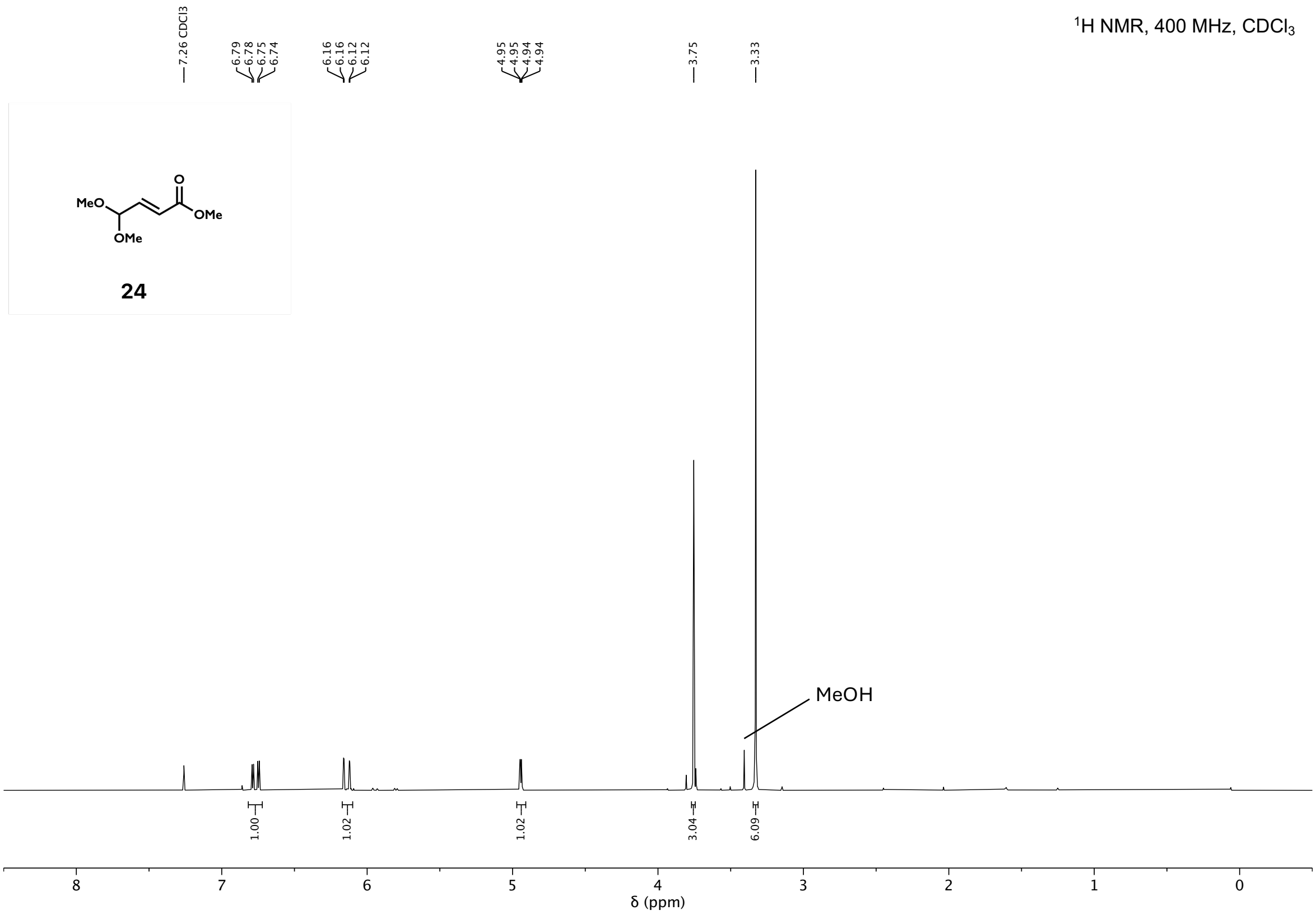

$^{13}C\{^{1}H\}$ NMR, 101 MHz, $CDCl_3$

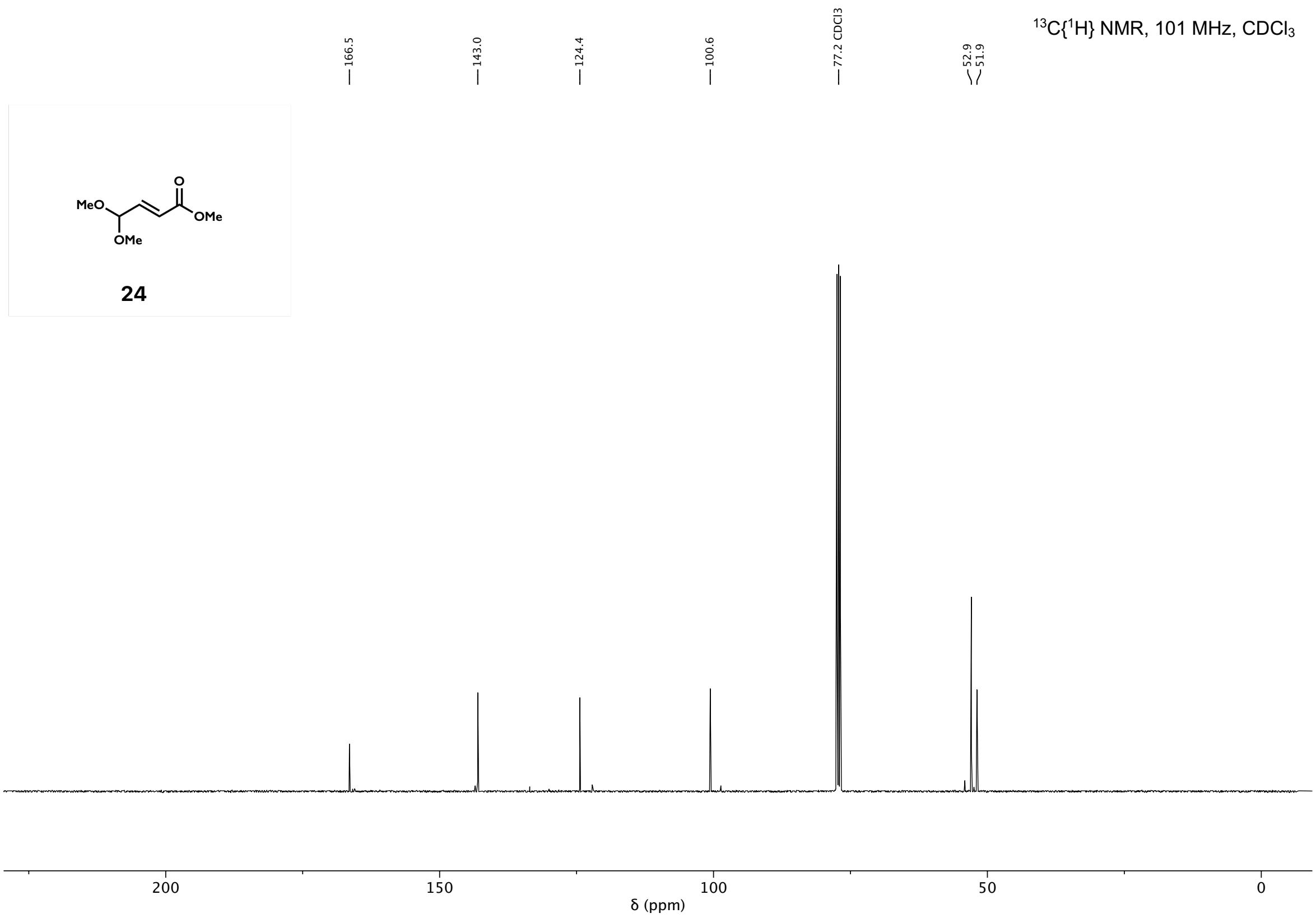

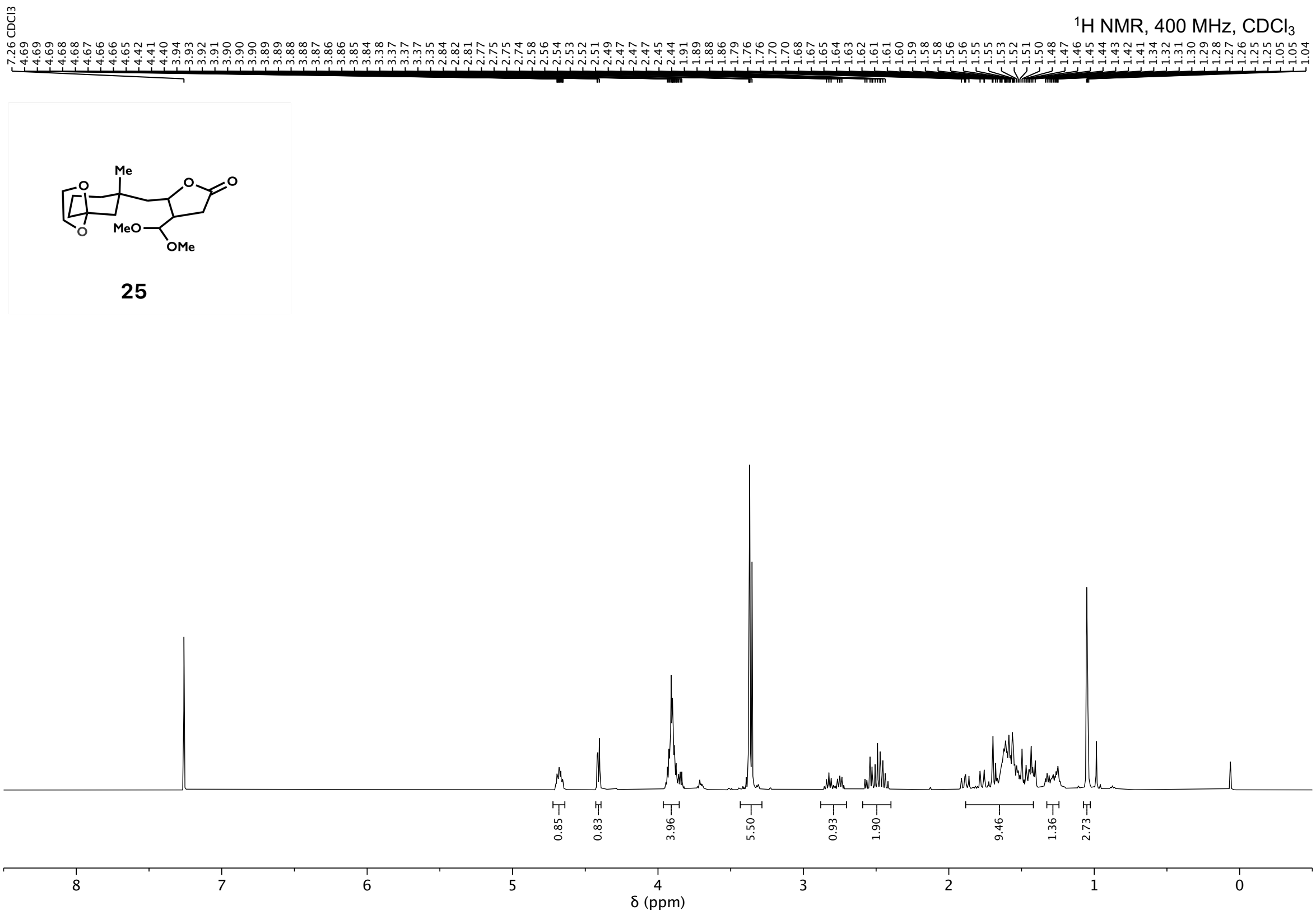

1H NMR, 400 MHz, CDCl3
7.26 CDCl3
4.69
4.69
4.69
4.68
4.68
4.67
4.66
4.66
4.65
4.42
4.41
4.40
3.94
3.93
3.92
3.91
3.90
3.90
3.89
3.89
3.88
3.88
3.87
3.86
3.86
3.85
3.84
3.38
3.37
3.37
3.35
2.84
2.82
2.81
2.77
2.75
2.75
2.74
2.58
2.56
2.54
2.53
2.52
2.51
2.49
2.47
2.47
2.47
2.45
2.44
1.91
1.89
1.88
1.86
1.79
1.76
1.76
1.70
1.70
1.68
1.67
1.65
1.64
1.63
1.62
1.61
1.61
1.60
1.59
1.58
1.58
1.56
1.56
1.55
1.55
1.53
1.52
1.51
1.50
1.48
1.47
1.46
1.45
1.44
1.43
1.42
1.41
1.34
1.32
1.31
1.30
1.29
1.28
1.27
1.26
1.25
1.25
1.05
1.05
1.04
0.85
0.83
3.96
5.50
0.93
1.90
9.46
1.36
2.73
8
7
6
5
4
3
2
1
0
δ (ppm)

25

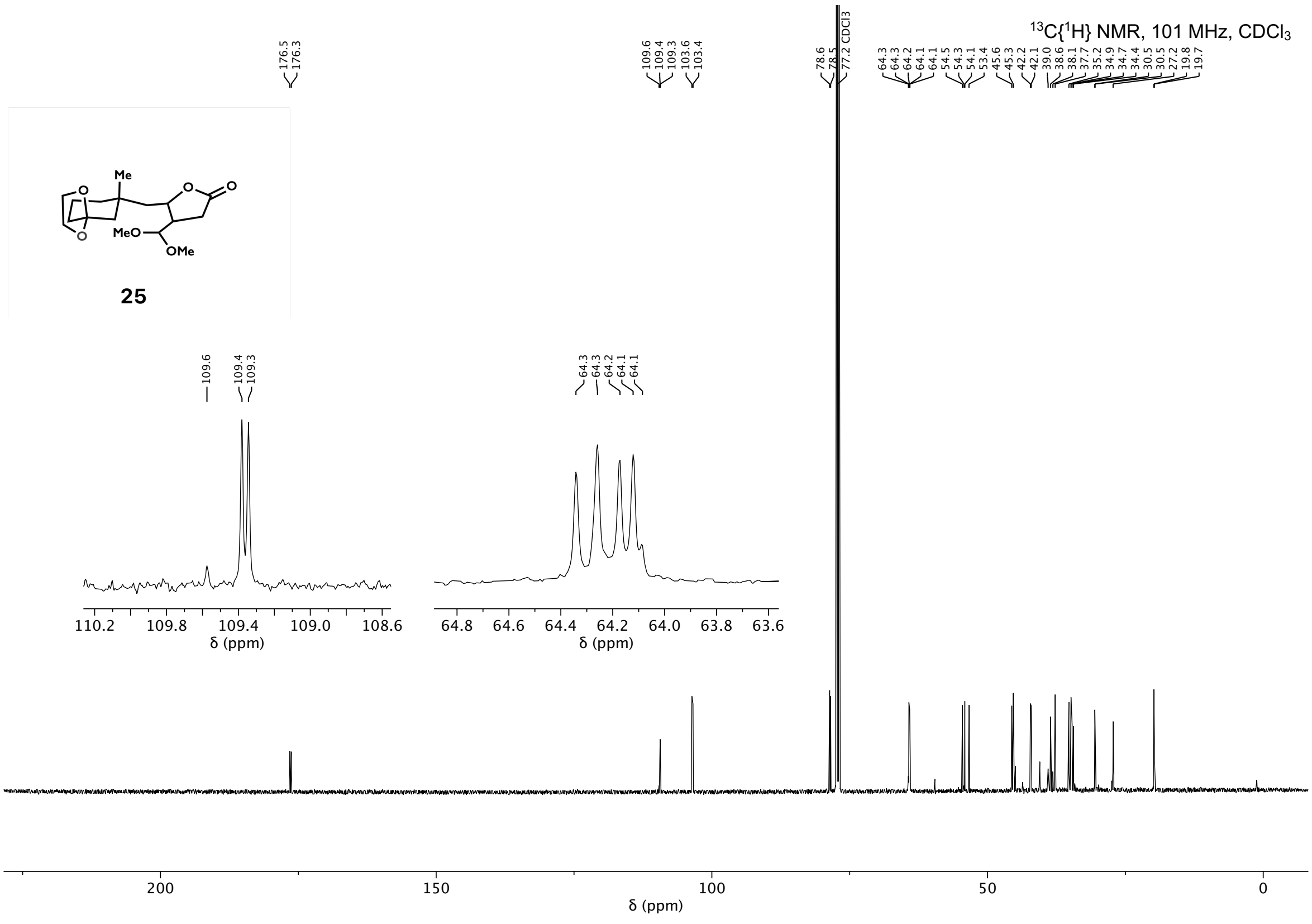

13C{1H} NMR, 101 MHz, CDCl3
176.5
176.3
109.6
109.4
109.3
103.6
103.4
78.6
78.5
77.2 CDCl3
64.3
64.3
64.2
64.1
64.1
54.5
54.3
54.1
53.4
45.6
45.3
42.2
42.1
39.0
38.6
38.1
37.7
35.2
34.9
34.7
34.4
30.5
30.5
27.2
19.8
19.7
109.6
109.4
109.3
110.2
109.8
109.4
109.0
108.6
δ (ppm)
64.3
64.3
64.2
64.1
64.1
64.8
64.6
64.4
64.2
64.0
63.8
63.6
δ (ppm)
200
150
100
50
0
δ (ppm)

25

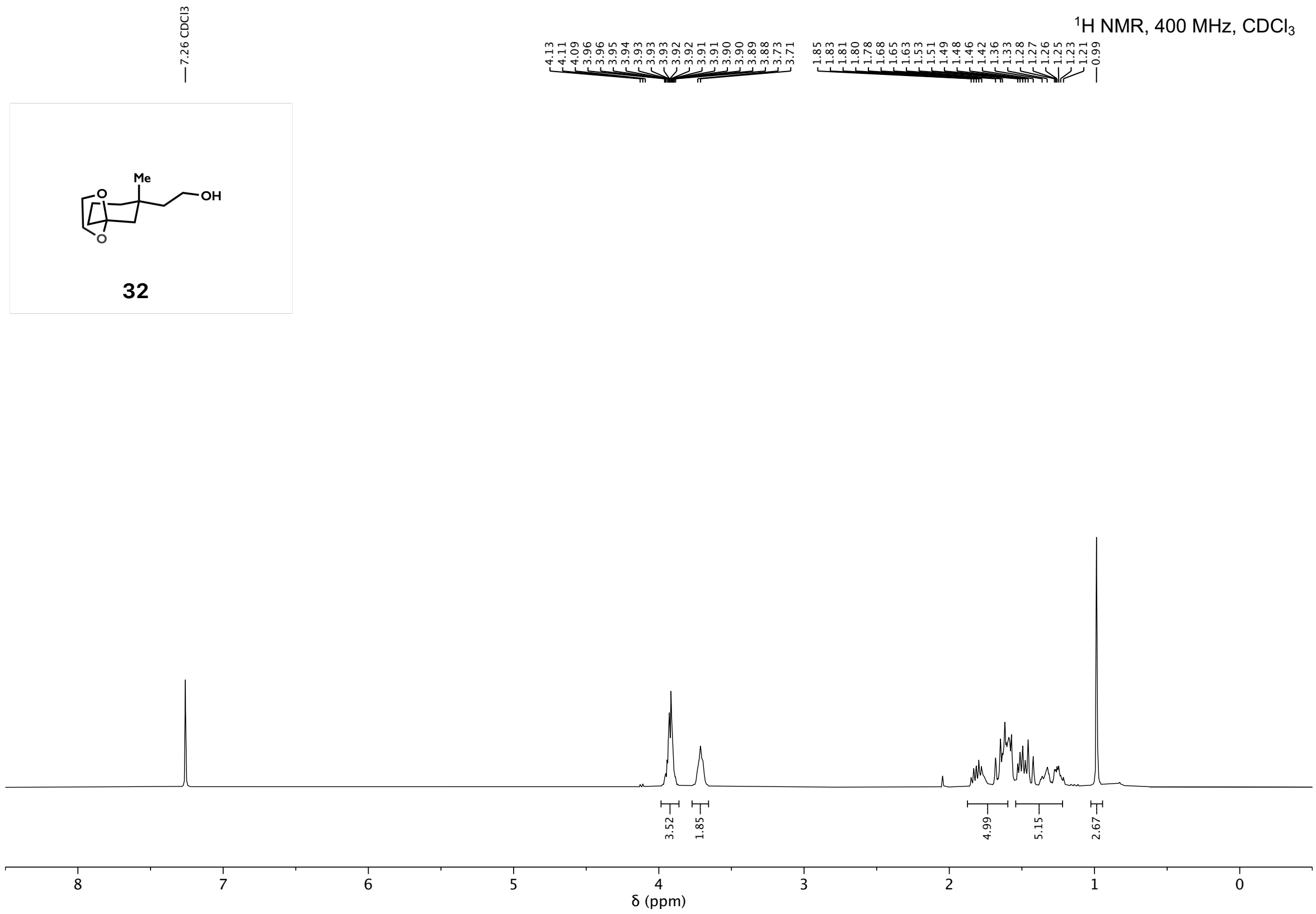

¹H NMR, 400 MHz, CDCl₃
7.26 CDCl3
Me
O
OH
O
32
3.52
1.85
4.99
5.15
2.67
δ (ppm)

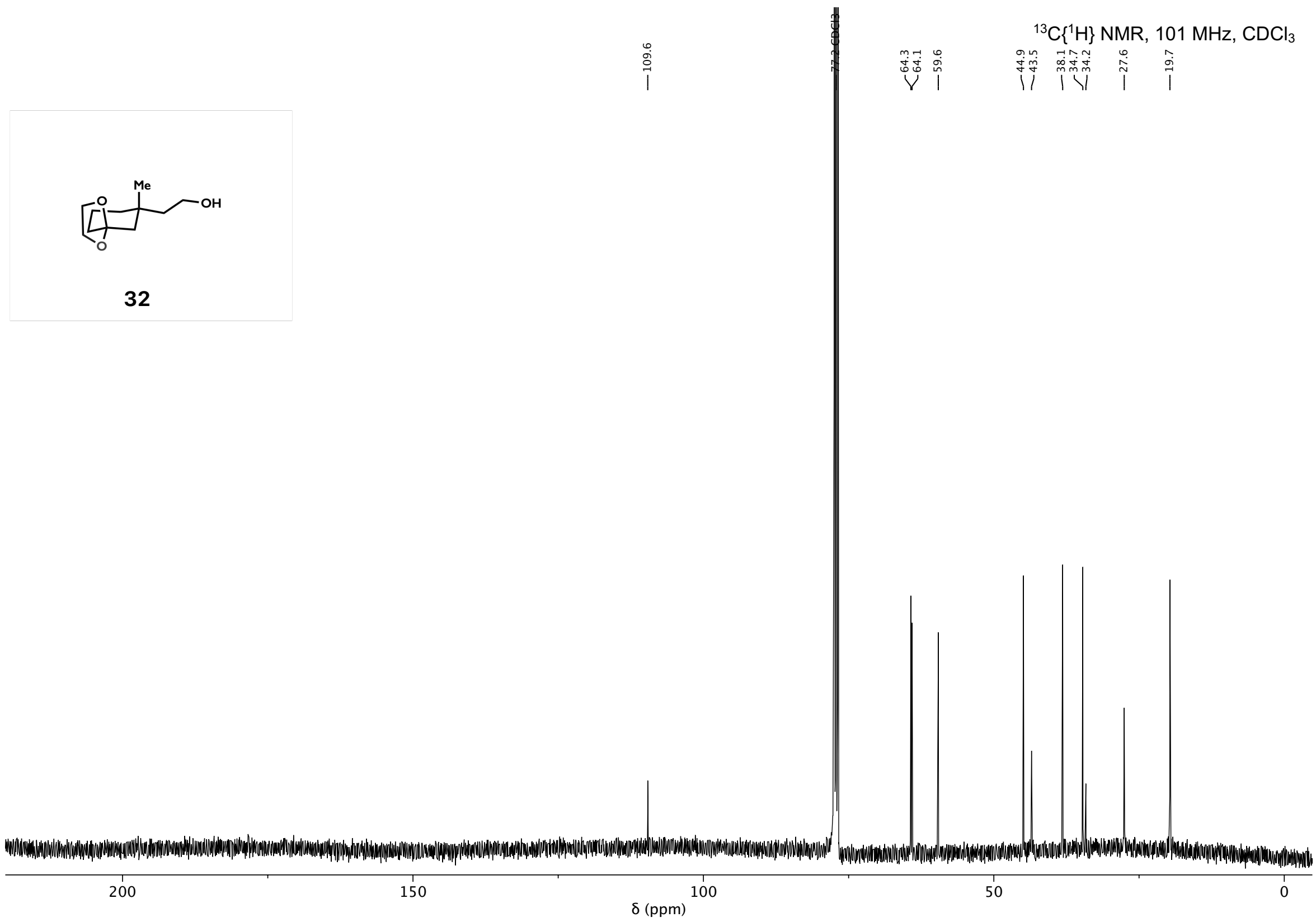

¹³C{¹H} NMR, 101 MHz, CDCl₃
109.6
64.3
64.1
59.6
44.9
43.5
38.1
34.7
34.2
27.6
19.7
Me
O
OH
O
32
δ (ppm)

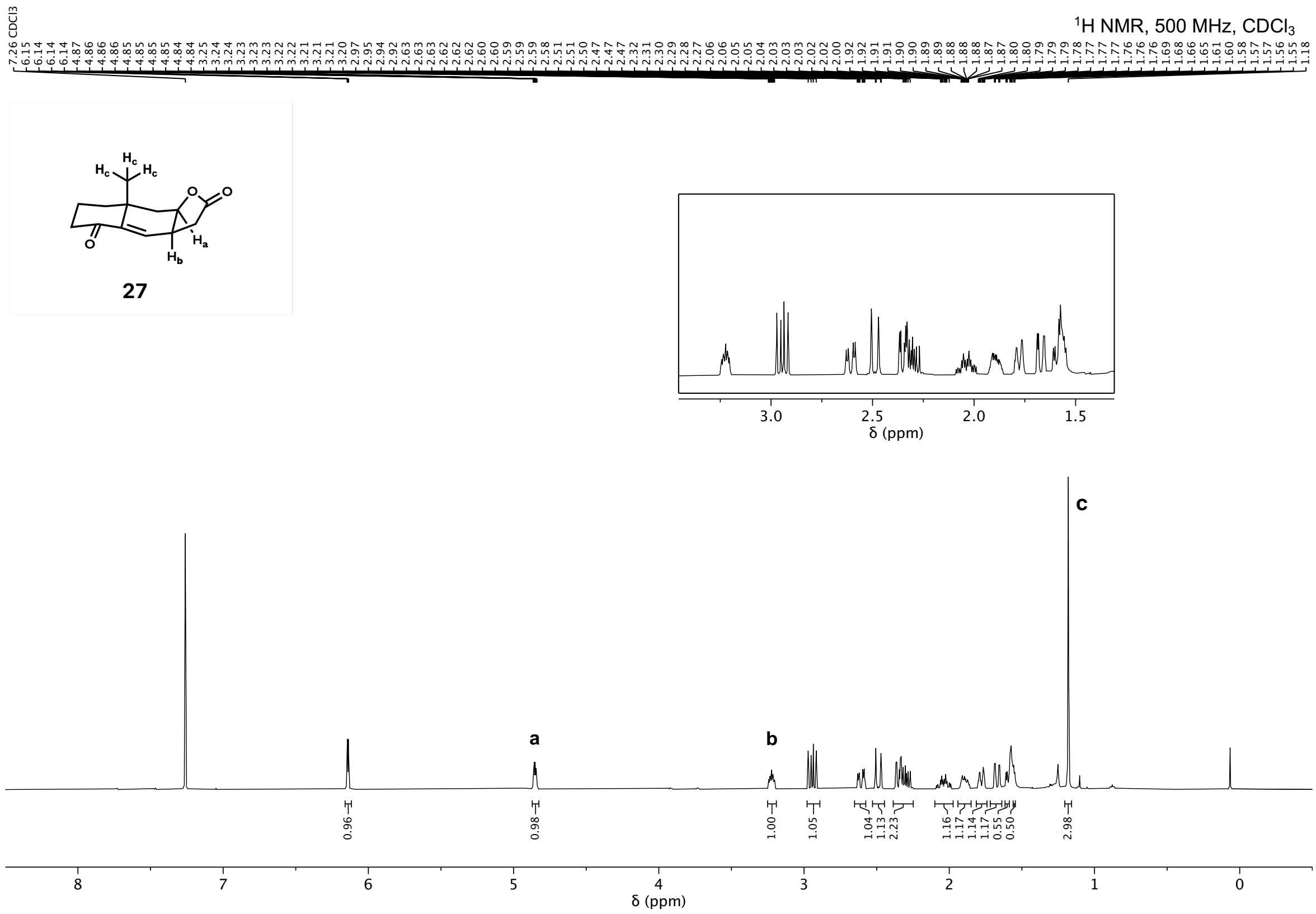

1H NMR, 500 MHz, CDCl3
H_c
H_c
H_c
O
O
O
H_a
H_b
27
a
b
c
δ (ppm)

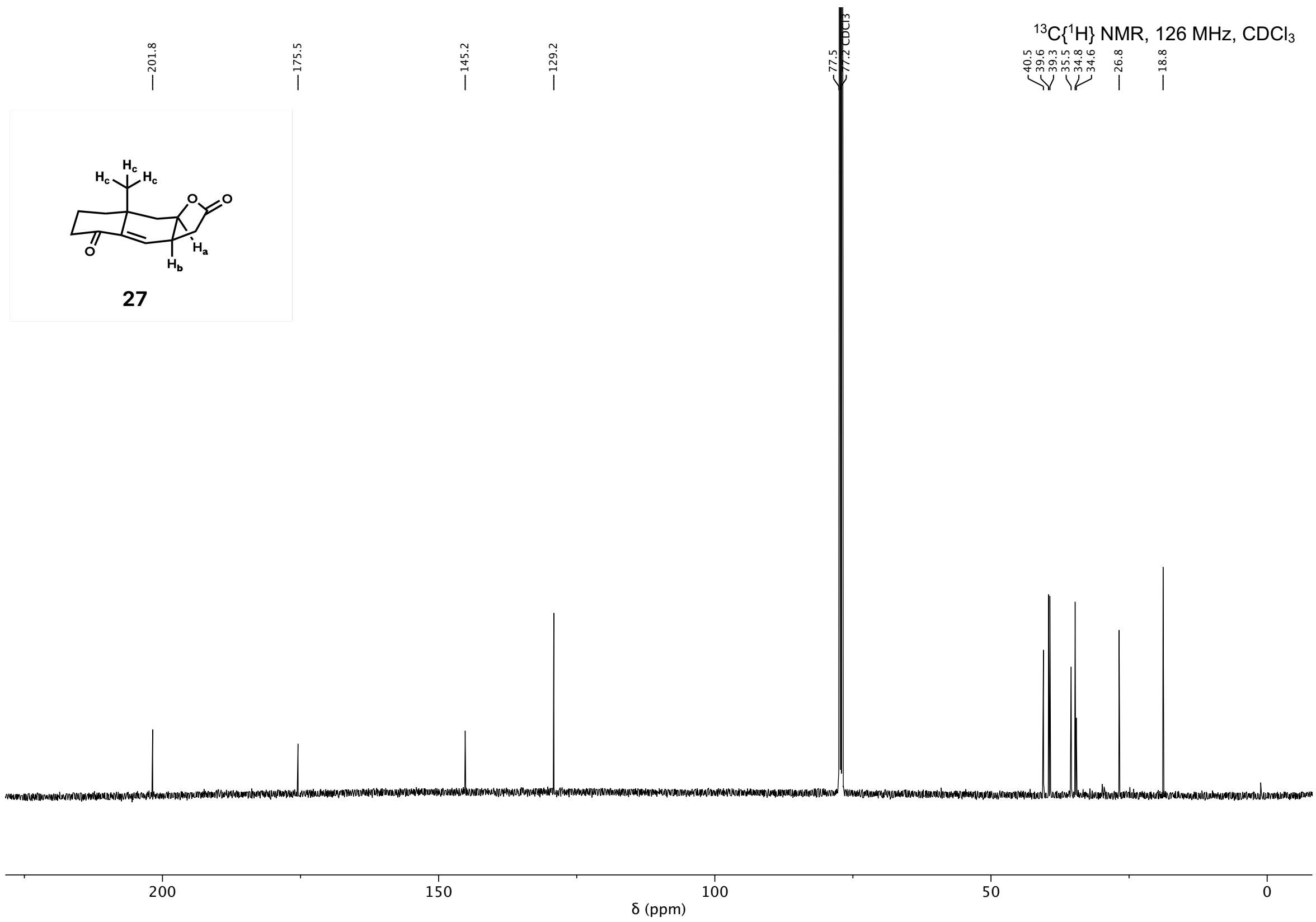

13C{1H} NMR, 126 MHz, CDCl3
201.8
175.5
145.2
129.2
77.5
77.2 CDCl3
40.5
39.6
39.3
35.5
34.8
34.6
26.8
18.8
H_c
H_c
H_c
O
O
O
H_a
H_b
27
δ (ppm)

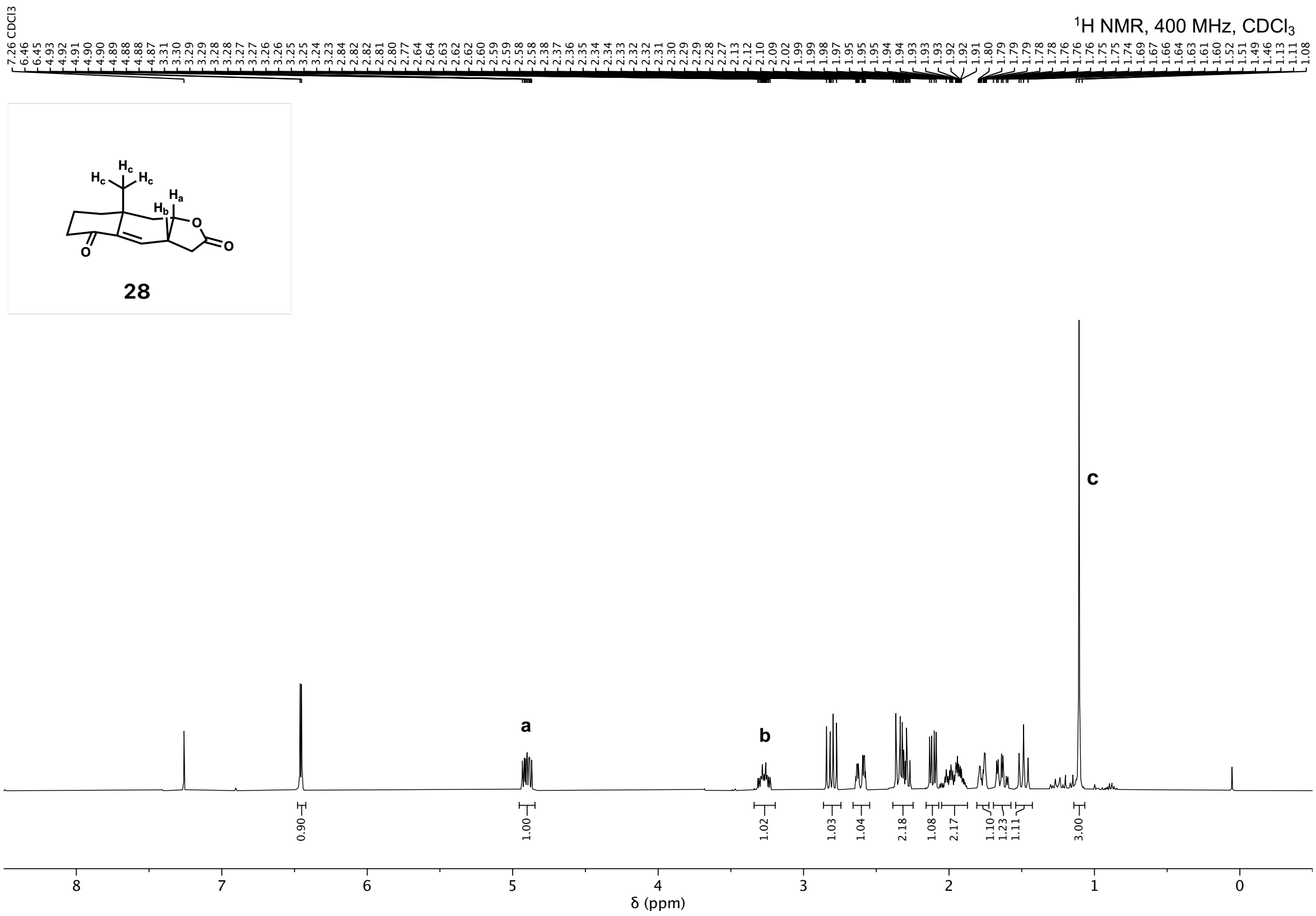

^{1}H NMR, 400 MHz, CDCl$_3$
28
a
b
c
δ (ppm)

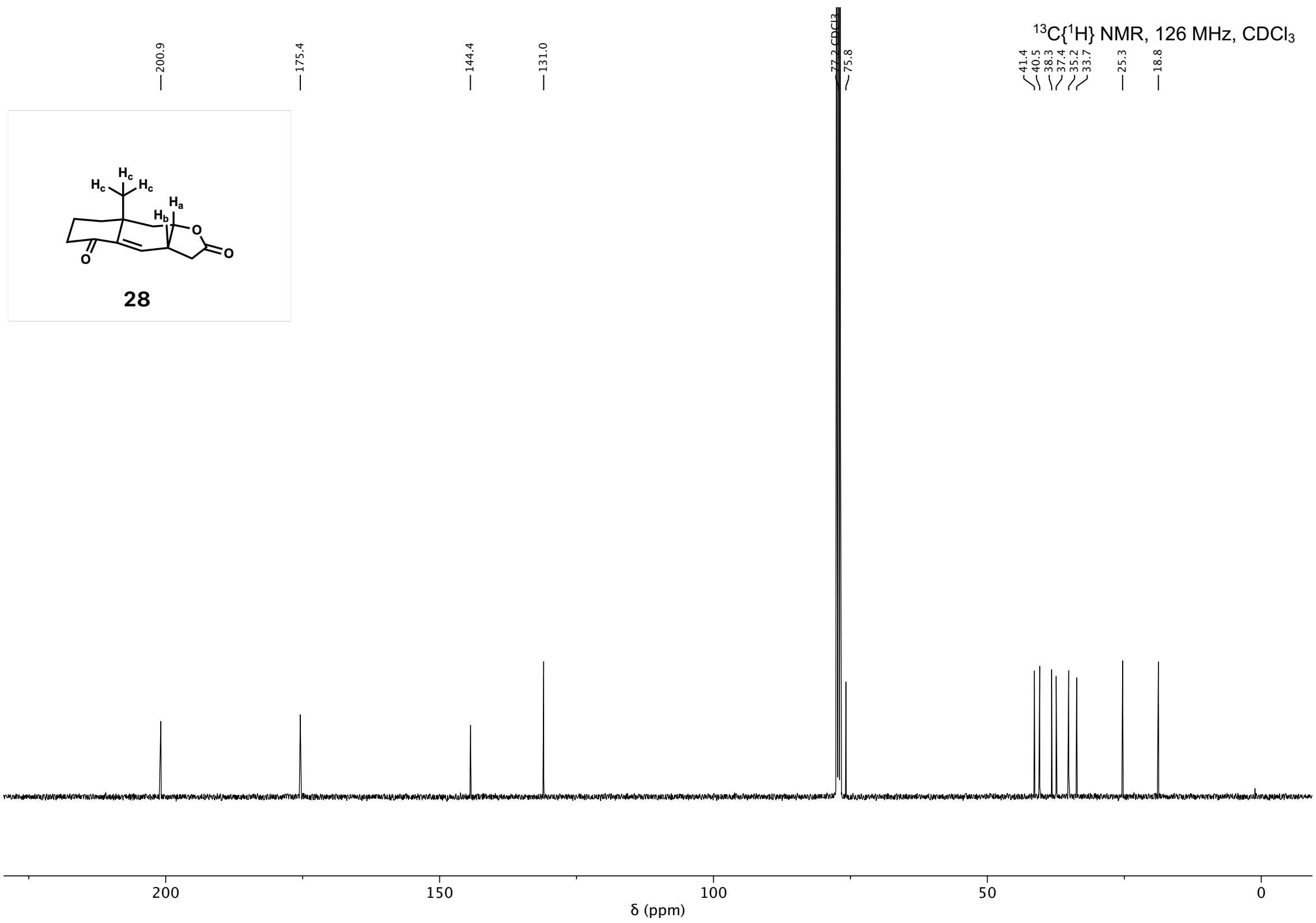

^{13}C{^{1}H} NMR, 126 MHz, CDCl$_3$
200.9
175.4
144.4
131.0
77.2 CDCl3
75.8
41.4
40.5
38.3
37.4
35.2
33.7
25.3
18.8
28
δ (ppm)

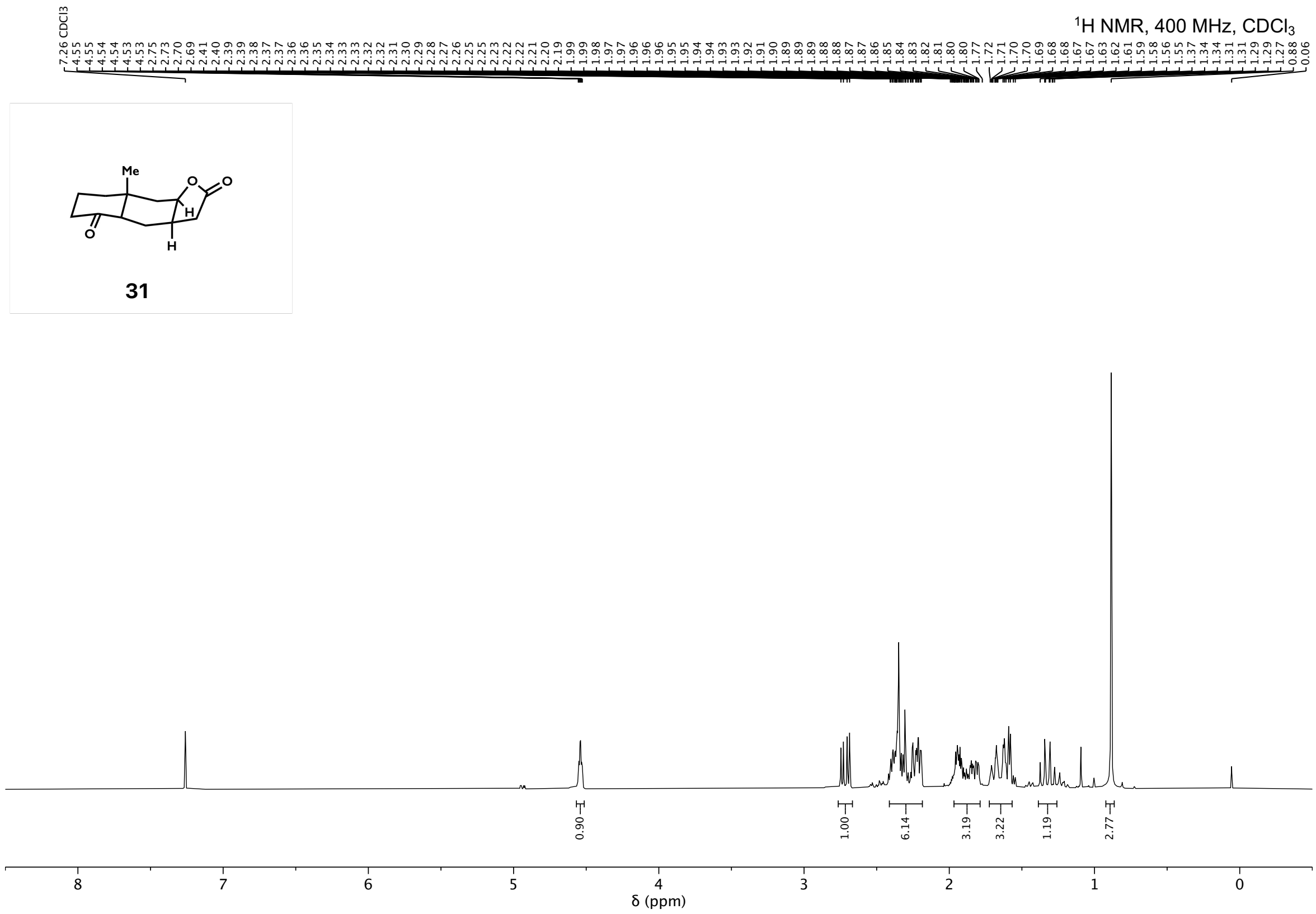
^{1}H NMR, 400 MHz, $CDCl_3$
31
δ (ppm)

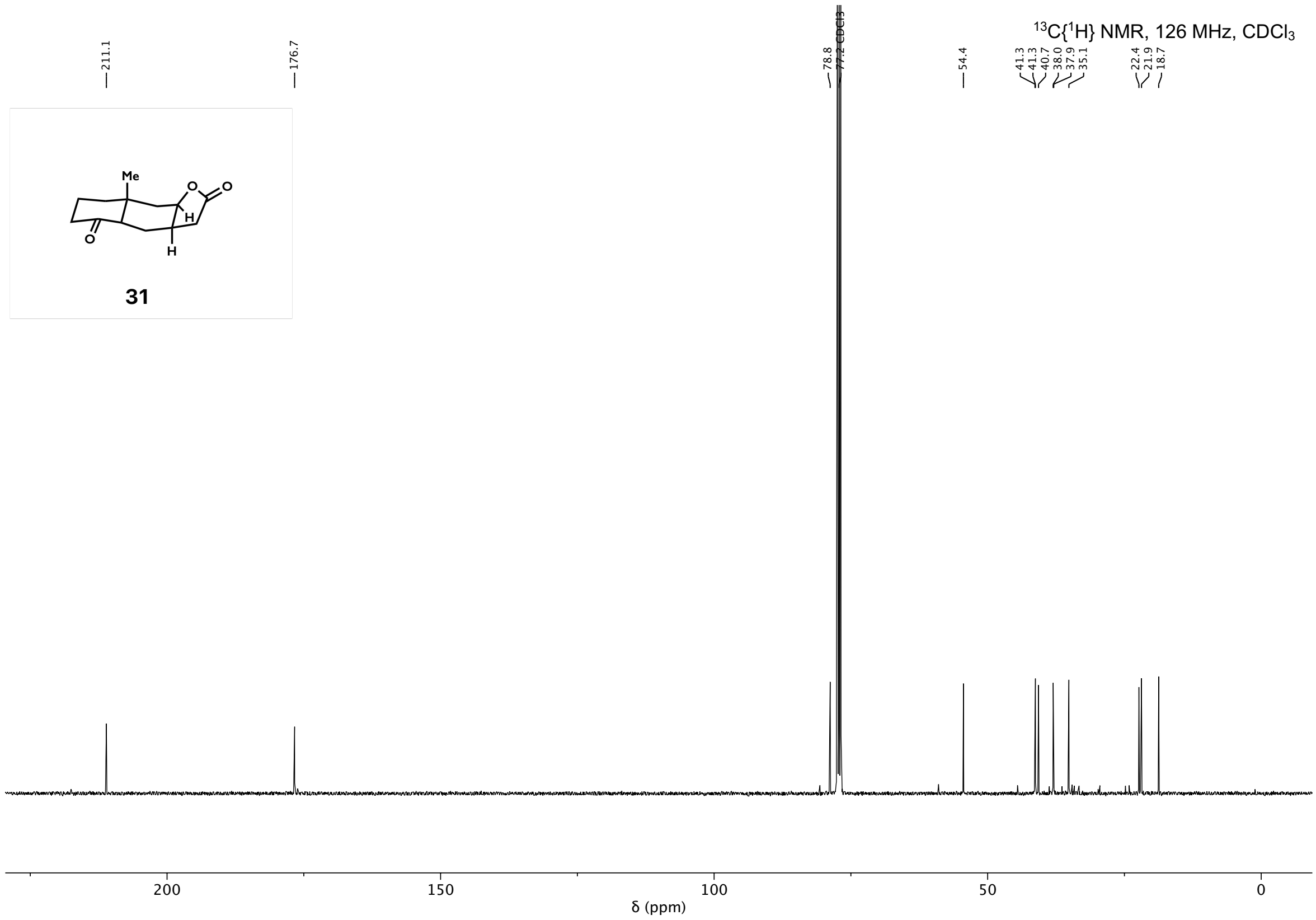
^{13}C{^{1}H} NMR, 126 MHz, $CDCl_3$
31
δ (ppm)

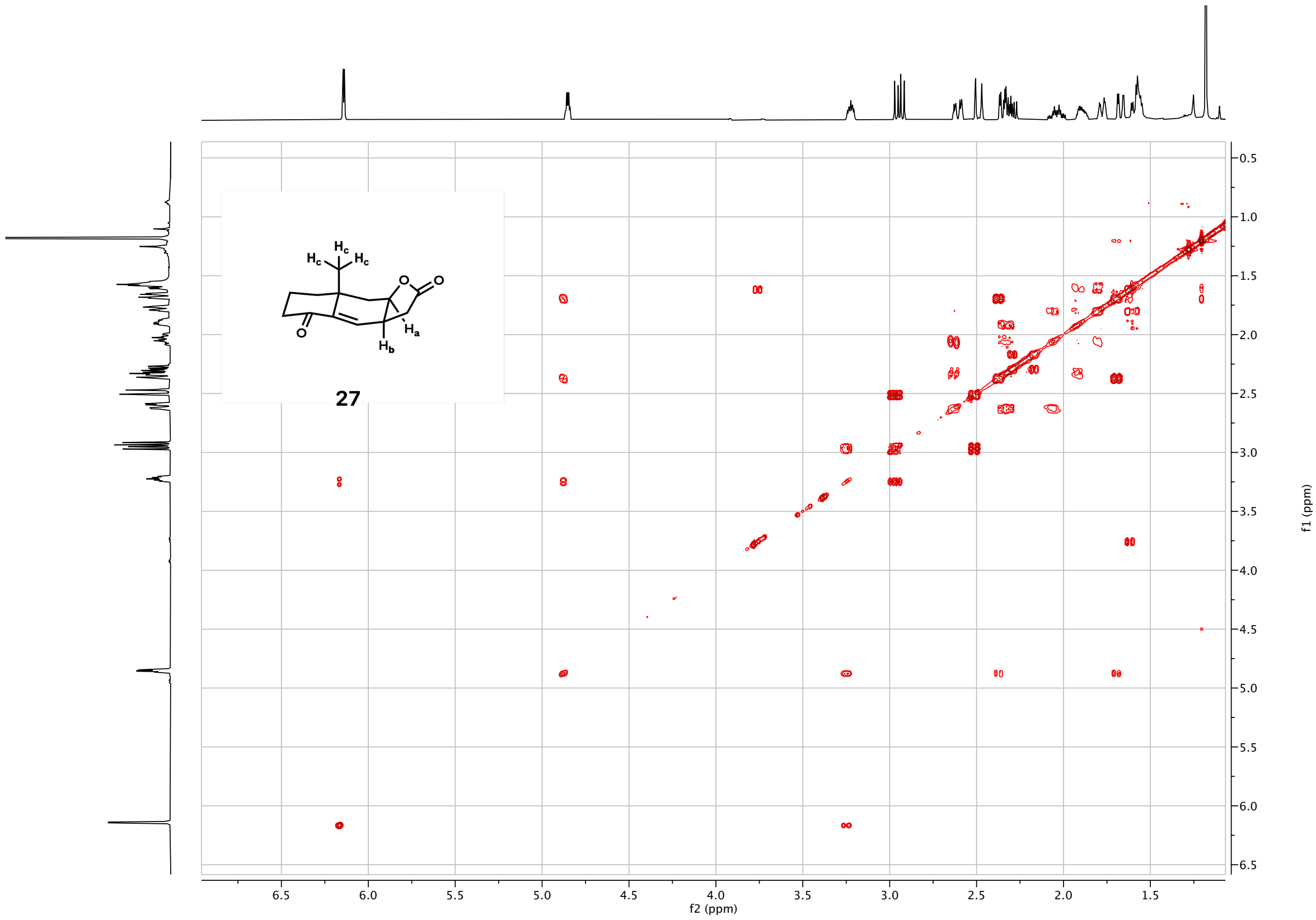

Figure S3. gCOSY NMR (^{1}H 500 MHz, $CDCl_3$) of compound **27**.

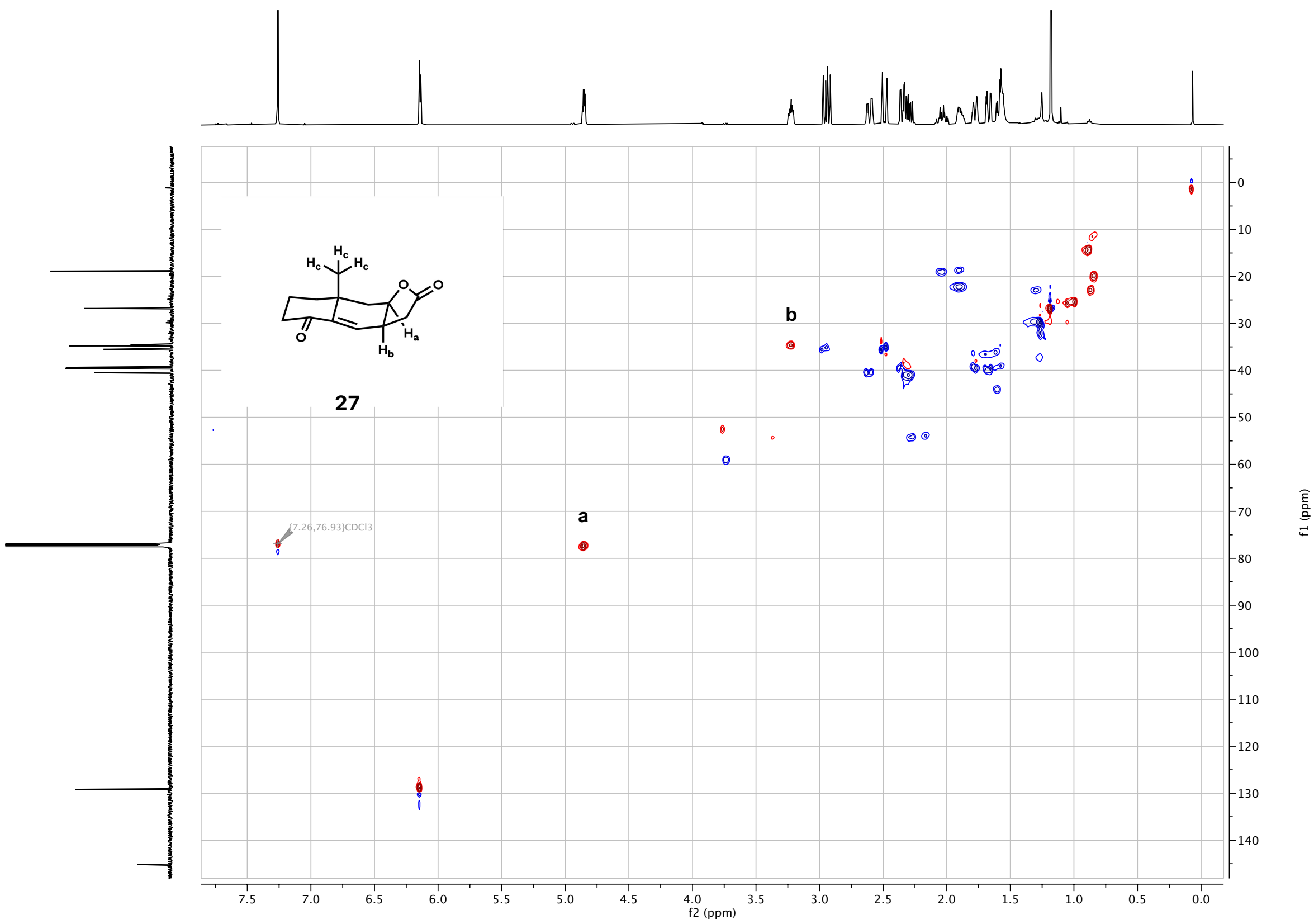

Figure S4. Edited HSQC NMR (^{1}H 500 MHz, $^{13}C\{^1H\}$ 126 MHz, $CDCl_3$) of compound **27**.

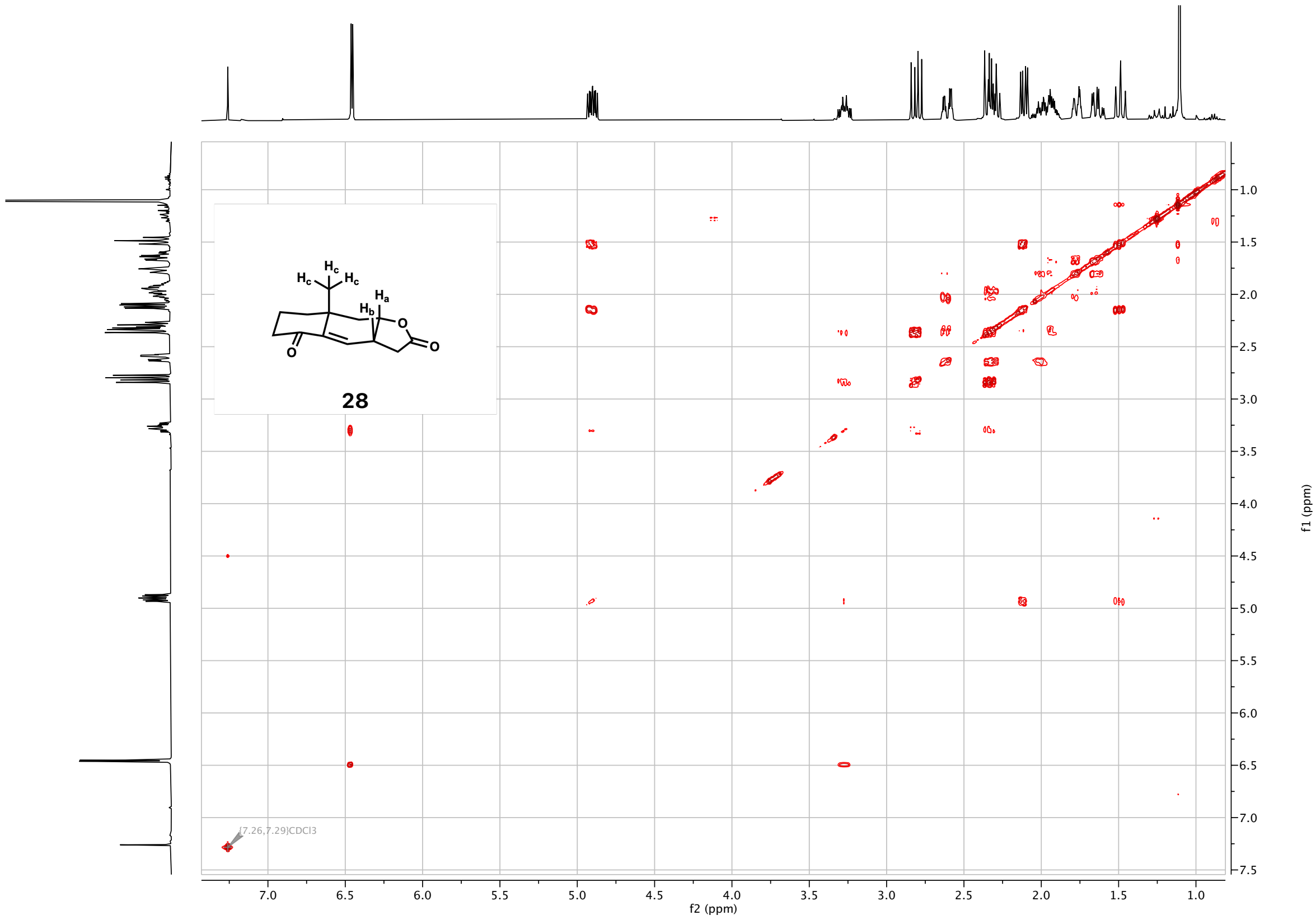

Figure S5. gCOSY NMR (^1H 500 MHz, $CDCl_3$) of compound **28**.

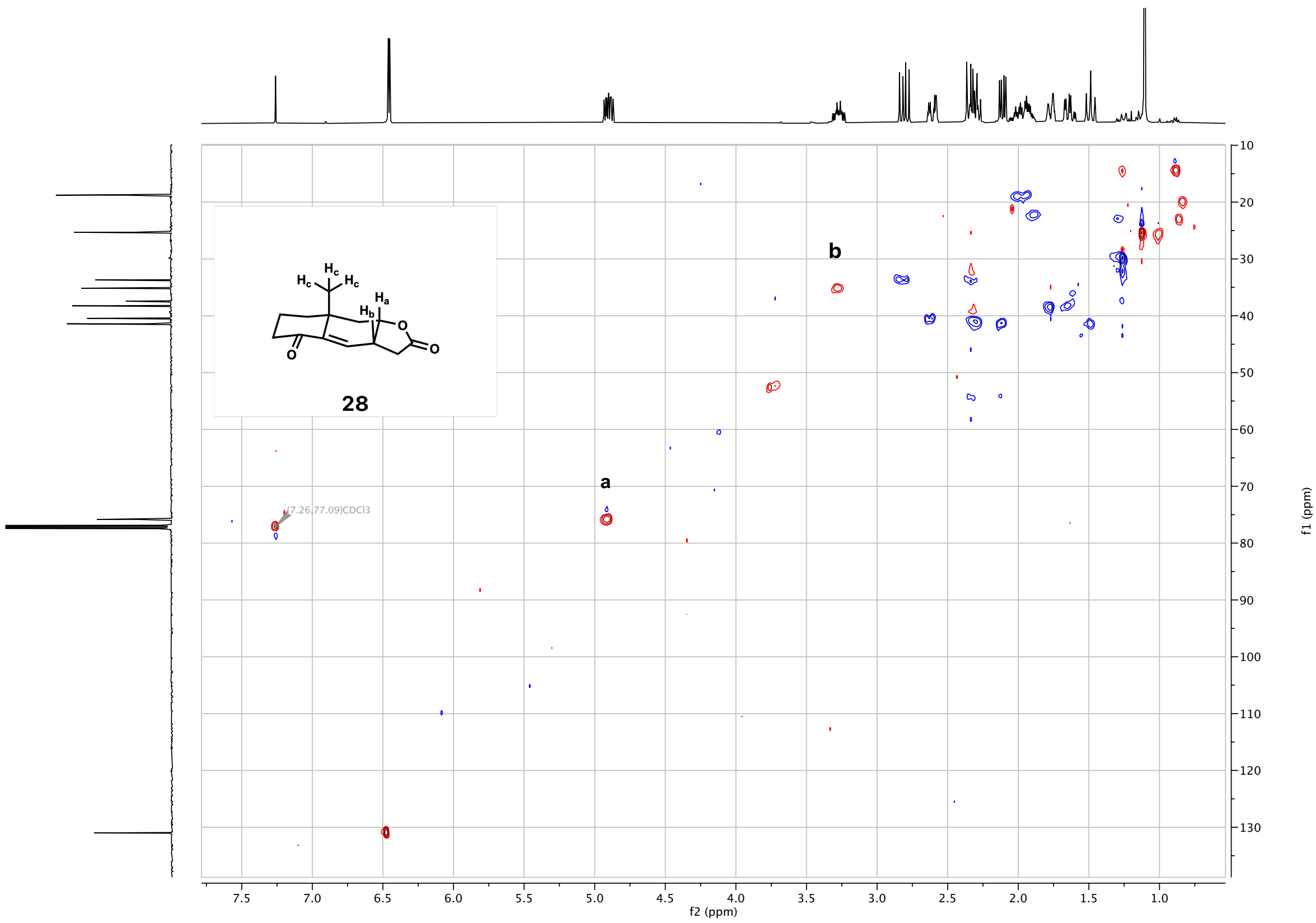

Figure S6. Edited HSQC NMR (^1H 500 MHz, $^{13}C\{^1H\}$ 126 MHz, $CDCl_3$) of compound **28**.

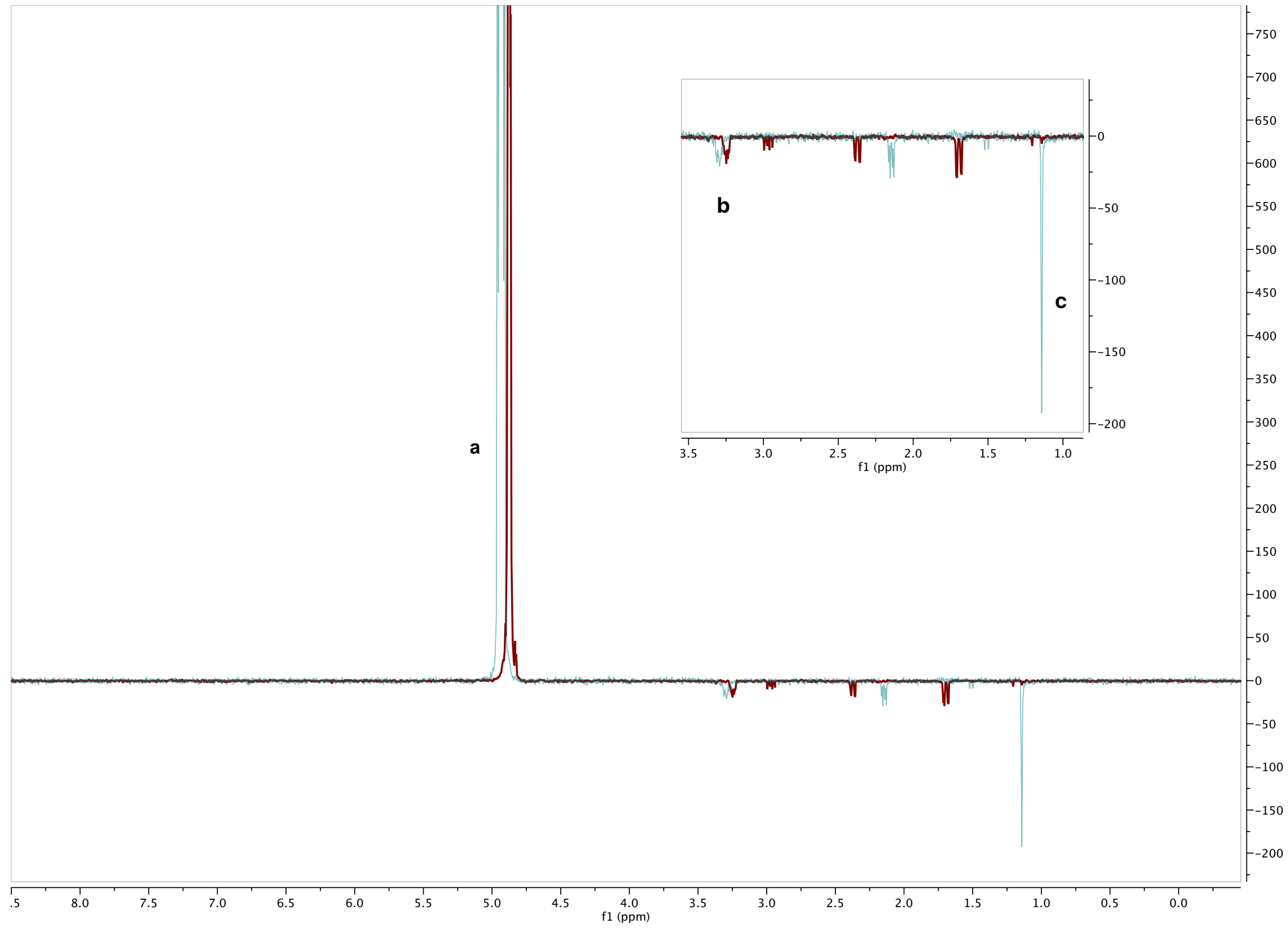

Figure S7. 1D NOESY (^1H 500 MHz, $CDCl_3$) with selective irradiation of H_a for compound **28** (**blue**) and **27** (**maroon**). Compound **28** shows NOE correlations to both the CH proton H_b and the CH_3 protons H_c, while compound **27** only shows NOE correlations to the CH proton H_b.

VI. Cartesian Coordinates of Computed Structures

27 (MeOH)

O	2.06572624566639	2.58336734278468	2.28213546574095
C	1.41315291391515	1.69018135374540	1.80850033075549
C	1.26585965163507	0.27000074804357	2.29572778257019
C	-0.00641907247786	-0.20874438750117	1.58706483243409
C	-0.00216652527559	0.65098564598744	0.31338181068350
O	0.69742100366031	1.85953595193428	0.69102486240291
H	0.60462180788721	0.16795814028883	-0.46643416036034
H	0.06460983005947	-1.27603319556861	1.33234922026019
C	-1.22715046126175	0.00903088314632	2.43211561095406
C	-2.28558877982103	0.75622531906363	2.08575950688004
C	-3.43873683735609	0.80901092810766	3.04524144170658
C	-4.82353727675450	0.94015207775604	2.45662884019755
C	-4.90342369288880	1.76947445425438	1.17566229750798
C	-3.80525157331331	1.36426920529092	0.19847016770968
C	-2.38649050410683	1.53041671660875	0.78152871350604
C	-1.37596178447343	0.97582901520784	-0.23601459392189
C	-2.12460457009144	3.01889911946656	1.07861190088393
O	-3.27486389066956	0.66062692957415	4.24206071442040
H	1.23893443341788	0.23275800097577	3.39158882077267
H	2.15303656712187	-0.28324798199027	1.94894539993462
H	-1.24604858568302	-0.50415436106073	3.39930611554083
H	-5.50178684985957	1.29557958652635	3.24439446921143
H	-5.11502929458292	-0.10169959653458	2.22557686692399
H	-4.83102939265369	2.84088867344521	1.41438773609241
H	-5.88921029523344	1.62090208279514	0.71233337117559
H	-3.87516226586985	1.95595810942288	-0.72832751417156
H	-3.94772458948436	0.30685206958712	-0.08314412183645
H	-1.27519032003883	1.67271306919472	-1.08279599919662
H	-1.76777568994194	0.02988198758358	-0.64427809130253
H	-2.82576319120755	3.40516363676499	1.83341541482952
H	-2.24806193690521	3.61578171753911	0.16084615980032
H	-1.10938507341275	3.17823675756003	1.46283662789442

27 (Toluene)

O	2.06205112324328	2.56709987043524	2.32245538877211
C	1.41617138261224	1.69032589606639	1.83312012361969
C	1.27174757440563	0.25226259207266	2.29178429704707
C	-0.00376471954923	-0.21288497995667	1.58129537409105
C	0.00603244899257	0.66778299128614	0.31974223972915
O	0.68822137788457	1.86855046137631	0.71336898724998
H	0.61465313924571	0.19073470538199	-0.46496412412371
H	0.05756793253095	-1.27792813420658	1.31241131496097
C	-1.22409241218537	0.00856952287513	2.42804399225627
C	-2.28129893360960	0.75656027608064	2.08371368850155
C	-3.43331772407242	0.81514082244779	3.05429924814245
C	-4.82327827979326	0.95335402688286	2.46231081178124
C	-4.90281737277378	1.76584590813843	1.17023168163915
C	-3.80512163588393	1.34710129259983	0.19747299122713
C	-2.38712666362277	1.52811947565208	0.77860799180784
C	-1.36919048903982	0.98396394427090	-0.23734961409499
C	-2.13926276700449	3.02083101855187	1.06915223972965

O	-3.26842936407615	0.66152269738550	4.24234932660074
H	1.24667029403926	0.19572441495579	3.38666725727196
H	2.15766288429326	-0.29664933935081	1.93576769612246
H	-1.24692830590694	-0.49064188930007	3.40231159837343
H	-5.49299821916750	1.32697724435951	3.24847189259851
H	-5.13104310465116	-0.08688122851198	2.24805573677322
H	-4.82505562796901	2.84039656998386	1.39250419849350
H	-5.88935914255510	1.61764998405722	0.70802180336885
H	-3.87770231915711	1.92321883015517	-0.73901941147330
H	-3.94513283267080	0.28474010475318	-0.06768667812043
H	-1.26750236043179	1.68772393067105	-1.07824300459670
H	-1.75542501984837	0.03898092247355	-0.65396928849756
H	-2.80842251638814	3.39303452747187	1.85885227290726
H	-2.31803225595305	3.61839444801774	0.16094528012433
H	-1.10847609093766	3.19717909292340	1.39817468771709

27 (THF)

O	2.06119462400791	2.56023091879286	2.33352662312908
C	1.41553780461813	1.68305387415463	1.83830442747726
C	1.27154081716792	0.24438056823563	2.28880581814703
C	-0.00683707228074	-0.21334616037077	1.57944592763204
C	0.00505426676230	0.66843902647870	0.32028163768753
O	0.69134515717223	1.86956977781072	0.72029589899471
H	0.61495406013086	0.19332601431149	-0.46366189814838
H	0.04944045269006	-1.27787746596589	1.30878636754324
C	-1.22377399420547	0.01323799415815	2.42938341153106
C	-2.28011110587230	0.76378954435828	2.08636556979924
C	-3.43100349357623	0.82802170388906	3.05643670003032
C	-4.82002014339743	0.94928289624637	2.46243958192580
C	-4.90388235672639	1.76097645318741	1.17016153722412
C	-3.80455115365273	1.34663906286301	0.19776902198059
C	-2.38725627960238	1.53163072500427	0.77881888486598
C	-1.36872589251942	0.98769371700670	-0.23639901354810
C	-2.14274097448060	3.02518265795050	1.06681418760582
O	-3.26394499213642	0.69387794923848	4.24821017111369
H	1.25117330620128	0.17780229804065	3.38329816089782
H	2.15387971916943	-0.30356122921939	1.92218166731586
H	-1.24285891456688	-0.48817987980224	3.40259649724757
H	-5.49757961022492	1.31593605736365	3.24530948734179
H	-5.11352125948985	-0.09460585805745	2.24521687374413
H	-4.83190815268582	2.83589981972901	1.39304780027897
H	-5.88962665198986	1.60568666247977	0.70863881245522
H	-3.87805942592706	1.92336274608840	-0.73825155683608
H	-3.94028599976978	0.28384166787865	-0.06705239253990
H	-1.26839231520312	1.69064076891332	-1.07814364273087
H	-1.75401111516837	0.04207247416025	-0.65200722204405
H	-2.81939916075129	3.39938661788813	1.84941555185000
H	-2.31437036075795	3.61887060462639	0.15465018290020
H	-1.11425978293509	3.20153799256125	1.40421492512826

27 (gas phase)

O	2.06494049526102	2.56580041817837	2.32675823996804
C	1.41797314678492	1.69533567149401	1.83758684014565
C	1.27268263424202	0.25175700668404	2.29259812655539
C	-0.00199226581576	-0.21260518385160	1.58024270852985

C	0.00803943451545	0.67457969140515	0.32196768766085
O	0.67887072691742	1.87332176870011	0.71855292886107
H	0.61942961950557	0.20024309588431	-0.46397279331529
H	0.05913424565458	-1.27731169317461	1.30709558732252
C	-1.22414526470510	0.00568630199534	2.42552291554167
C	-2.28055238405471	0.75297842921476	2.08011572139494
C	-3.43176197702725	0.80740224423588	3.05362955075861
C	-4.82438147363385	0.95923890649067	2.46425507721144
C	-4.90160068429423	1.76871727277530	1.16980757707870
C	-3.80528487147491	1.34332951822647	0.19757527043104
C	-2.38702792458488	1.52632405730042	0.77732692608455
C	-1.36855632073940	0.98324086900654	-0.23832805701378
C	-2.13976564490493	3.02020349278774	1.06652985662972
O	-3.26747568385767	0.63725350167090	4.23701033233495
H	1.24482227469535	0.19707874964112	3.38739075853724
H	2.16000513641248	-0.29835503657397	1.94259857044072
H	-1.25248185372514	-0.48800170944779	3.40256464620755
H	-5.48537270642827	1.33998700633473	3.25405912200244
H	-5.14535083128999	-0.07794919819242	2.25569864751206
H	-4.81894257844864	2.84373069027700	1.38750837423213
H	-5.88893327261849	1.62586515122711	0.70767051743492
H	-3.87879252905639	1.91364297310496	-0.74245329151892
H	-3.94689788990442	0.27942403799564	-0.06191254645978
H	-1.26697111259762	1.68687158038079	-1.07931393139720
H	-1.75370008594338	0.03690269236380	-0.65380725445342
H	-2.80021131675931	3.38749464253231	1.86518694219524
H	-2.33364301785223	3.61597025553658	0.16052563223737
H	-1.10505602427219	3.19864279579628	1.37890931684972

28 (MeOH)

O	4.96216030654571	-0.62348629889296	-1.50330560021489
C	3.95387897982673	-0.07605311002104	-1.14293766084260
C	3.01547898793300	-0.47813919300826	-0.03105652464618
C	2.17903109601926	0.78912384132836	0.19219197638339
C	2.25915480492119	1.48410415871159	-1.17746940735296
O	3.50692060811391	1.03592114157891	-1.74277456240766
H	2.33932557283979	2.57324483098465	-1.07877405618056
H	2.69331731376709	1.41096808764261	0.94174430760280
C	0.77933360877854	0.54095784887312	0.65395943022021
C	-0.31462853358010	0.82486100711039	-0.07008814785297
C	-1.65684897172413	0.50457353934478	0.51599714757371
C	-2.89598611792615	0.89267869491472	-0.26011036771142
C	-2.76143717673692	1.00826929297057	-1.79161079525828
C	-1.35212311265223	0.70858057828653	-2.29983938043774
C	-0.25884751013467	1.40655101394388	-1.47345470537165
C	1.11088385708219	1.09202461840410	-2.09195564534079
C	-0.49659674978692	2.92494584877767	-1.43114737286110
O	-1.76309970984070	-0.02125785423956	1.61008182370907
H	3.58303310239324	-0.82157925079807	0.84179257757731
H	2.40775878207308	-1.31939015421565	-0.40166781816092
H	0.65199489790747	0.09562627490334	1.64508319307439
H	-3.18694696767843	1.86564208020915	0.17402001469734
H	-3.68427365227177	0.18915780784148	0.04102628180122
H	-3.05564964775972	2.01849579507341	-2.10879716824909
H	-3.46832670224709	0.31899522158629	-2.27371217864798
H	-1.26264020518685	1.01354687951550	-3.35459905758430

H	-1.16280260638143	-0.37789990080730	-2.26295098627731
H	1.21565273591416	1.60679831304115	-3.05933401550190
H	1.17721974156056	0.00694954374649	-2.28151797639449
H	-0.56348439781072	3.32903706733848	-2.45363070382477
H	0.32110696436480	3.44092363626634	-0.90752503697409
H	-1.42705929832289	3.17752863958931	-0.90153758454575

28 (Toluene)

O	4.96882932569453	-0.60159959738748	-1.50678817083160
C	3.96607583355310	-0.06108149455071	-1.14977074410147
C	3.01663724276773	-0.48124136062029	-0.04250045188343
C	2.17816211038170	0.78040861332924	0.19928755298979
C	2.26189489723045	1.48821467076256	-1.16563141922798
O	3.50890059408062	1.06650020934863	-1.72868134672220
H	2.32008044721198	2.57842809695671	-1.05340938707036
H	2.69511611205090	1.39563023339961	0.95326735316338
C	0.77606989574391	0.53005422329631	0.65681852867140
C	-0.31551851574953	0.81695241368587	-0.06765167426166
C	-1.66293098263491	0.48104480672062	0.51473720906444
C	-2.88990970116410	0.93751080779508	-0.25215326272717
C	-2.76509360820111	0.98661404918657	-1.78984793044019
C	-1.34980787357018	0.70085609385786	-2.29640975272515
C	-0.25941204174215	1.40411628437057	-1.46967962182083
C	1.11434638746359	1.09632759247774	-2.08386247648283
C	-0.50338689110114	2.92199919018574	-1.43565184370331
O	-1.77030324481105	-0.09216683820318	1.57525048114969
H	3.58255010845810	-0.83893852684035	0.82558072696861
H	2.40815890723561	-1.31756947805308	-0.42196226578096
H	0.63237515324477	0.07504780268586	1.64163001925308
H	-3.09573943915774	1.94741203367303	0.14380589143405
H	-3.72511411228695	0.30915820167729	0.08345961606425
H	-3.08969497289857	1.97331416908502	-2.14966251494034
H	-3.45455895062898	0.25832556833948	-2.23882148041229
H	-1.26220036918368	1.00257869118462	-3.35245108941354
H	-1.15160363168165	-0.38390419460201	-2.25934496056122
H	1.22399534057062	1.61043242337947	-3.05121763051543
H	1.18306751544923	0.01226862438445	-2.27907318121296
H	-0.55252111269412	3.32508460590260	-2.45962528550642
H	0.30099972993816	3.44437968206379	-0.89806179437609
H	-1.44396415356914	3.17554240250837	-0.92547909404123

28 (THF)

O	4.96272553091850	-0.61358188436198	-1.51080086585334
C	3.96052362457580	-0.06815627668880	-1.15149848890455
C	3.01150883136004	-0.48386309620688	-0.04488450589836
C	2.18053412802071	0.78279780617614	0.19690624449614
C	2.26319846879510	1.48827151638111	-1.16809420360809
O	3.51122849779702	1.05930918364738	-1.73203115056504
H	2.32614349350656	2.57811418583083	-1.05810335865676
H	2.70172125910999	1.39568273464268	0.94952678811152
C	0.77906234121879	0.53934212789971	0.65822935141908
C	-0.31379445226724	0.82505758636826	-0.06543218684242
C	-1.66015857948488	0.49893255697193	0.52185425521576
C	-2.89460055872023	0.91280314039216	-0.25684350272981
C	-2.76039672430247	0.99670687400719	-1.79134741174608

C	-1.34752836434757	0.70074564605653	-2.29479865285387
C	-0.25745674040413	1.40548087094227	-1.46944828407231
C	1.11502072665150	1.09632289974557	-2.08501586677977
C	-0.50149117377401	2.92337049664606	-1.43549480897528
O	-1.76624542409217	-0.04072420250343	1.60207173794656
H	3.57402060601284	-0.84420519556425	0.82425661130606
H	2.39758412573543	-1.31513497400194	-0.42696685789013
H	0.64039029934661	0.09216147011471	1.64710462491717
H	-3.15457818561352	1.90273026709382	0.15716792654910
H	-3.70263917058364	0.23892899799249	0.05752671768951
H	-3.06424060541518	1.99725841207839	-2.12990887294978
H	-3.45948211612094	0.29071330779465	-2.26060089366202
H	-1.25691327084920	0.99987489118428	-3.35126549440525
H	-1.15390570551480	-0.38468519141416	-2.25211024138569
H	1.22106743271047	1.61227739832544	-3.05169624515828
H	1.18276212765464	0.01195938900771	-2.27874561296545
H	-0.56514661360160	3.32215195798042	-2.46029346593961
H	0.31181923681574	3.44526204545151	-0.91113092478657
H	-1.43523304513818	3.17579505801019	-0.91203236102240

28 (gas phase)

O	4.84197615940124	-0.84381325928572	-1.46462004535943
C	3.88300268339589	-0.22026543249335	-1.13700884756737
C	2.92129529197515	-0.50708775065119	0.00793537327713
C	2.17024368508994	0.81920573583931	0.17969179294123
C	2.27804721267653	1.43018886128533	-1.23047997049515
O	3.48082295693522	0.89815056158836	-1.78197538560229
H	2.40454852571535	2.52001802043598	-1.18885437773715
H	2.74183439159400	1.44729422649575	0.88248532669358
C	0.76343937504186	0.70142087070619	0.67861696558824
C	-0.32517106731202	0.97329593157427	-0.05543846340016
C	-1.66807299289659	0.77320120303087	0.60060326788736
C	-2.84217974157088	0.45384884233371	-0.30786005195536
C	-2.77097445291939	1.08791296591428	-1.69595152565944
C	-1.39672319913950	0.86444983605971	-2.32009197668653
C	-0.25287076729686	1.47741735224033	-1.49016615868124
C	1.08977731427681	1.05775585012467	-2.10543077836585
C	-0.38125879095907	3.01202179388622	-1.47791098662437
O	-1.78945365443101	0.78067435966899	1.80175147269993
H	3.47596613161757	-0.85038367321095	0.88871418440683
H	2.25051840413787	-1.32162649951679	-0.30763530672985
H	0.61068661285487	0.36669736527286	1.70913117719367
H	-3.76434920350553	0.69974719441800	0.23498493222648
H	-2.82104819718609	-0.64605588250850	-0.41596079356262
H	-2.99171013284115	2.16431096499980	-1.63437572071074
H	-3.54995647244095	0.65293991070164	-2.33818246380759
H	-1.35848557670716	1.28408807071237	-3.33799506918176
H	-1.21853781299270	-0.22061949296855	-2.41706202426627
H	1.21458331922625	1.50687681071525	-3.10292137932130
H	1.09289355884357	-0.03719446791341	-2.24147707427975
H	-0.38212331433837	3.40595733601803	-2.50623839657805
H	0.44744643900590	3.47600344317502	-0.92568181099527
H	-1.30866668525074	3.33526895135149	-0.98449588534686

29 (MeOH)

O	-3.83145011098964	-0.82956082395685	-0.37020845782211
C	-2.77364000779097	-0.40594800853253	0.00910995741791
C	-2.48892404255360	0.85941543907283	0.80338024110021
C	-1.01493517679243	1.05831370814274	0.47403548349352
C	-0.54937206272042	-0.38421814083545	0.43572135930675
O	-1.61964701034303	-1.05420241377630	-0.25471610368239
H	-0.53424353848258	-0.78633809735255	1.46235619171779
H	-0.96784095805357	1.44315747986502	-0.56190809032108
C	-0.06291594445429	1.86539193631538	1.29473242037572
C	1.22920090560208	1.49989745980593	1.36202776184618
C	2.17907225208938	2.43145051026846	2.05421727964506
C	3.55177357777780	2.58756795471311	1.44067770155408
C	4.12590660056444	1.33189784643970	0.78332619141491
C	3.07590534161532	0.62078615712229	-0.06475221841182
C	1.82546652994495	0.22969948516425	0.74637448335419
C	0.81993639107841	-0.49979650760167	-0.18132701862387
C	2.22650728870204	-0.70836349339664	1.90076167457749
O	1.82826885499834	3.11020503869698	3.00117076214956
H	-2.64878478037989	0.63269473855444	1.87063770411994
H	-3.17068411644442	1.66190956748499	0.49962543950051
H	-0.38807552922230	2.79882580166109	1.76298932741198
H	4.22136871716484	3.02187758167081	2.19567081937696
H	3.40117728441211	3.35925935925784	0.66245005777516
H	4.51626158587435	0.64959312892701	1.55287189539735
H	4.98271089283893	1.61609032052541	0.15612840968086
H	3.50015836636907	-0.29085802097846	-0.51544292480611
H	2.76489482055890	1.27791255503337	-0.89466410321845
H	1.12784483674579	-1.54989690701648	-0.29574027700056
H	0.80848414608082	-0.03250646294722	-1.17876852158932
H	1.34004001997885	-1.12201864498651	2.40280132601968
H	2.81674523547465	-0.18661550488969	2.66909636595522
H	2.82928963035600	-1.54602304245133	1.51646486228463

29 (Toluene)

O	-3.83317225570054	-0.83222147730358	-0.36904275381583
C	-2.77935968084410	-0.41593464226435	0.00348731103300
C	-2.49128328480746	0.85449336183584	0.80464747081106
C	-1.01885980930278	1.05993372903728	0.47317260139032
C	-0.55549716873521	-0.38480393730885	0.42815995619926
O	-1.61325765771154	-1.05500998316477	-0.26115656563992
H	-0.53220122157997	-0.77999038839076	1.45894700968950
H	-0.97613964936710	1.44451562280675	-0.56332333464576
C	-0.06309524840669	1.86613540024826	1.29297216263555
C	1.22874028538373	1.50315747283304	1.36041379598867
C	2.17935863228418	2.43717256658578	2.06115635835908
C	3.55526679831975	2.59056888191401	1.43822389402731
C	4.12808838148455	1.33113565491455	0.78627864536797
C	3.07825069358132	0.61959407569512	-0.06279552128195
C	1.82412571790189	0.23252848988437	0.74543501958897
C	0.81876318635742	-0.49640578936918	-0.18244609575438
C	2.22356475438441	-0.70622736491023	1.90047077802999
O	1.83167863057288	3.11167701745593	3.00231709699118
H	-2.64651291245105	0.63107430946139	1.87304354007018
H	-3.17704881933958	1.65503212356384	0.50536730965988
H	-0.38040613747780	2.79594428039050	1.77327019966157
H	4.22529799105205	3.02613277435565	2.19157382056521

H	3.40899258415118	3.35951279641018	0.65719222012493
H	4.51358800639978	0.64850612207654	1.55812638395851
H	4.98927647887905	1.60686254592545	0.16103772065962
H	3.50196974143599	-0.29279556218365	-0.51286187073919
H	2.77098950130889	1.27571847204532	-0.89508066629176
H	1.12521523622895	-1.54658222104400	-0.30109494924134
H	0.80887920624590	-0.03060970314840	-1.18084159784212
H	1.33731734079733	-1.12070132472494	2.40145223884238
H	2.80991941244889	-0.18448360121413	2.67129972628318
H	2.82805126650564	-1.54432970241296	1.51969809531489

29 (THF)

O	-3.83380092704504	-0.82829397179887	-0.36963210878444
C	-2.77815487063332	-0.41120956849488	0.00443710283003
C	-2.48922604407324	0.85833608510861	0.80255124791025
C	-1.01630928096652	1.05948097179954	0.47210482466491
C	-0.55412531781567	-0.38511912137186	0.43119541686330
O	-1.61668541721601	-1.05533575799416	-0.25809565386720
H	-0.53341862648055	-0.78049894855225	1.46149630754325
H	-0.97034463914095	1.44374070794333	-0.56424966899298
C	-0.06229761157760	1.86552688213308	1.29329216807695
C	1.22934111769144	1.50115675294287	1.36331265119781
C	2.18076299415270	2.43382533886088	2.06275214798077
C	3.55232288366977	2.59298284169543	1.43482467007661
C	4.12577324361545	1.33318849825438	0.78430307201689
C	3.07654603077118	0.61894077931986	-0.06223006053569
C	1.82429287876695	0.23033396417862	0.74766166354837
C	0.81902587822872	-0.49888808059204	-0.18040343585478
C	2.22437136016257	-0.70908579331464	1.90159868256081
O	1.83572893510416	3.10290757556058	3.01092325283965
H	-2.64500678161719	0.63436518465992	1.87091314331415
H	-3.17138291346844	1.66151842980521	0.50186939645598
H	-0.38310675141364	2.79678016208266	1.76853128690994
H	4.22511799532279	3.03227671053973	2.18362721338123
H	3.39808014897907	3.35818918550466	0.65146192121037
H	4.51356060029575	0.65378853438952	1.55801300611418
H	4.98571215681657	1.60974151601752	0.15757924678660
H	3.50129977603680	-0.29378649642329	-0.51057199935647
H	2.76630596115482	1.27365028071306	-0.89444689783178
H	1.12750000992178	-1.54879561284213	-0.29616311630023
H	0.80999558809600	-0.03219116987500	-1.17833780346826
H	1.33723148982111	-1.12171194864234	2.40304656364594
H	2.81500649318602	-0.18884871751484	2.67043193043176
H	2.82638363965445	-1.54736521409321	1.51730382863204

29 (gas phase)

O	-3.83409771934029	-0.83813456493244	-0.36352277776699
C	-2.78254742877926	-0.42371858537761	0.00476573532429
C	-2.49232679331256	0.85009964804276	0.80886009575040
C	-1.02134467122158	1.05993269490974	0.47393029311842
C	-0.55753595606119	-0.38528113781666	0.42422180567834
O	-1.60726999562246	-1.05638602349527	-0.26345835372441
H	-0.52899099798260	-0.77728464563640	1.45720054975659
H	-0.98310665090840	1.44490067177564	-0.56290656256717
C	-0.06325642735443	1.86729183843421	1.29112657963256

C	1.22822951164195	1.50512886917212	1.35567150975988
C	2.17715420582662	2.44183329589792	2.05704453202129
C	3.56007301552537	2.58807606769805	1.44124582080513
C	4.13026296315371	1.32760536856907	0.78834963047447
C	3.07925447702731	0.61983037174825	-0.06309245626447
C	1.82336871919234	0.23448394283183	0.74288985593423
C	0.81817574079487	-0.49353189183025	-0.18489612137125
C	2.22117211271031	-0.70352416958811	1.89986187360773
O	1.82542035234354	3.12448733833128	2.98760949110503
H	-2.64360975718936	0.63055488483236	1.87841629272463
H	-3.18317401101194	1.64710918842082	0.51241460063123
H	-0.37429968656249	2.79545614530148	1.77847213140371
H	4.22590508413015	3.01955997270930	2.20029450890809
H	3.42486912707301	3.36143785826903	0.66282618244738
H	4.51344833519171	0.64184905822997	1.55860080081674
H	4.99465403974708	1.59908013675556	0.16574083581625
H	3.50129557399791	-0.29155086253220	-0.51693679888068
H	2.77337237582583	1.27853040384403	-0.89409337439886
H	1.12258528478891	-1.54365270012763	-0.30894164314238
H	0.80685221173602	-0.02779278474629	-1.18344976538162
H	1.33446193965880	-1.11516596909023	2.40093168445885
H	2.80602551103668	-0.17958680463719	2.66967341358995
H	2.82547951394439	-1.54203761596317	1.52024962973261

30 (MeOH)

O	-3.12500347393163	0.99680052193911	-0.27567923653491
C	-1.99164485077480	0.64650440406244	-0.35077937883902
C	-1.42577240807616	-0.59832325243869	-1.04396636970076
C	0.02814574799831	-0.18614630341273	-1.22810963671755
C	0.23965662348111	0.61502196382090	0.04909174906839
O	-0.96551016291276	1.35475082732192	0.19601940251253
H	0.31673931559787	-0.09025695960805	0.89880914400322
H	0.06450846744963	0.53177507023130	-2.06879685240250
C	1.15757073753150	-1.15360135369915	-1.37920976356192
C	2.36110960994723	-0.84963578537069	-0.86684967596409
C	3.44478501181784	-1.89265905586216	-0.95104729071623
C	4.45671435825301	-1.85172157949543	0.18228258755163
C	3.79350399589391	-1.30499552840517	1.44578432922286
C	3.26071392496087	0.12535825241302	1.27436928088763
C	2.71708285771234	0.45475831262647	-0.14893782008074
C	1.50593755553218	1.42413992760140	-0.04041509187673
C	3.80365535152324	1.17571936305064	-0.97008273540471
O	3.45784846197915	-2.74290822076812	-1.80613640212120
H	-1.99533287753565	-0.81586122425899	-1.95419894230078
H	-1.52422321947786	-1.45341261784514	-0.35492500623479
H	1.03365867628713	-2.10863292834310	-1.89769229557886
H	5.31477239694976	-1.22856742304925	-0.11909681429489
H	4.84009619415907	-2.87255062641167	0.31371873548976
H	4.48884093437375	-1.34554783444470	2.29584125505860
H	2.95487839595397	-1.97238721072375	1.70191643822417
H	4.05059012619817	0.85712907039011	1.50917370590799
H	2.46447220505700	0.28829888260302	2.01713654500877
H	1.44754598767143	2.07107589863811	-0.92970332969661
H	1.63628566410162	2.07879736385519	0.83415347957203
H	4.73988720145019	0.60357222895672	-1.02221819351430
H	4.03037975639345	2.14876949621126	-0.50751628919041

H	3.45580743443504	1.35413632041523	-1.99813552777661

30 (Toluene)

O	-3.12276293110868	0.98962892343504	-0.26108161795089
C	-1.98579642214726	0.63925068032447	-0.34320723706262
C	-1.42469950808116	-0.60394799364805	-1.03320791136928
C	0.02782378732247	-0.18956964095776	-1.22424159422307
C	0.24685783003512	0.61633440424139	0.04837762012804
O	-0.96739277633642	1.35235040754659	0.19806487402692
H	0.32672371457896	-0.08094044812963	0.90334495618970
H	0.05783868747302	0.52488195019709	-2.06790002785486
C	1.15749865206527	-1.15518180483753	-1.37791950726050
C	2.36354649877344	-0.84615569633471	-0.87417386066064
C	3.45125250628899	-1.88254172417003	-0.95757099781407
C	4.45009016131735	-1.84979877434124	0.18451462207913
C	3.77984211840829	-1.30518197308614	1.44519040090909
C	3.24875981383410	0.12544426996978	1.27304181455979
C	2.71892152561779	0.45823115540752	-0.15500954183978
C	1.50889927070507	1.42993136663788	-0.05411648821109
C	3.81437472224480	1.17275723061914	-0.96743840332558
O	3.48012994976506	-2.72174450888241	-1.82685368230486
H	-1.99496465913487	-0.82113824775776	-1.94339859789978
H	-1.52300443336650	-1.45528867339844	-0.33947140131610
H	1.02533669313987	-2.11077193830543	-1.89361509779907
H	5.31047919459104	-1.22657378574237	-0.10987226062599
H	4.82901018872560	-2.87214124208378	0.31882405933114
H	4.47466819993653	-1.34641346963455	2.29560968364430
H	2.94139050398097	-1.97424924351636	1.69678208101708
H	4.03642418243785	0.85596671216062	1.51765697649299
H	2.44244003964517	0.28516802263535	2.00516977922605
H	1.44758996372367	2.06565398335801	-0.95116063138981
H	1.64539463055340	2.09095742280824	0.81469898954738
H	4.74755829902557	0.59436364434230	-1.01429768960487
H	4.04674004463147	2.14401295014548	-0.50361366883077
H	3.47672955135396	1.35610604099732	-1.99832563980799

30 (THF)

O	-3.12179366747098	0.98543370096823	-0.25559406064034
C	-1.98264464300187	0.63454001881886	-0.34004157395740
C	-1.42217276284104	-0.60795831159868	-1.02696726448456
C	0.02844749664399	-0.19000890668155	-1.22260164438389
C	0.25088974861915	0.61672623672884	0.04803327282021
O	-0.96826068380423	1.35121632163997	0.19903026800439
H	0.33172173556004	-0.07755784790305	0.90471656146021
H	0.05543525728144	0.52229284842120	-2.06812275146604
C	1.15819172625576	-1.15470929753575	-1.37875534313473
C	2.36561423861928	-0.84375086238387	-0.87860562841579
C	3.45518509813689	-1.87673739412350	-0.96220122723036
C	4.44507224355392	-1.85052169313529	0.18614750578561
C	3.76898006899841	-1.30738783919480	1.44412873015617
C	3.24551726591555	0.12556726188493	1.27167141324393
C	2.72002540121099	0.46004991815093	-0.15754857622270
C	1.51068149214505	1.43239948259166	-0.05941855954066
C	3.81808144601062	1.17417854773373	-0.96635596130121
O	3.49385468210653	-2.70866684154639	-1.84035388237309

H	-1.99263822123619	-0.83019889814121	-1.93590363239810
H	-1.51555080293591	-1.45594349570067	-0.32820939390591
H	1.02056180892817	-2.11065537462414	-1.89243076341872
H	5.30732359021688	-1.22652837253228	-0.10192038719532
H	4.82206042519904	-2.87371770459280	0.32101938398644
H	4.46000659006953	-1.35289226677311	2.29744160644144
H	2.92612606874456	-1.97354932822041	1.68891670943530
H	4.03720443673486	0.85171541798912	1.51597862354721
H	2.43810123050556	0.28904692454076	2.00165123162296
H	1.44902565560473	2.06332787916641	-0.95983674111956
H	1.65057218092240	2.09491058956708	0.80775797216383
H	4.75061357641008	0.59412395888657	-1.01027337119442
H	4.04948177568287	2.14406824077177	-0.49911233768811
H	3.48198554121386	1.36058708682743	-1.99744017859678

30 (gas phase)

O	-3.12438663919261	0.99132069773562	-0.26417088216069
C	-1.99050209057893	0.64370507898377	-0.34368818601507
C	-1.42538204576243	-0.59976092957394	-1.04046362063200
C	0.02809402501701	-0.18677749358665	-1.22721114040723
C	0.24257466653004	0.61655066091137	0.04883628285651
O	-0.96382502252417	1.35306617972033	0.20033612268571
H	0.32544596105133	-0.08765154701188	0.89925183350842
H	0.06036952276563	0.53102491027481	-2.06825122054816
C	1.15812786616726	-1.15276867600152	-1.38032153973183
C	2.36127401510910	-0.84759279230238	-0.86911243145617
C	3.44553274651478	-1.88953552146473	-0.95232143102246
C	4.45657870644260	-1.84722298887449	0.18188626429508
C	3.78968500779270	-1.30347324921843	1.44542478736549
C	3.25147972688817	0.12539626045392	1.27517966637864
C	2.71746732546170	0.45648722166816	-0.15136488132965
C	1.50751503080800	1.42737819160752	-0.04810263972032
C	3.81050138615090	1.17126682776450	-0.96861101709557
O	3.45829488422416	-2.74120459649740	-1.80630806457033
H	-1.99638826914833	-0.81305572111832	-1.95089584425059
H	-1.52380338418403	-1.45696339017612	-0.35424661996597
H	1.03659998854318	-2.10716472967246	-1.90080482748333
H	5.31335379870998	-1.22187320899289	-0.11753195585537
H	4.83979184273287	-2.86785029075686	0.31393552910463
H	4.48526222750916	-1.34261534990125	2.29519826101805
H	2.95356968637412	-1.97476924871180	1.69892851907682
H	4.03580633234071	0.85946457014106	1.52055257139941
H	2.44667013477876	0.28172589064843	2.00991160710558
H	1.44586406618583	2.06866052194465	-0.94127112719104
H	1.63944886818714	2.08756149414660	0.82211368393817
H	4.74161596286355	0.59106181472823	-1.02058613961824
H	4.04482033053017	2.14165039144272	-0.50450417453323
H	3.46624334171158	1.35335902168944	-1.99698738514524
